# Supplementary material for: Cultural adaptation of a psychosocial screening tool for adolescents living with HIV/AIDS attending antiretroviral therapy program in Malawi
Source: PLoS One. 2025 Nov 17;20(11):e0318738. doi: 10.1371/journal.pone.0318738 (PMC12622793; doi:10.1371/journal.pone.0318738)
Supplement: S1 File — English Focus Group Discussion Guide. S2 Text. Chichewa Focus Group Discussion Guide. S3 Text. Original HEADSS tool. S4 Text. Participants HEADSS adaptation notes_v1. S5 Text. HEADSS adaptation v1. S6 Text. Participants HEADSS adaptation notes_ v2. S7 Text. HEADSS adaptation v2. S8 Text. HEADSS adaptation v3. S9 Text. HEADSS adaptation _v4_Final Version. (ZIP) [file pone.0318738.s001.zip › Supporting Information/Supplementary File 1.docx]

**Supplementary File 1 - English Focus Group Guide**

**Study Title: Cultural adaptation of a psychosocial screening tool for adolescents living with HIV/AIDS attending antiretroviral therapy program in Malawi**

| Topic | Comments |
| --- | --- |
| Introduction | - Facilitator introduces herself & Explain briefly the objectives of the discussions |
| Concession | - Adolescents Living with HIV (ALHIV) |
| Objectives of the FG | - The objectives of the focus group discussion are - Health and Adolescents - Perceived benefits of HEADSS Screening/Assessment |
| Participants consent | - The facilitator explains on consent and that the participants can ask for any clarification at any time |
| Introduce the participants | - Use an ice breaker to relax the group, FG can be stressful for participants, especially if the participants do not know each other at all |

***Comprehensibility of the adapted HEADSS tool***

1. What do you think of this adapted HEADSS tool overall?
2. In your opinion, are the questions in the HEADSS tool clear and understandable?
3. May you tell me, if the language in this tool is appropriate to Malawi setting?
4. What if any aspects of the screening tool do you find useful, and how?
5. In general, how would you describe the design of HEADSS screening tool and its quality?
6. Do you find the questions in the screening tool easy to understand or somehow complex?
7. Do you have any suggestions for how the tool could be improved?

***Acceptability of the adapted tool***

1. Are you finding the design of the HEADSS tool acceptable? Why or why not?
2. Do you think the wording in this tool is acceptable to Malawi setting? Why or why not?
3. What aspects of the tool, do you find helpful and why?
4. What aspects of the tool, do you not find helpful and why?
5. What aspects of this tool should be changed to make it acceptable?
6. Please explain to me, how we can improve this tool to make it more acceptable?

***Relevance of the adapted tool***

1. In your opinion, do you think the questions on this tool are important to ALHIV needs and if yes, why so?
2. Which sections do you think are most relevant and why so or why not?
3. Do you find the questions in the tool appropriate? Why or why not?
4. Do you have any suggestions for how the tool could be improved?

***General questions***

1. Do you have anything you want to add or clarify or any questions?
2. Is there anything important that you expected to discuss that we have not covered?

**Wrap –Up:**

**Thank the participants and summarize the discussion and how it will help inform the study**

Any observations/comments by interviewer

| Comments |
| --- |
|  |

***THANK YOU VERY MUCH FOR YOUR TIME***
