# Supplementary material for: Cultural adaptation of a psychosocial screening tool for adolescents living with HIV/AIDS attending antiretroviral therapy program in Malawi
Source: PLoS One. 2025 Nov 17;20(11):e0318738. doi: 10.1371/journal.pone.0318738 (PMC12622793; doi:10.1371/journal.pone.0318738)
Supplement: S1 File — English Focus Group Discussion Guide. S2 Text. Chichewa Focus Group Discussion Guide. S3 Text. Original HEADSS tool. S4 Text. Participants HEADSS adaptation notes_v1. S5 Text. HEADSS adaptation v1. S6 Text. Participants HEADSS adaptation notes_ v2. S7 Text. HEADSS adaptation v2. S8 Text. HEADSS adaptation v3. S9 Text. HEADSS adaptation _v4_Final Version. (ZIP) [file pone.0318738.s001.zip › Supporting Information/Supplementary File 2.docx]

Supplementary File 2 - Chichewa Focus Group Guide

**Mutu wa Kafukufuku*: Kusintha mwatsatanetsane chida chowunikira m'maganizo a ngwiro mwa achinyamata omwe ali ndi kachilombo ka HIV ndi Edzi mu pulogalamu ya achinyamata ya ma ARV m’boma la Zomba, m’Malawi***

Muli bwanji, dzina langa ndi.................................Ndine opanga kafukufuku yemwe watchulidwa pamwambapa kuchokera ku university of North Carolina, Lilongwe.

| Mutu wa Nkhani | Ndemanga |
| --- | --- |
| Mawu woyamba | - Wotsogolera zokambirana alifotokozere gulu kuti iwo ndi ndani komanso zolinga zazokambirana zimenezi ndi zotani |
| Chilolezo | - Tengani Chilolezo kwa achinyamata |
| Zolinga zokambirana pa gulu | - Fotokozani mwachidule zolinga za zokambirana za pa gulu - Zolinga za zokambirana zimenezi ndi:   - Umoyo wa achinyamata   - Kupindula kowunikiridwa/kufufuzidwa matenda okhudza za maganizo |
| Kupempha mamembala a gulu kuti achite nawo zokambirana | - Fotokozani kwa mamembala a gulu kuti akhoza kepempha wotsogolera kuti afotokoze bwinobwino pamene iwo sakumvetsa, nthawi ina iliyonse |
| Ma membala a gulu anene maina awo ndi kumene amachokera | - Gwiritsani ntchito nthabwala mosataya nthawi kuti mamembala a gulu asamatope ndizokambirana. Zokambirana zotere zimakhala zotopetsa makamaka ngati ma membala a gulu sadziwana ndi komwe |

***KUMVETSETSA KWA CHIDA CHOSINTHIDWA CHA HEADSS***

1. Mukuganiza bwanji za chida chosinthidwa cha HEADSS chonsechi?
2. Mukuganiza kwanu, kodi mafunso omwe ali pachida cha HEADSS kodi ndi omveka bwino?
3. Mungandiuzeko, ngati chilankhulo chomwe chili mchida ichi ndi mchoyenera ku Malawi kuno?
4. Kodi ndi zinthu ziti pachidachi zomwe mukuwona kuti ndizothandiza, ndipo ndizothandiza motani?
5. Kodi mungafotokoze bwanji za muonekedwe a chida cha HEADSS ndi kagwiritsidwe kake ka ntchito?
6. Kodi mukuona kuti mafunso omwe ali mu chidachi ndiwosavuta kumvetsetsa kapena ndi wovuta?
7. Kodi muli ndi malingaliro aliwonse amomwe chidacho chingasinthidwire kuti chigwire ntchito moyenerera?

***KUVOMEREZEKA KWA CHIDA CHOSINTHIDWACHO***

1. Kodi mukuwona maonekedwe a chida cha HEADSS ali ovomerezeka? Chifukwa chiyani?
2. Kodi mukuganiza kuti mawu omwe ali mchida ichi ndi ovomerezeka ku Malawi kuno? Chifukwa chiyani?
3. Ndi mbali ziti za chida, zomwe mukuwona kuti ndizothandiza ndipo chifukwa chiyani?
4. Nanga ndi mbali ziti za chida, zomwe simukuziwona nzothandiza komanso chifukwa chiani?
5. Ndi zinthu ziti za chida ichi zomwe zikuyenera kusinthidwa kuti zikhale zovomerezeka?
6. Chonde ndifotokozereni, momwe tingasinthire chida ichi kuti chikhale chovomerezeka?

***KUFUNIKA KWAKE***

1. Mukuganiza kwanu, mukuganiza kuti mafunso omwe ali pachidachi ndi ofunikira pa zomwe ALHIV amafuna ndipo ngati inde, ndichifukwa chiyani?
2. Kodi ndi zigawo ziti zomwe mukuganiza kuti ndizofunikira kwambiri ndipo mchifukwa chiyani zili choncho kapena bwanji ziri choncho?
3. Kodi mukuwona mafunso omwe ali pachidacho kukhala oyenera? Chifukwa chiyani?
4. Kodi muli ndi malingaliro ali wonse amomwe chida ichi chingasinthidwire kuti chigwire bwino ntchito?

***MAFUNSO OWONJEZERA***

1. Kodi muli ndi chili chonse chomwe mukufuna kuwonjezera kapena kufotokoza kapena mafunso aliwonse?
2. Kodi pali chinthu china chofunikira chomwe mumayembekezera kukambirana zomwe sitinaziphimbe/sitinazikambe?

Kumaliza (Wrap –Up):

Thokozani onse potenga nawo mbali pa mchezowu. Mosataya nthawi kambani za momwe mchezowu wayendela ndi ubwino wa zokambiranazo mu kafukufuku ameneyu? Pa nthawi ino, musakambepo zimenene mwa kambirana.

1. Ndemanga iliyonse kwa ofunsa mafunso

| Ndemanga |
| --- |
|  |

***Zikomo Kwambiri***
