# Supplementary material for: Cultural adaptation of a psychosocial screening tool for adolescents living with HIV/AIDS attending antiretroviral therapy program in Malawi
Source: PLoS One. 2025 Nov 17;20(11):e0318738. doi: 10.1371/journal.pone.0318738 (PMC12622793; doi:10.1371/journal.pone.0318738)
Supplement: S1 File — English Focus Group Discussion Guide. S2 Text. Chichewa Focus Group Discussion Guide. S3 Text. Original HEADSS tool. S4 Text. Participants HEADSS adaptation notes_v1. S5 Text. HEADSS adaptation v1. S6 Text. Participants HEADSS adaptation notes_ v2. S7 Text. HEADSS adaptation v2. S8 Text. HEADSS adaptation v3. S9 Text. HEADSS adaptation _v4_Final Version. (ZIP) [file pone.0318738.s001.zip › Supporting Information/Supplementary File 4.docx]

**Supplementary File 4 – Participants HEADSS Adaptation notes _v1**

| **DOMAIN** | **SUB-HEADING** | **CLINIC 1: ALHIV RESPONSES** | **CLINIC 2: ALHIV RESPONSES** |
| --- | --- | --- | --- |
| **HOME & ENVIRONMENT** | **RELATIONSHIP AT HOME** |  |  |
|  | Mungandiwuze dzina lanu ndi komwe mumakhala? | Should remain the same | Should remain the same |
|  | Mumakhala ndi ndani? (Makolo okubelekani, a zakhali, amalume kapena agogo ndi abale anu ena?) | Mumakhala ndi ndani ? (exclude examples in brackets) | Should remain the same |
|  | Mungandiwuze zambiri za achibale anuwa? | Mumakhala bwanji ndi achibale anuwa?  (Majority agreed to remove this question because they think it is the same as the following question) | Mungatiwuze za chikhalidwe cha abale anuwo?  Mungandiuze zabwino kapena zoipa za achibale anuwa? |
|  | Mumakhalitsana motani ndi achibale anuwa? | (Should replace question 3)  This one should go up to question 3 | Kodi achibale anu amakuwonetserani makhalidwe abwino?  Kodi ndi makhalidwe otani amene amakuwonetserani abale anuwa.? |
|  | Kodi mumakhala mosangalala, mwa mtendere ndi movomerezedwa m’banja mwanu? | Should remain the same | Should remain the same |
|  | Munayamba mwathawapo kunyumba komwe mumakhalako? Ngati munathawako, ndi chifukwa chiyani munathawa ndipo munapita kukakhala kuti? | Should remain the same | Should remain the same |
|  | Kodi alipo wina akumwanso mankhwala mbanja mwanu? | Should remain the same | Should remain the same |
|  |  |  |  |
|  | **STRESS** |  |  |
|  | Zimachitika kuti nthawi zina munthu umatha kupanikizika m’maganizo, kukhala odandaula ndi zochitika. Munayamba mwakhalapo odandaula? | Should remain the same | Should remain the same |
|  | Mungandifotokozereko mavuto amene amakusowetsani mtendere pakhomopo? | Should remain the same | Should remain the same |
|  | Kodi mumakhala odandaula mu zinthu zambiri ndikuona kuti nkhawa zanu sizikuchoka? ndi kudandaulanso kwa nthawi yayitali? | (The question too long should be split  Or in short, they suggested that it should just be as follows)  Kodi mumakhala odandaula kwa nthawi yayitali bwanji? | Should remain the same |
|  | Kodi mumamukhuthukira ndani mukakhala ndi nkhawa kapena mukapanikizika mmaganizo? | Kodi mungamuuze ndani mukakhala ndi nkhawa kapena mukapanikizika m’maganizo? | Kodi mumauza ndani mukakhala ndi nkhawa kapena kupanikizika m’maganizo? |
|  |  |  |  |
|  | **PHYSICAL ABUSE** |  |  |
|  | Mchaka chathachi, mwapangidwako nkhanza kapena kumenyedwa ndi wina aliyense? | Mwapangidwako nkhanza kuchokera zaka za m’buyozi kufikira pano ndi wina aliyense? | Should remain the same |
|  | Mungafotokoze m’mene nkhazazo zinachitikira? | Should remain the same | Should remain the same |
|  |  |  |  |
|  | **ADHERENCE TO ANTIRETROVIRAL THERAPY** |  |  |
|  | Kupatula anzanu kuno ku Teen Club ndi aku banja kwanu, munawuzapo anthu ena za kuti muli ndi kachilombo ka HIV? | Should remain the same | Should remain the same |
|  | Amene amakulimbikitsani ndani zakutsata ndondomeko ya kamwedwe ka mankhwala anu? | Should remain the same | Should remain the same. Only add Kodi at the beginning.  Kodi ndi ndani amene amakulimbikitsani zakutsata ndondomeko ya kamwedwe ka mankhwala anu? |
|  | Mumadumphitsa kumwa mankhwala anu mowirikiza bwanji? | Kodi kamwedwe ka mankhwala anu kamakhala motani, modumphitsa kapena ayi? | Kodi munayamba mwakhalapo nthawi yayitali bwanji musanamwe mankhwala anu? |
|  | Nanga mukakhala ku sukulu, mamwedwe anu a makhwala amakhala otani? (kwa okhawo ali ku sukulu yogonera konko) | Should remain the same | kodi mukakhala ku sukulu yogonera konko kamwedwe ka mankhwala anu kamankhala kotani? |
|  |  |  |  |
|  | **STIGMA AND DISCRIMINATION** |  |  |
|  | Kodi mumadzimva kuti mukusankhidwa, kutonzedwa kapena kusalidwa munjila ina iliyonse chifukwa muli ndi kachilombo ka HIV (kudela kwanu, kunyumba kapena kusukulu) | Kodi mumadziwa kuti mukutonzedwa kapena kutsalidwa munjira inailiyonse? | Should remain the same |
|  | Kodi mumakumana ndi mavuto anji, kamba ka zoyankhula za anthu ku sukulu kapena kunyumba? | Kodi mukukumana ndi mavuto anji ochokera kunyumba kapena kusukulu? | Should remain the same |
|  | Pali zosintha za m’thupi mwanu zokhudzana ndi kumwa mankhwala zomwe zikukudandaulitsani? (ngati kukula mabele kwa achinyamata kapena kusintha nkhope*)* | Should remain the same | Should be the same |
|  |  |  |  |
|  | **FOOD SECURITY** |  |  |
|  | Mungandifotokozereko zakudya zomwe mumanya patsiku; m’mawa , masana ndi madzulo | Should remain the same | Should remain the same. Only add kodi at the beginning:  Kodi Mungandifotokozereko zakudya zomwe mumanya patsiku; m’mawa , masana ndi madzulo? |
|  | Kodi mumadya zakudya zomwe inu mukufuna? | Kodi mumakwanitsa kupeza zakudya zomwe inu mukufuna? | Should remain the same |
|  | Kodi makolo anu zakudya zimenezi amapeza bwanji? | Should remain the same | Should remain the same |
|  |  |  |  |
| **EDUCATION AND EMPLOYMENT** |  |  |  |
|  | Kodi muli pa sukulu? | Should remain the same | Should remain the same |
|  | Kodi muli ku pulayimale, sekondale kapena sukulu ya ukachenjede/yoyendera kapena yogonera pompo? | Should remain the same | Should remain the same |
|  | Kodi amakulipilirani fizi, kukugulirani uniform ndi zofunikira ku sukulu ndani? | Kodi amakugulirani zofunika ku sukulu ndi ndani? | Should remain the same |
|  | Kodi mmakumana ndi zotani ku sukulu? (kuzunzidwapo/kumenyedwapo) | Should remain the same | Should remain the same |
|  | Kodi munabwerezapo kalasi, kuyimitsidwa kapena kuchotsedwa? Chifukwa chiyani? | Should remain the same | Should remain the same |
|  | Kodi mwajombako ku sukulu mwezi uno, miyezi itatu yapitayi kapena teremu yathayi? (pali zifukwa zanji?) | Should remain the same | Should remain the same |
|  | Munayamba mwalingalirapo zosiya sukulu? | Should remain the same | Should remain the same |
|  | Pali wina wake amene mumakhala omasuka naye ku sukulu, yemwe mumakamba naye zinthu zofunikira? (ndipo ndi ndani?) | Should remain the same | Should remain the same |
|  | Kodi mumafuna kudzapanga chiyani mukamaliza sukulu. Muli ndi malingaliro anji atsogolo lanu pa ntchito yomwe mumafuna kudzagwira? | Question must be split, because it is too long.  Kodi mumafuna kudzapanga chiyani mukamaliza sukulu?  Muli ndi malingaliro anji atsogolo lanu pa ntchito yomwe mumafuna kudzagwira? | Should remain the same |
|  | Kodi mumagwira ntchito ina iliyonse mukakhala kuti simuli ku sukulu? Ngati eeeh kutiko? Mumalipilidwa kapena ayi? | Should remain the same | Should remain the same |
|  | Kodi mumakhalitsana nawo bwanji amene anakulembani ntchitowo? | Should remain the same | Should remain the same |
| **ACTIVITIES** |  |  |  |
|  | Kodi Mumachita chiyani pa nthawi yanu yopuma?( monga kupita ku magulu a achinyamata, masewera olimbitsa thupi ngati mpira wa miyendo ndi wa manja) | Should remain the same | Should remain the same |
|  | Anzinzanu ambiri muli nawo ndi ochokera kusukulu kapena madela ena? (ali ndi zaka zingati, ndi anyamata kapena atsikana, nanga amakonda chiyani?) | Should remain the same | Should remain the same |
|  | Kodi mumapita ku tchalitchi pafupi pafupi kapena kutenga nawo gawo pa zochitika za mu tchalitchi? | Should remain the same | Should remain the same |
|  | Kodi pali nthawi zina zomwe mumakhala muli nokha nokha? Nanga mumaona kuti zili bwino bwino? | Should remain the same | Should remain the same |
|  | Kodi pali nthawi yomwe mumakhala osungulumwa kapena kusalidwa pa magulu a anzanu pazochitika? Chimachitika ndi chani ndipo mumamva bwanji? | Should remain the same | Should remain the same |
| **DRUG AND SUBSTANCE ABUSE** |  |  |  |
|  | Kodi mukudziwapo chiani za mankhwala ozunguza bongo? | Should remain the same | Should remain the same |
|  | Kodi mudayamba mwagwiritsapo ntchito, kapena anzanu ena anayamba agwiritsapo ntchito mankhwala ozunguza bongowa? (monga marijuana, Kuber, Indian hemp,mowa) | Question too long needs to be split and remove examples in brackets:  Kodi munayamba mwagwitsapo ntchito mankhawala ozunguza bongowa? The question should only end there. | Should remain the same |
|  | Ndi chifukwa chiyani anthu amatenga mankhwala ozunguza bongowa? | Should remain the same | Should remain the same |
| **SEXUAL AND REPRODUCTIVE HEALTH** |  |  |  |
|  | **Munayamba mwawamverapo anzanu ena kuti amagonana ndi abwenzi awo?** | Should remain the same | Should remain the same |
|  | Nanga inuyo, kodi munayamba mwakhalapo ndi chibwenzi chomwe mumagonana nacho ndipo ndinu omasuka nazo? | Remove nanga inuyo, should be:  Kodi munayamba mwakhalapo ndi chibwenzi chomwe mumagonana nacho ndipo ndinu omasuka? | Kodi munayamba mwakhalapo ndi chibwenzi ndipo munayambapo mwakhalapo pamodzi ? |
|  | Ngati munayamba mwakhalapo pa chibwenzi, mwagonapo ndi anthu angati? | Should remain the same | Should remain the same |
|  | Pali wina anakuchitanipo nkhanza? Anakuchitani nkhanza motani ndipo munachitapo chiyani? | This question should be removed and placed on abuse section. | Same. Add Kodi at the beginning. Kodi pali wina anakuchitanipo nkhanza ankuchitani nkhanza motani ndipo munachitapo chiyani? |
|  | Munayamba mwafotokozerapo abwenzi anu kuti muli ndi kachilombo ka HIV? | Should remain the same | Add Kodi.  Kodi munayamba mwafotokozerapo abwenzi anu kuti muli ndi kachilombo ka HIV? |
|  | Nanga abwenzi anu anakuwuzani ngati ali ndi kachilombo koyambitsa HIV kapena ayi? | Add at the beginning ngati muli ndi chibwenzi , the question should flow as follows:  Ngati muli ndi chibwenzi , abwenzi anu anakuwuzani ngati ali ndi kachilombo koyambitsa HIV kapena ayi? | Should remain the same |
|  | Munayamba mwagonanapo ndi munthu ndi cholinga choti akupatseni ndalama kapena zinthu zimene mumasowa? | Should remain the same | Add kodi at the beginning:  Kodi munayamba mwagonanapo ndi munthu ndi cholinga choti akupatseni ndalama kapena zinthu zimene mumasowa? |
|  | Ngati munayamba mwagonapo ndi abwenzi anu, munatulukapo ukazi kapena umuna omwe udakudabwitsani kuti mwina mwatenga matenda? | Should remain the same | Should remain the same |
|  | Kodi mukudziwapo chiyani pa nkhani yogonana modziteteza? | Should remain the same | Should remain the same |
|  | Munayamba mwamvapo za ma condom kapena njira zina zodzitetezera ku matenda opatsirana pogonana kapena mimba? | Should remain the same | Add Kodi.  Kodi munayamba mwamvapo za ma condom kapena njira zina zodzitetezera ku matenda opatsilana pogonana kapena mimba? |
|  | Munayamba mwagwiritsako ntchito njira zomwe mwatchulazo? | Should remain the same | Add Kodi.  Kodi munayamba mwagwiritsako ntchito njira zomwe mwatchulazo? |
|  | Kodi pali wina wake anakugwirani thupi lanu mosayenera/musakufuna, kapena kugonana nanu kumene musakufuna? | Should remain the same | Should remain the same |
|  | Ngakhale mwakamba kuti simunakhalepo ndi chibwenzi, muli ndi chidwi ndi anyamata kapena atsikana. (Kapena simunaganizire za nkhaniyi, kapena munachita chisankho chosala kaye). **(Kwa amene sanayambe zibwenzi).** | Should remain the same | Should remain the same.  Only po chosala kaye pakhale kudziletsa kaye.  Ngakhale mwakamba kuti simunakhalepo ndi bwenzi muli ndi chidwi ndi anyamata kapena atsikana.(Kapena simunaganizire za nkhaniyi, kapena munachita chisankho chodziletsa kaye).(Kwa amene sanayambe zibwenzi). |
|  |  |  |  |
| **SUICIDE /DEPRESSION** |  |  |  |
|  | Munayamba mwakhalako mu mmaganizo oterawa. | Should remain the same | Should remain the same. Add kodi at the beginning:  Kodi Munayamba mwakhalako mu mmaganizo oterawa? |
|  | Zimachitika pafupi pafupi bwanji, ndipo kwa nthawi yayitali bwanji? | Should remain the same | Kodi kukhumudwako kumachitika pafupi pafupi bwanji, ndipo kwa nthawi yayitali bwanji? |
|  | Kodi mumakhala ndi maganizo obwelera mbuyo pafupi pafupi? N’chifukwa chani? | Should remain the same | Should remain the same |
|  | Kodi pali nthawi ina yomwe simumafuna kukhala ndi anzanu, kapena achibale anu kwa nthawi yayitali? N’chifukwa chiyani zili choncho? | Should remain the same | Should remain the same |
|  | Kodi muli ndi khalidwe lililonse limene limakulepheretsani kukhala pakati pa achibale kapena anzanu kusukulu, ngakhale m’dera lanu (mwachitsanzo, kukangana kangana)? | Should remain the same | Should remain the same |
|  | Kodi mumadya bwanji, mumagona bwanji komanso mumalimbikitsidwa bwanji? | Should remain the same | Should remain the same |
|  |  |  |  |
|  |  |  |  |
|  | Kodi munayamba mwadzipwetekapo nokha kapena kuganiza zopweteka munthu wina (mwachitsanzo kudzicheka, kudziwotcha, kapena kudzikanda) | Should remain the same | Should remain the same |
|  | Mwa achinyamata, Kodi mukudziwa wina amene anadzipha chifukwa chovutika maganizo? | Should remain the same | Kodi mukudziwa wina mwa achinyamata, amene anadzipha chifukwa chovutika mmaganizo? |

Supplementary File 4 2
