# Supplementary material for: Cultural adaptation of a psychosocial screening tool for adolescents living with HIV/AIDS attending antiretroviral therapy program in Malawi
Source: PLoS One. 2025 Nov 17;20(11):e0318738. doi: 10.1371/journal.pone.0318738 (PMC12622793; doi:10.1371/journal.pone.0318738)
Supplement: S1 File — English Focus Group Discussion Guide. S2 Text. Chichewa Focus Group Discussion Guide. S3 Text. Original HEADSS tool. S4 Text. Participants HEADSS adaptation notes_v1. S5 Text. HEADSS adaptation v1. S6 Text. Participants HEADSS adaptation notes_ v2. S7 Text. HEADSS adaptation v2. S8 Text. HEADSS adaptation v3. S9 Text. HEADSS adaptation _v4_Final Version. (ZIP) [file pone.0318738.s001.zip › Supporting Information/Supplementary File 5.docx]

**Supplementary File 5 - HEADSS adaptation v1**

VERSION 1_ADAPTED HEADSS PSYCHOSOCIAL SCREENING TOOL FOR ADOLESCENTS LIVING WITH HIV IN TEEN CLUB PROGRAM


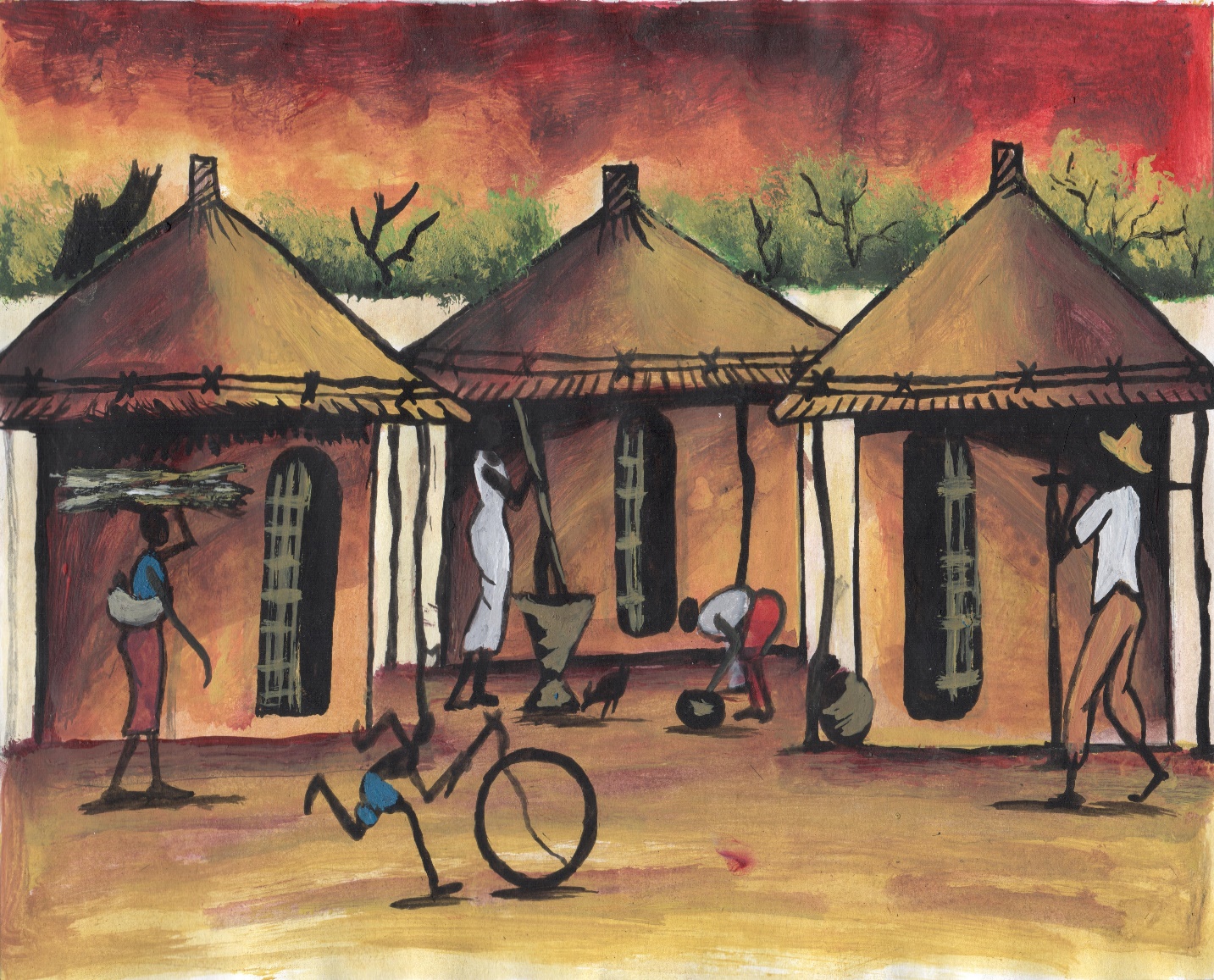


| DOMAINS | INTERVIEWS QUESTIONS |
| --- | --- |
| **H – HOME AND ENVIRONMENT** | |
| **ENGLISH** | **CHICHEWA** |
| **RELATIONSHIPS AT HOME** | **UBALE KU NYUMBA** |
| May I know your name and where you stay? | Mungandiuze dzina lanu ndi komwe mumakhala? |
| Who do you live with? | Nanga mumakhala ndi ndani? |
| How do you get along with your relatives? | Nanga inuyo mumakhala nawo bwanji achibale anu? |
| What challenges do you face from where you live? | Ndi zovuta zanji zomwe mumakumana nazo kuchokera kumene mukukhala? |
| Have you ever had thoughts of leaving your home? | Munayamba mwakhalapo ndi maganiza ochoka komwe mukukhala? |
| Why did you have such an idea? | N’chifukwa chiyani munali ndi maganizo amenewa? |
| Is there anyone on ARVs where you live? | Kodi pali wina aliyense amene ali pa ma ARV komwe mumakhala? |
| How about in your family, is anyone on ARVs apart from you? | Nanga m’banja mwanu pali wina aliyense amene amamwa ma ARV kupatula inu? |


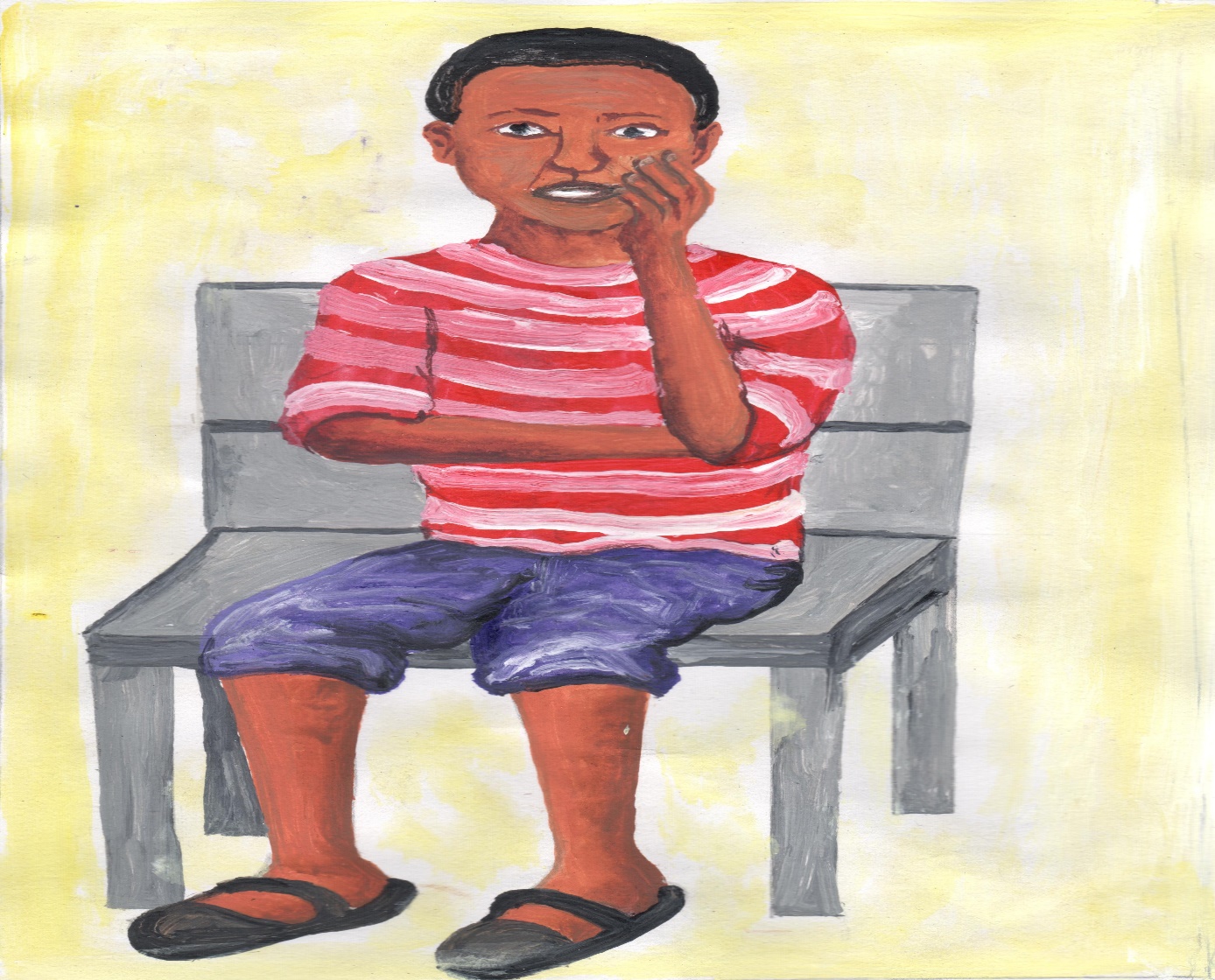


| **STRESS/ANXIETY** | **KUKHALA NDI NKHAWA** |
| --- | --- |
| ***Sometimes people get stressed up by what is happening around them, have you ever been stressed up?*** | ***Nthawi zina munthu amatha kukhala okhumudwa ndi zochitika za m’mene akukhalira ndi achibale ake. Inu munayamba mwakhumudwapo komanso kuda nkhawa?*** |
| Do you know what anxiety is? | Kodi mumatha kuzindikira kuti nkhawa ndi chiani? |
| Have you ever felt disappointed and worried by the way your family or community members treat you? What are your worries? | Kodi munayamba mwakhumudwapo ndi kuda nkhawa ndi mmene achibale anu kapena anthu a m’dera lanu akukuchitirani? Kodi nkhawa zanu ndi zotani? |
| Please tell me what you are worried about? | Tandiuzani mukuda nkhawa ndi chani? |
| In particular, what makes you anxious? | Makamaka, chimakupangitsani kuti mukhale ndi nkhawa ndi chiani? |
| May you explain what can cause a person to be depressed or anxious | Tafotokozani zomwe zingapangitse munthu kuti akhale okhumudwa kapena ndi nkhawa |
| When you get worried, what do you do? | Mukakhala ndi nkhawa mumatani? |
| Is there a time when you are alone and how do you feel when you are alone? | Kodi pali nthawi yomwe muli nokha ndipo mumamva bwanji mukakhala nokha? |
| Who do you talk to at home when you are worried? | Mumafotozera ndani kunyumba mukakhala ndi nkhawa? |


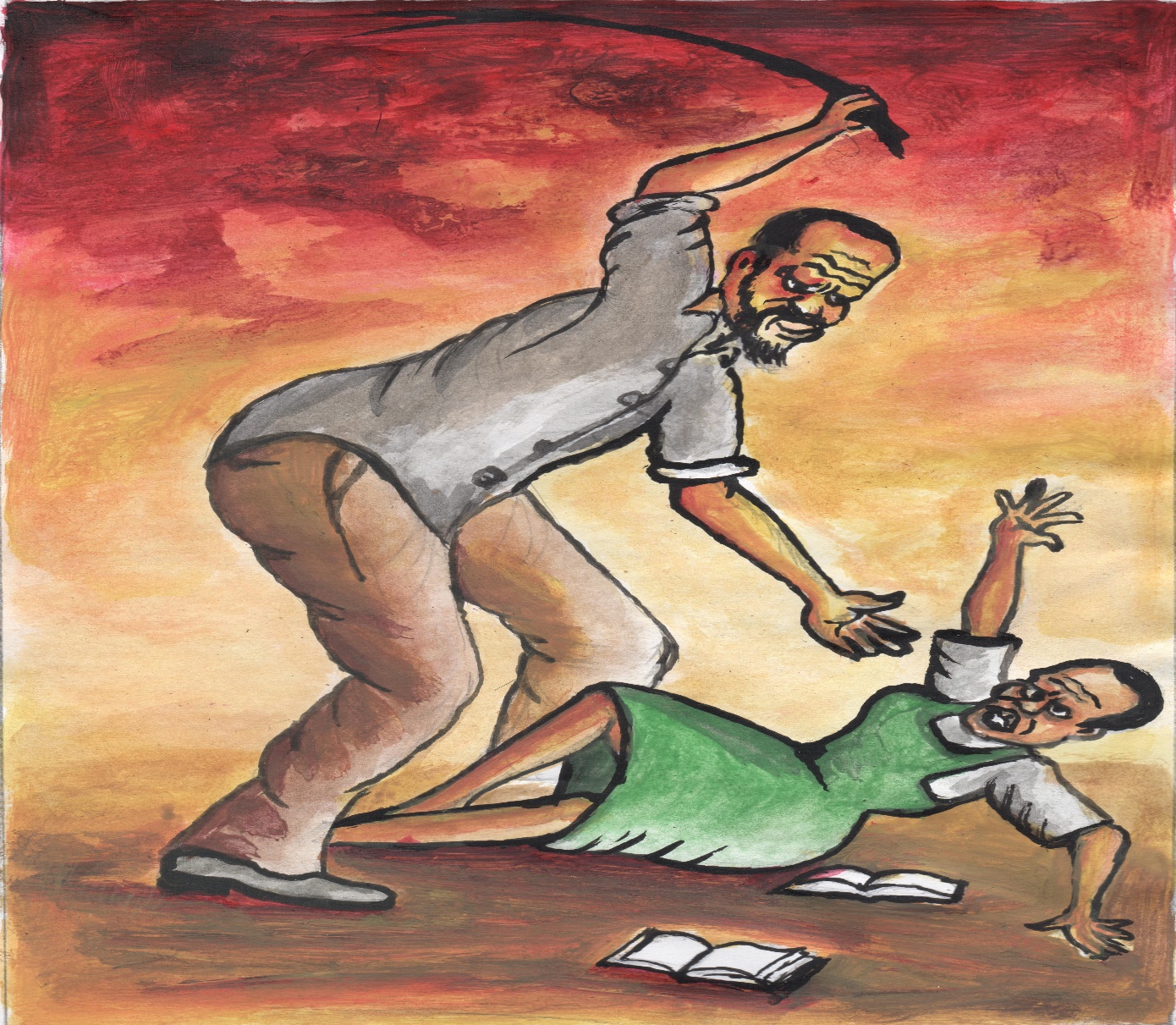


| **ABUSE** | **NKHANZA** |
| --- | --- |
| Have you ever been a victim of violence? | Kodi munayamba mwachitidwapo nkhanza? |
| Explain what kind of violence it was | Tafotokozani zinali nkhanza za mtundu wanji? |
| What did you do after being abused? | Munatani mutachitiridwa nkhanza? |
| Where did you report after being abused? | Kodi mudakchita lipoti kutiko mutachitiridwa nkhanza? |
| Has anyone ever forced to have sex with you without your consent? | Kodi pali wina amene anakugwililiranipo kapena kukukakamizani kuti agonane nanu popanda chilolezo chanu? |


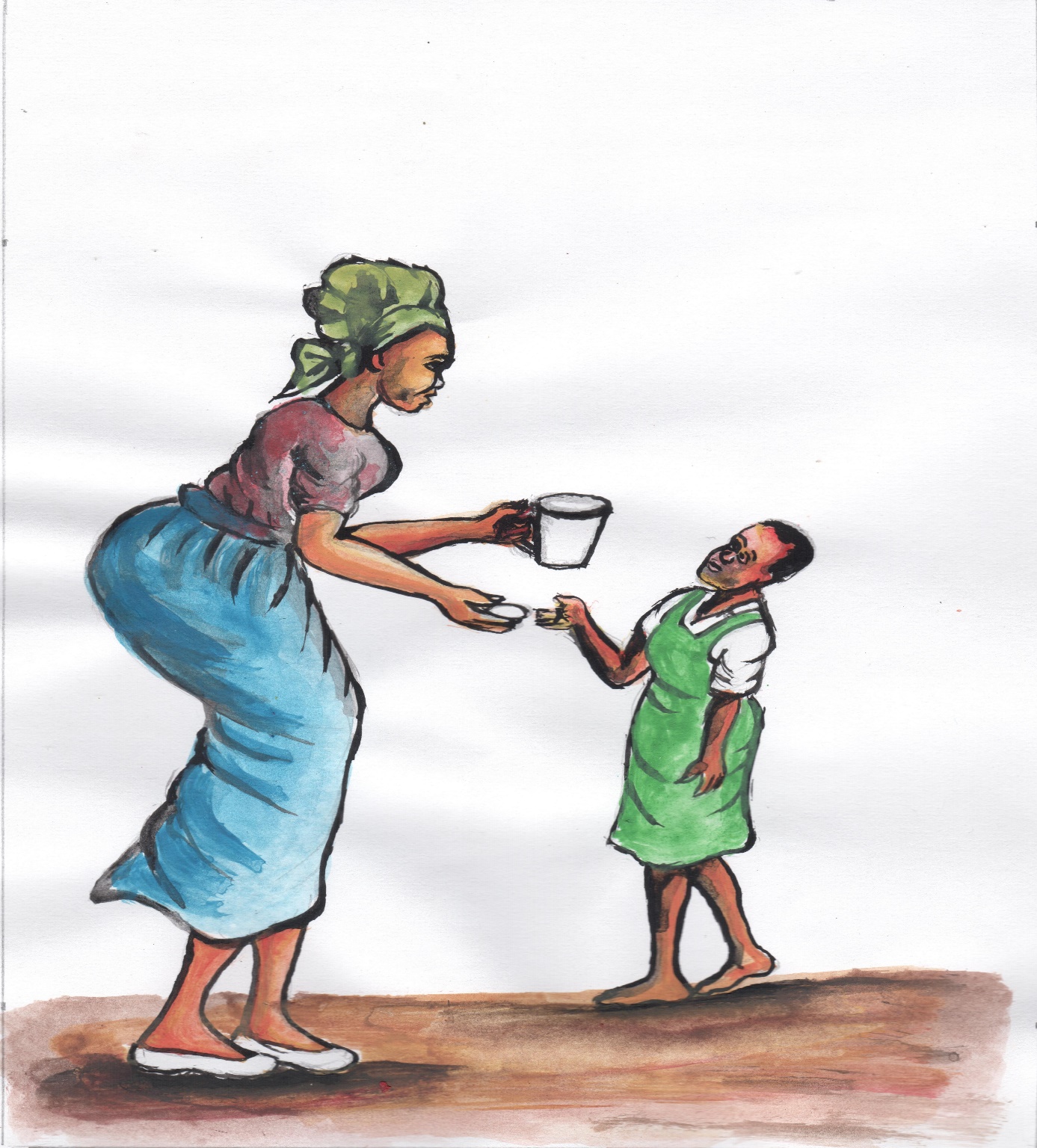


| **ADHERENCE TO ANTIRETROVIRAL THERAPY** | **KUMWA MANKHWALA MWA NDONDOMEKO** |
| --- | --- |
| Apart from your friends in Teen Club or your family, have you disclosed your HIV status to other people? | Kupatula anzanu kuno ku Teen Club ndi akubanja kwanu, munawuzapo anthu ena za kuti muli ndi kachilombo ka HIV? |
| Who supports/encourages you to take your medications/ARVs? | Ndani amakuthandizani/amakulimbikitsani kumwa mankhwala/ma ARV? |
| Do you ever forget to take medication/ARVs? | Kodi pali nthawi ina iliyonse yomwe mumayiwala kumwa mankhwala/ma ARV? |
| What caused you to forget to take medication? | Chinachitika ndi chiyani kuti muiwale kumwa mankhwala? |
| Who reminded you to take your medicine? | Anakukumbutsani ndi ndani kuti mumw mankhwala anu? |
| How do you manage to take your medications at school? (*Only for those in boarding schools*) | kodi mukakhala ku sukulu yogonera konko kamwedwe ka mankhwala anu kamakhala kotani? |
| Did you disclose your HIV status to anybody (*for those in boarding schools)?* | Kodi mudauzako aliyense kuti muli ndi kachilombo ka HIV (*kwa omwe ali kusukulu zogonera*)? |
| Do you have any problems when taking the drugs? | Muli ndi vuto lililonse mukamamwa mankhwalawa? |
|  |  |


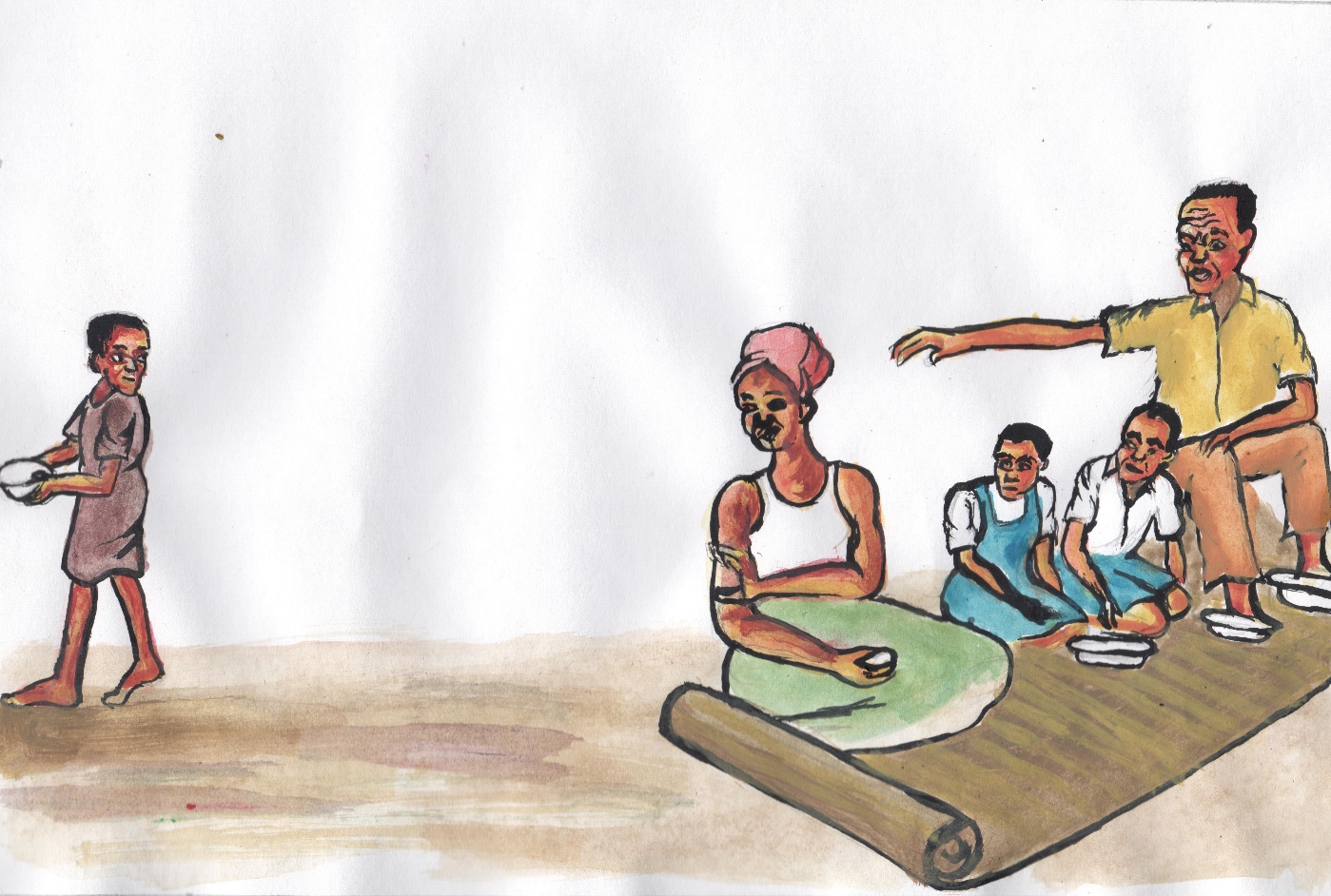


| **STIGMA AND DISCRIMINATION** | **KUSALIDWA** |
| --- | --- |
| Do you feel discriminated, being bullied and stigmatized in any way because you are HIV positive?(in the community, home or at school)? | Kodi mumadzimva kuti mukusankhidwa, kutonzedwa kapena kusalidwa munjila ina iliyonse chifukwa muli ndi kachilombo ka HIV (kudela kwanu, kunyumba kapena kusukulu) |
| What difficulties/problems do you face because of what other people say about you at school or at home? | Ndizovuta ziti zomwe mumakumana nazo chifukwa cha zomwe anthu ena amanena za inu kusukulu kapena kunyumba? |
| How do you feel about being on ARVs? | Mumamva bwanji kuti muli pa ma ARV? |
| Do you feel discriminated against during community or school events? | Kodi mumaona kuti mumakusalidwa panthawi ya zochitika za m'dera lanu kapena kusukulu? |
| What happens and how do you feel? | Kodi chimachitika ndi chiyani ndipo mukumva bwanji? |


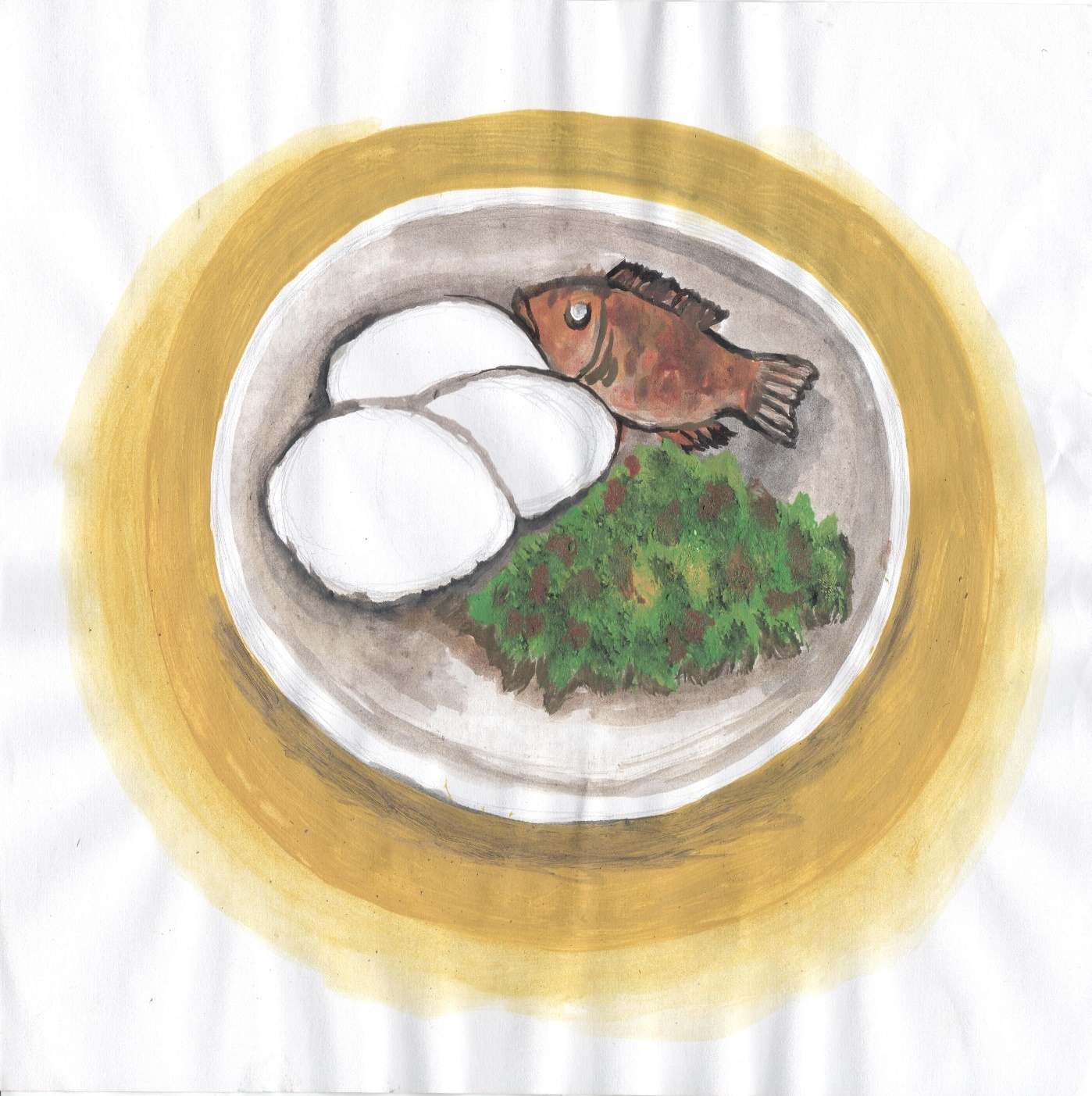


| **FOOD SECURITY** | **KUKHALA NDI CHAKUDYA CHOKWANIRA** |
| --- | --- |
| How many times do you take food in a day? | Kodi mumadya chakudya kangati pa tsiku? |
| What type of foods do you take for breakfast, lunch and supper? | Ndi zakudya zotani zomwe mumadya m'mawa, masana ndi madzulo? |
| How do your parents get adequate food to feed the whole family? | Kodi makolo anu amapeza bwanji chakudya chokwanira banja lonse? |


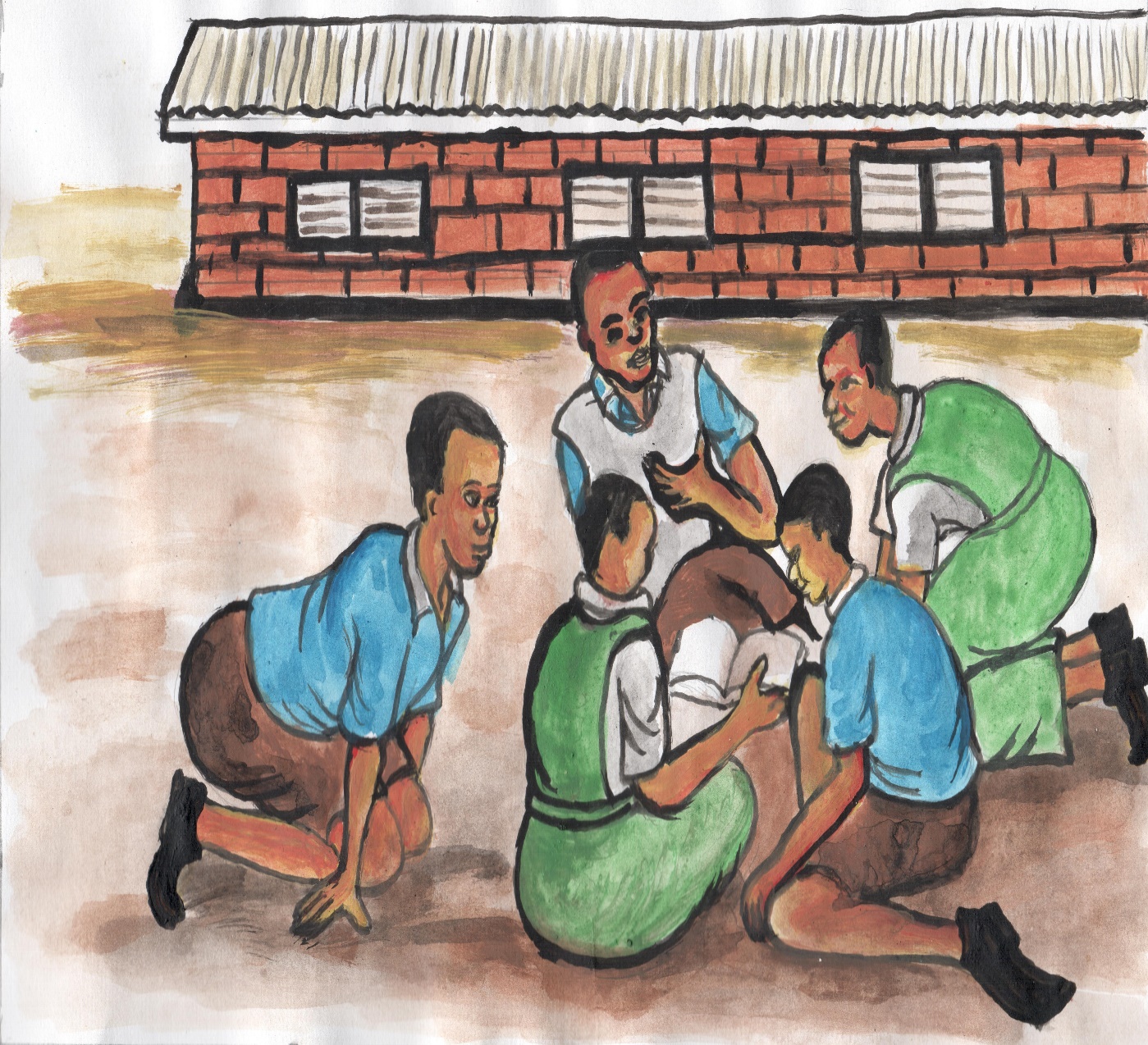


| **E – EDUCATION AND EMPLOYMENT/MAPHUNZIRO NDI NTCHITO/GANYU** | |
| --- | --- |
| Are you in school? If yes, where do you go to school? | Kodi muli pasukulu? Ngati inde, mumapita ku sukulu itiyo? |
| Who pays for your books and other expenses | Ndi ndani amakulipirani ndalama za mabuku ndi zina zofunikira ku sukulu? |
| What problems do you face because you are at school? | Kodi mumakumana ndi mavuto otani chifukwa choti muli pa sukulu? |
| So how do you deal with these problems? | Ndiye mumathana ndi mavutowa bwanji? |
| Have you ever been disciplined at school? | Munayamba mwapatsidwapo chilango ku sukulu? |
| What type of the punishment did you get? | Kodi munalandira chilango chotani? |
| Have you been absent from school and if yes, why? *(Ask for the past 3 months)* | Munayamba mwajombapo ku sukulu? Ndi chifukwa chiani? (*Funsani kwa miyezi itatu yapitayi*) |
| Have you ever considered dropping out of school? | Kodi munayamba mwaganizapo zosiya sukulu? |
| If yes, why did you consider of dropping out of school? | Ngati inde, n’chifukwa chiyani munaganiza zosiya sukulu? |
| When you are at school, who is your confidant that you discuss freely about your HIV status and the ARVs you are taking? | Mukakhala kusukulu, ndani amene mumamukhulupirira yemwe mumakambirana momasuka za momwe mulili ndi kachilombo ka HIV komanso ma ARV omwe mukumwa? |
| What do you want to do when you finish school? | Kodi mumafuna kudzapanga chiyani mukamaliza sukulu? |
| What future plans do you have regarding your career? | Muli ndi malingaliro anji atsogolo lanu pa ntchito yomwe mumafuna kudzagwira? |
|  |  |
| **FORMAL/INFORMAL EMPLOYMENT /KUGWIRA NTCHITO/GANYU** | |
| How much are you paid when you do some work for some people or in a company? | Kodi mumalipidwa ndalama zingati mukamagwira ntchito kwa anthu ena kapena pakampani? |
| How do you get along with the ones who employed you (give you piece works) | Mumagwirizana bwanji ndi omwe adakulembani ntchito (akupatsani ntchito) |
| Are you comfortable to work or doing piece works? | Ndinu womasuka kugwira ntchito kapena kugwira ganyu? |
|  |  |
|  |  |
|  |  |


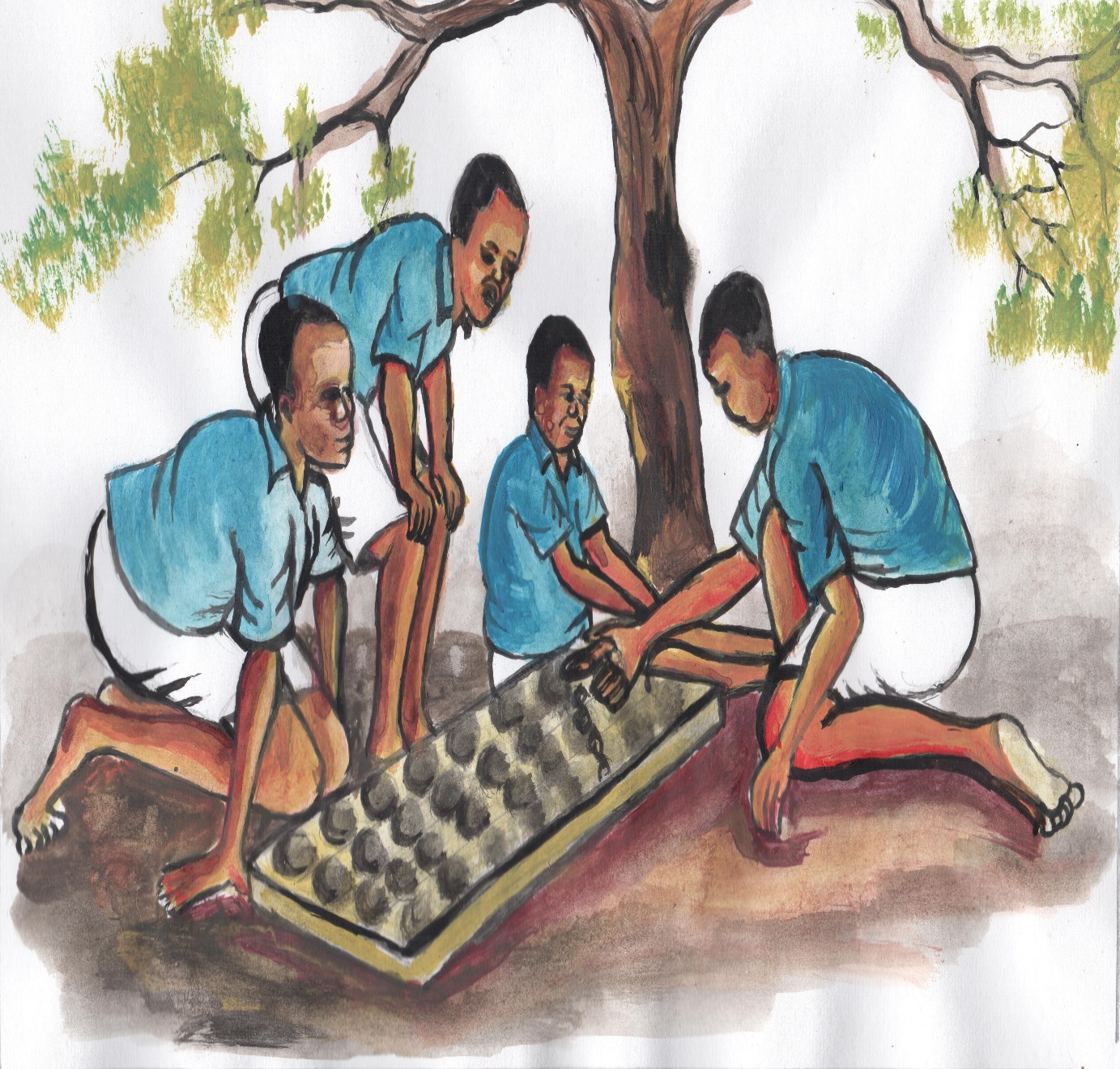


| **A – ACTIVITIES/ZOCHITA** | |
| --- | --- |
| Do you have friends and where do they come from? | Kodi muli ndi anzanu ndipo amachokera kuti? |
| What types of games do you usually play? | Ndi masewera anji omwe mumakonda kusewera? |
| What religion do you belong to? | Ndinu achipembedzo chanji? |
| Do you have time to go for prayers? | Kodi muli ndi nthawi yopita kukapemphera? |
| Mumamva bwanji mukakhala m’nyumba yopemphereramo? | How do you feel when you are in the chapel/prayer house? |
| Does going to pray give you encouragement and hope | Kodi kupita kukapemphera kumakupatsani chilimbikitso ndi chiyembekezo? |
| Do you participate in church or mosque activities? | kodi mumatenga nawo gawo pa zochitika za mu tchalitchi kapena ku nzikiti? |


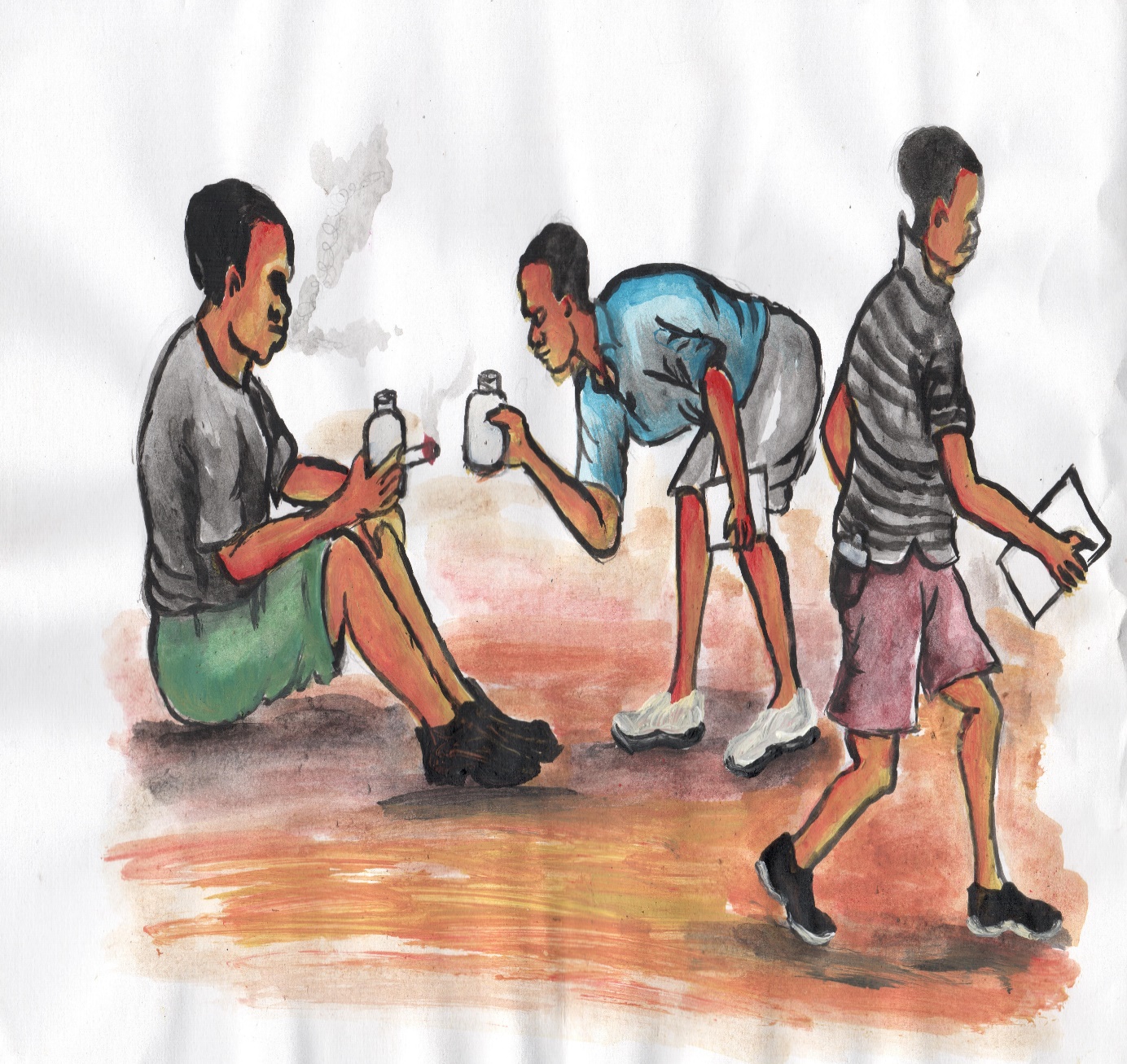


| **D – DRUGS USE AND ABUSE/MANKHWALA OZUNGUZA BONGO** | |
| --- | --- |
| ***Some young people abuse substances/drugs*** | ***Achinyamata ena amagwiritsa ntchito mankhwala ozunguza bongo*** |
| Do you understand what substance abuse mean? | Kodi mukumvetsa tanthauzo la kugwiritsa ntchito mankhwala ozunguza bongo? |
| Can you give me examples of addictive drugs found in your community? | Kodi mungandipatseko zitsanzo za makhwala ozunguza bongo omwe amapezeka m’deral la kwanu? |
| Do you know the negative effects of combining such drugs with ARVs? | Kodi mukudziwa kuipa kophatikiza mankhwalawa ndi ma ARV? |
| Do you have friends who abuse drugs (alcohol, marijuana, cigarettes)? | Kodi muli ndi anzanu omwe amagwiritsa ntchito mankhwala osokoneza bongo (*mowa, chamba, fodya*)? |
| How about you, have you ever abused drugs? | Nanga inu, munagwiritsapo ntchito mankhwala ozunguza bongo? |
| Why do people abuse drugs? | N’chifukwa chiyani anthu amagwiritsa ntchito mankhwala ozunguza bongowa? |
| How can one avoid abusing drugs? | Kodi munthu angapewe bwanji kugwiritsa ntchito mankhwala ozunguza bongowa? |


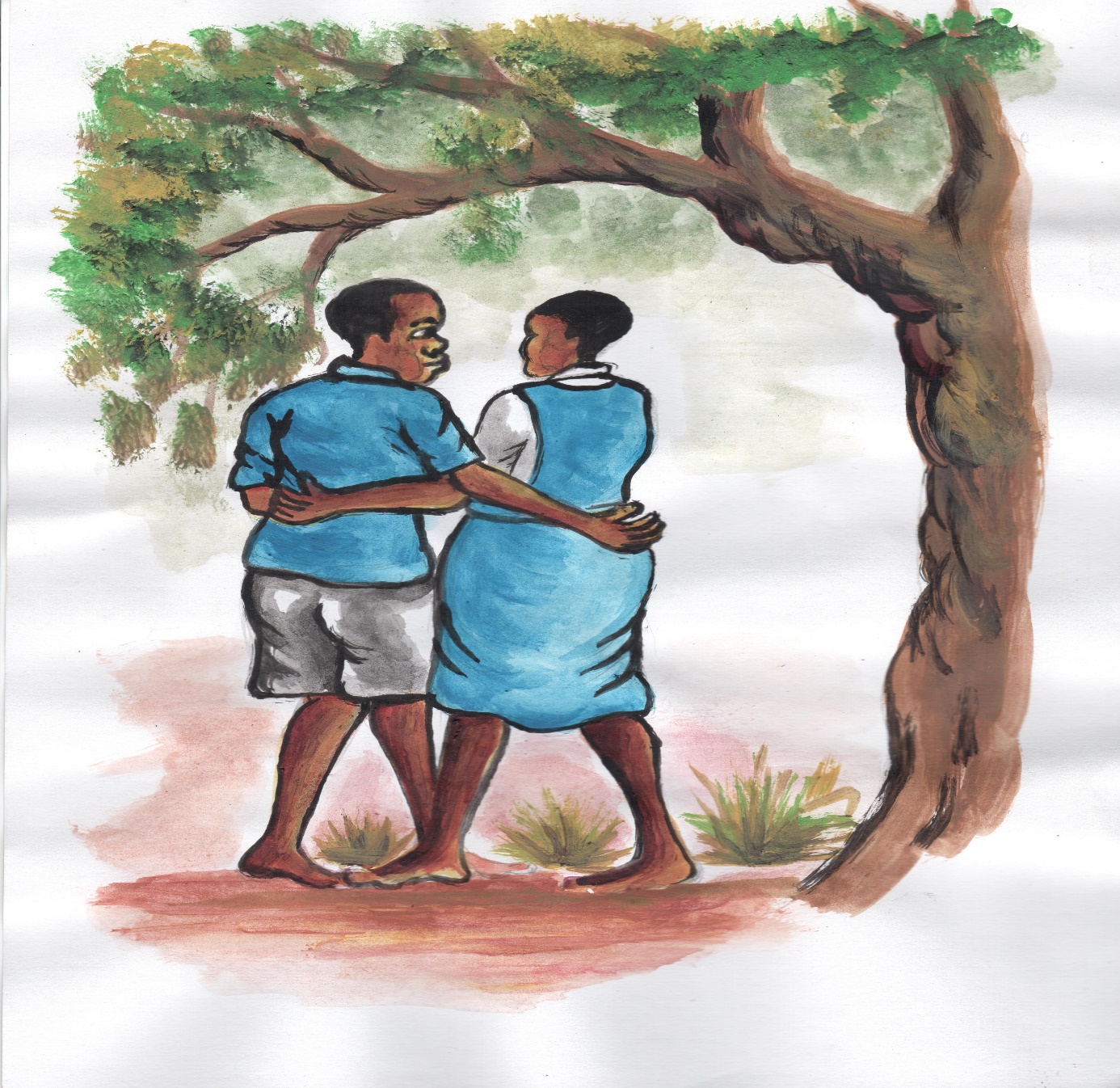


| **S-SEXUAL AND REPRODUCTIVE HEALTH/KUGONANA NDI UBELEKI WABWINO** | |
| --- | --- |
| ***Some young people are involved in intimate relationships*** | ***Achinyamata ena amachita nawo maubwenzi apamtima*** |
| Do you know how your body works? | Kodi mumadziwa m'mene thupi lanu limagwilira ntchito? |
| Have you ever been asked to date someone? | Kodi munafunsidwapo kuti mukhale pachibwenzi? |
| Was that person same age as you or older than you? | Kodi munthu ameneyo anali wamsinkhu wofanana ndi inu kapena wamkulu kuposa inu? |
| If you've been in a relationship before, how did you feel about it? | Ngati munayamba mwakhalapo pachibwenzi, zinakusangalatsani bwanji? |
| If you have never been in a relationship before, has someone ever touched your private parts? | Ngati simunakhalepo pachibwenzi, pali wina amene anakugwirapo ziwalo zobisika? |
| Have you ever heard of anyone among your friends having a sexual relationship? | Munayamba mwawamverapo anzanu ena kuti amagonana ndi chibwenzi chawo? |
| How about you, have you ever had any sexual relationship with someone else? | Nanga inu, munayamba mwagonanapo ndi munthu wina? |
| Have you ever exchanged sex with money or material things? | Munayamba mwagonanapo ndi munthu ndi cholinga choti akupatseni ndalama kapena zinthu zimene mumasowa? |
| If you have had a sexual relationship with someone else, how many sexual partners have you had? | Ngati munagonanapo ndi munthu wina, kodi munagonanapo ndi anthu angati? |
| If you have a sexual partner, have you ever discussed about HIV and AIDS? | Ngati muli ndi chibwenzi chogonana nacho, munayamba mwakambiranapo za HIV ndi Edzi? |
| Have you ever discussed that you are HIV positive and that you are taking ARVs? | Kodi mudakambiranapo kuti muli ndi kachilombo ka HIV komanso kuti mukumwa ma ARV? |
| Have you ever accessed information on safer sex? | Kodi mudamvapo nkhani yogonana modziteteza? |
| Have you ever discussed with your sexual partner about protecting yourselves from STIs and pregnancy? | Munayamba mwakambiranapo ndi chibwenzi chanu zodziteteza ku matenda opatsirana pogonana komanso kutenga mimba? |
| If you have ever received advice on sexual and reproductive health, and how to protect yourself, where did you get this information? | Ngati mudalandirapo uphungu wokhudzana ndi zakugonana ndi ubeleki, komanso ndi m'mene mungadzitetezele munalandira kutiko? |
| Have you ever heard of family planning methods? | Munayamba mwamvapo za njira za kulera? |
| Who should use these methods? | Ndipo oyenera kugwiritsa njirazi ndi ndani? |
| How can you find family planning methods? | Kodi njira za kulera mungazipeze bwanji? |
| Even if you say you have never been in a relationship do you have an interest in boys or girls? | Ngakhale mwanena kuti simunakhalepo ndi chibwenzi kodi muli ndi chidwi ndi anyamata kapena atsikana? |
| Are you thinking about marriage in the future? | Kodi mumaganiza zodzakhala pa banja m'tsogolomu? |


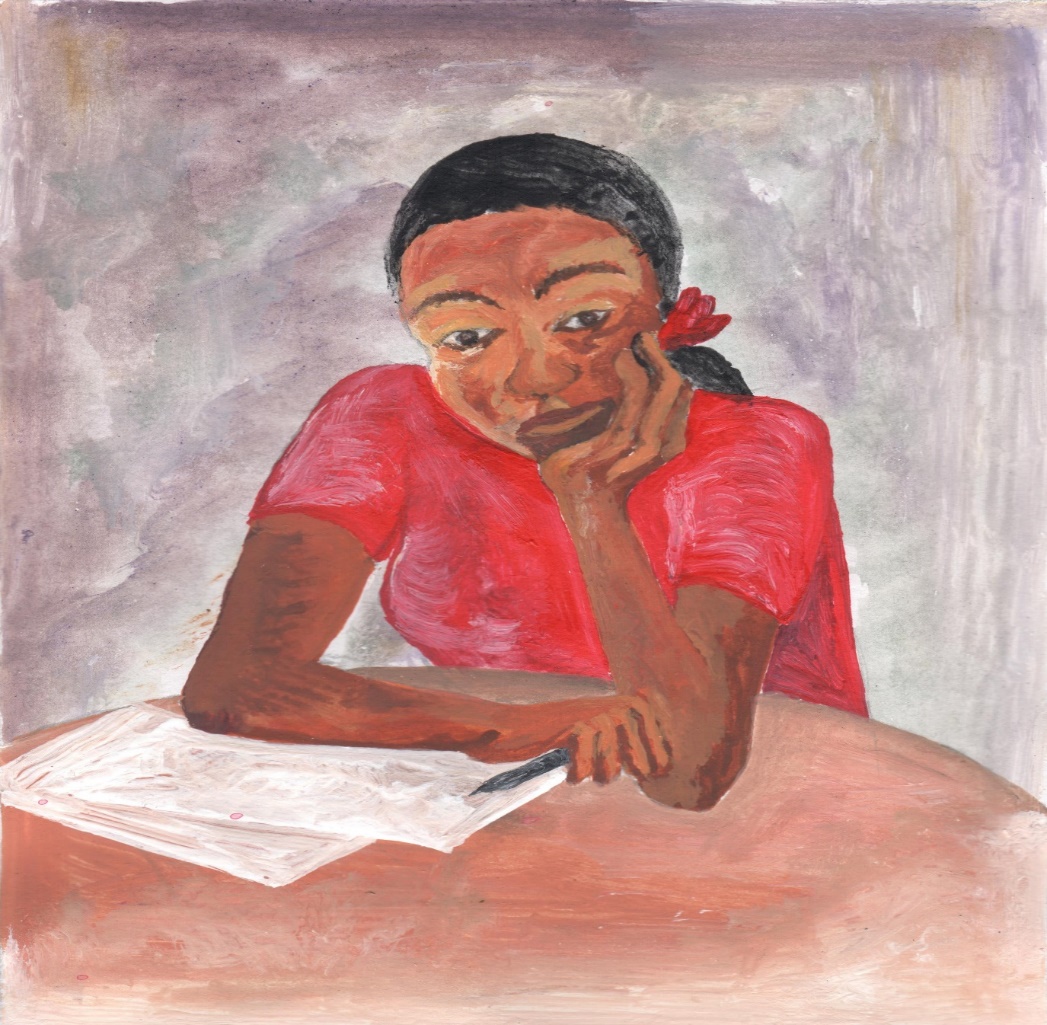


| **S - SUICIDE/DEPRESSION/KUDZIPHA NDI KUKHUMUDWA** | |
| --- | --- |
| ***Some people become so depressed that they want to ruin their lives*** | ***Anthu ena amavutika m’maganizo kwambiri moti amafuna kuwononga moyo wawo*** |
| Have you ever intentionally hurt yourself (*for example cutting, burning, scratching*) | Kodi munadzivulaza mwadala (*mwachitsanzo kudzicheka, kudzitentha, kudzikanda)* |
| Many young people feel discouraged, sad and low/not happy | Achinyamata ambiri amakhumudwa, achisoni komanso osasangalala |
| Have you ever been depressed? So, what did you do? | Kodi munayamba mwavutikapo maganizo? Nanga munachitapo chiani? |
| How do you feel about taking ARVs for the rest of your life? | Kodi mumamva bwanji kuti mukumwa ma ARV m'moyo mwanu wonse? |
| Have you ever thought that you are different from your peers and that things are not going well because you are on ARVs? | Munayamba mwaganizapo kuti ndinu osiyana ndi anzanu ndipo zinthu sizikuyenda bwino chifukwa mumamwa ma ARV? |
| As a result of taking ARVs, do you feel that it is better to just leave the world? If so, why? | Chifukwa chokumwa ma ARV, kodi mumakhala ndi maganizo oti kuli bwino kungochoka pa dziko pano? Ngati ndi choncho, n'chifukwa chiani? |
| Are you able to talk to your friends about the challenges you are facing, and do they encourage you to lead a better life? | Kodi mumatha kukambirana ndi anzanu za zovuta zomwe mukukumana nazo ndipo amakulimbikitsani kuti mukhale ndi moyo wabwino? |
| Have you ever felt depressed after an argument with family and friends? Why was that? | Kodi munayamba mwakhumudwapo pambuyo pokangana ndi achibale kapena anzanu? N’chifukwa chiyani zinali choncho? |
| When you get depressed do you want to continue your daily routine/activities? | Kodi mukakhumudwa mumafuna kupitiliza zomwe mumachita tsiku ndi tsiku? |
| Have you ever heard of a young person committing suicide and why? | Kodi munamvapo kuti wachinyamata wadzipha ndipo chifukwa chiyani? |
| What about you? Have you ever thought that you do not want to live in this world anymore? | Nanga inu? Kodi munayamba mwaganizirapo kuti simukufunanso kukhala m’dziko lino? |
| Who did you tell about such thoughts? | Munamuuza ndani za maganizo oterewo? |
|  |  |
