# Supplementary material for: Cultural adaptation of a psychosocial screening tool for adolescents living with HIV/AIDS attending antiretroviral therapy program in Malawi
Source: PLoS One. 2025 Nov 17;20(11):e0318738. doi: 10.1371/journal.pone.0318738 (PMC12622793; doi:10.1371/journal.pone.0318738)
Supplement: S1 File — English Focus Group Discussion Guide. S2 Text. Chichewa Focus Group Discussion Guide. S3 Text. Original HEADSS tool. S4 Text. Participants HEADSS adaptation notes_v1. S5 Text. HEADSS adaptation v1. S6 Text. Participants HEADSS adaptation notes_ v2. S7 Text. HEADSS adaptation v2. S8 Text. HEADSS adaptation v3. S9 Text. HEADSS adaptation _v4_Final Version. (ZIP) [file pone.0318738.s001.zip › Supporting Information/Supplementary File 6.docx]

**Supporting File 6 Participants HEADSS adaptation responses v2**

| **DOMAIN** | **ADAPTATION VERSION v1** | | **CLINIC 1: ALHIV RESPONSES** | **CLINIC 2: ALHIV RESPONSES** | **HCPS AND KIS RESPONSES** |
| --- | --- | --- | --- | --- | --- |
| **H - HOME** | **HOME & ENVIRONMENT** | | | | |
| Original HEADSS Questions | **RELATIONSHIP AT HOME** | | | | |
|  | **CHICHEWA** | **ENGLISH** |  |  |  |
| Where do you live? | Mungandiwuze dzina lanu ndi komwe mumakhala? | May I know your name and where you stay? | Should remain the same | Should remain the same | Mungandiuze dzina lanu ndi komwe mumakhala? |
| Who do you live with? | Mumakhala ndi ndani? (Makolo okubelekani, a zakhali, amalume kapena agogo ndi abale anu ena?) | Whom do you stay with? (Biological parents, an auntie, uncle or grandparents and other relatives/siblings?) | Mumakhala ndi ndani? (exclude examples in brackets)  *Majority agreed to remove this question because they think it is the same as the following question)* | Should remain the same | Nanga mumakhala ndi ndani? |
| What do you and your family argue about? | Mumakhalitsana motani ndi achibale anuwa? | How do you get along with your relatives? | (Should replace question 3) | Mungandiuze zabwino kapena zoipa za achibale anuwa? | Should remain the same |
| How much time do you spend at home? | Kodi mumakhala mosangalala, mwa mtendere ndi movomerezedwa m’banja mwanu? | Do you live with them happily, in peace and feeling part of your family? | Should remain the same | Should remain the same | Ndi zovuta zanji zomwe mumakumana nazo kuchokera kumene mukukhala? |
| Have you ever run away from home? | Munayamba mwathawapo kunyumba komwe mumakhalako? Ngati munathawako, ndi chifukwa chiyani munathawa ndipo munapita kukakhala kuti? | Have you ever run away from your home? If yes, why did you do so and where did you go to stay? | Should remain the same | Should remain the same | Munayamba mwakhalapo ndi maganiza ochoka komwe mukukhala? |
|  | Kodi alipo wina akumwanso mankhwala mbanja mwanu? | Is there anybody in your family, who is on ART? | Should remain the same | Should remain the same | Kodi pali wina aliyense amene ali pa ma ARV komwe mumakhala? |
|  | **SUB-HEADINGS** | |  |  |  |
|  | **STRESS** | |  |  |  |
|  | Zimachitika kuti nthawi zina munthu umatha kupanikizika m’maganizo, kukhala odandaula ndi zochitika. Munayamba mwakhalapo odandaula? | Sometimes people get stressed up by what is happening around them, have you ever been stressed up? | Should remain the same | Should remain the same | Nthawi zina munthu amatha kukhala okhumudwa ndi zochitika za m’mene akukhalira ndi achibale ake. Inu munayamba mwakhumudwapo komanso kuda nkhawa? |
|  | Mungandifotokozereko mavuto amene amakusowetsani mtendere pakhomopo? | Can you explain to me the problems that make you get stressed at home? | Should remain the same | Should remain the same | Tafotokozani zomwe zingapangitse munthu kuti akhale okhumudwa kapena ndi nkhawa |
|  | Mungandifotokozereko mavuto amene amakusowetsani mtendere pakhomopo? | Can you explain to me the problems that make you get stressed at home? | Should remain the same | Should remain the same | Should remain the same |
|  |  |  |  |  |  |
|  | Kodi mumakhala odandaula mu zinthu zambiri ndikuona kuti nkhawa zanu sizikuchoka? ndi kudandaulanso kwa nthawi yayitali? | Do you worry a lot about things and find the worry just won’t go away? | (The question too long and recommended that we should split it)  Kodi mumakhala odandaula kwa nthawi yayitali bwanji? | Should remain the same | Should remain the same |
| Can you go to your parents with problems? | Kodi mumamukhuthukira ndani mukakhala ndi nkhawa kapena mukapanikizika mmaganizo? | Who do you talk to at home when you are stressed? | Kodi mungamuuze ndani mukakhala ndi nkhawa kapena mukapanikizika m’maganizo? | Kodi mumauza ndani mukakhala ndi nkhawa kapena kupanikizika m’maganizo? | Mumafotozera ndani kunyumba mukakhala ndi nkhawa? |
|  | **PHYSICAL ABUSE** | |  |  |  |
|  | Mchaka chathachi, mwapangidwako nkhanza kapena kumenyedwa ndi wina aliyense? | In the past year, have you ever been physically abused or been beaten by somebody?  If yes, can you explain how the abuse happened? | Mwapangidwako nkhanza kuchokera zaka za m’buyozi kufikira pano ndi wina aliyense? | Should remain the same | Should remain the same |
|  | **ADHERENCE TO ANTIRETROVIRAL THERAPY** | |  |  |  |
|  | Kupatula anzanu kuno ku Teen Club ndi aku banja kwanu, munawuzapo anthu ena za kuti muli ndi kachilombo ka HIV? | Apart from your friends in Teen Club or your family, have you disclosed your HIV status to other people? | Should remain the same | Should remain the same | Kupatula anzanu kuno ku Teen Club ndi akubanja kwanu, munawuzapo anthu ena za kuti muli ndi kachilombo ka HIV? |
|  | Amene amakulimbikitsani ndani zakutsata ndondomeko ya kamwedwe ka mankhwala anu? | Who supports/encourages you to take your medications/ARVs? | Should remain the same | Should remain the same. Only add “Kodi” at the beginning e.g.,  Kodi ndi ndani amene amakulimbikitsani zakutsata ndondomeko ya kamwedwe ka mankhwala anu? | Ndani amakuthandizani/amakulimbikitsani kumwa mankhwala/ma ARV?  ? |
|  | Mumadumphitsa kumwa mankhwala anu mowirikiza bwanji? | How often do you miss taking your medications/ARVs? | Kodi kamwedwe ka mankhwala anu kamakhala motani, modumphitsa kapena ayi? | Kodi munayamba mwakhalapo nthawi yayitali bwanji musanamwe mankhwala anu? | Should remain the same |
|  | Nanga mukakhala ku sukulu, mamwedwe anu a makhwala amakhala otani? (kwa okhawo ali ku sukulu yogonera konko) | How do you manage taking your medications/ARVs at school? (Only for those in boarding schools) | Should remain the same | kodi mukakhala ku sukulu yogonera konko kamwedwe ka mankhwala anu kamakhala kotani? | Kodi mumakwanitsa bwanji kumwa mankhwala kusukulu? (*Kwa omwe ali m'sukulu zogonera okha*) |
|  | **STIGMA AND DISCRIMINATION** | |  |  |  |
|  | Kodi mumadzimva kuti mukusankhidwa, kutonzedwa kapena kusalidwa munjila ina iliyonse chifukwa muli ndi kachilombo ka HIV (kudela kwanu, kunyumba kapena kusukulu) | Do you feel discriminated, being bullied and stigmatized in any way because you are HIV positive?(in the community, home or at school) | Kodi mumadziwa kuti mukutonzedwa kapena kutsalidwa munjira inailiyonse? | Should remain the same | Should remain the same |
|  | Kodi mumakumana ndi mavuto anji, kamba ka zoyankhula za anthu ku sukulu kapena kunyumba? | What difficulties/problems do you face because of what other people say about you at school or at home? | Kodi mukukumana ndi mavuto anji ochokera kunyumba kapena kusukulu? | Should remain the same | Should remain the same |
|  | Pali zosintha za m’thupi mwanu zokhudzana ndi kumwa mankhwala zomwe zikukudandaulitsani? (ngati kukula mabele kwa achinyamata kapena kusintha nkhope) | Are there any changes in your body that have resulted from taking the ARVs that you are worried about? (Lipodystrophy or gynecomastia)? | Should remain the same | Should remain the same | Should remain the same |
|  | **FOOD SECURITY** | |  |  |  |
|  | Mungandifotokozereko zakudya zomwe mumanya patsiku; m’mawa , masana ndi madzulo | May you explain to me, what type of foods do you normally eat in a day; breakfast, lunch, and supper? | Kodi mumakwanitsa kupeza zakudya zomwe inu mukufuna? | Should remain the same but add “kodi”  For example: Kodi Mungandifotokozereko zakudya zomwe mumadya patsiku; m’mawa , masana ndi madzulo? | Should remain the same |
|  | Kodi mumadya zakudya zomwe inu mukufuna? | Do you normally eat what you want at home? | Kodi mumakwanitsa kupeza zakudya zomwe inu mukufuna? | Should remain the same | Should remain the same |
|  | Kodi makolo anu zakudya zimenezi amapeza bwanji? | How do your parents find the food in the home? | Should remain the same | Should remain the same | Kodi makolo anu amapeza bwanji chakudya chokwanira banja lonse? |
| **E - EDUCATION AND EMPLOYMENT** | |  |  |  |  |
| What grade are you in? | Kodi muli pa sukulu? | Are you in school? | Should remain the same | Should remain the same | Should remain the same |
| What grades are you getting? Have they changed? | Kodi muli ku pulayimale, sekondale kapena sukulu ya ukachenjede/yoyendera kapena yogonera pompo? | Are you in primary school, secondary school or tertiary institution? Are you in boarding or not? | Should remain the same | Should remain the same | Should remain the same |
|  | Kodi amakulipilirani fizi, kukugulirani uniform ndi zofunikira ku sukulu ndani? | Who pays for your school fees, uniform, and school materials? | Kodi amakugulirani zofunika ku sukulu ndi ndani? | Should remain the same | Should remain the same |
| Have you ever been teased or attacked at school? | Kodi mumakumana ndi zotani ku sukulu? (kuzunzidwapo/kumenyedwapo) | Do you face any problems at school?? (Inquire about “bullying”). | Should remain the same | Should remain the same | Kodi mumakumana ndi mavuto otani chifukwa choti muli pa sukulu? |
| Have you ever failed any classes or been kept back a grade? | Kodi munabwerezapo kalasi, kuyimitsidwa kapena kuchotsedwa? Chifukwa chiyani? | Have you ever repeated a class, suspended or expelled from school? Why did this happen? | Should remain the same | Should remain the same | Should remain the same |
| Do you ever skip classes? | Kodi mwajombako ku sukulu mwezi uno, miyezi itatu yapitayi kapena teremu yathayi? (pali zifukwa zanji?) | Have you been absent from school this month, three months ago or the previous term? (what was the reason) | Should remain the same | Should remain the same | Munayamba mwajombapo ku sukulu? Ndi chifukwa chiani? (*Funsani kwa miyezi itatu yapitayi*) |
|  | Munayamba mwalingalirapo zosiya sukulu? | Have you ever considered dropping out of school? | Should remain the same | Should remain the same | Kodi munayamba mwaganizapo zosiya sukulu? |
|  | Pali wina wake amene mumakhala omasuka naye ku sukulu, yemwe mumakamba naye zinthu zofunikira? (ndipo ndi ndani?) | Do you have anybody at school whom you freely discuss important issues with? (Who is he/she)? | Question must be split, because it is too long.  “Kodi mumafuna kudzapanga chiyani mukamaliza sukulu?” | Should remain the same | Mukakhala kusukulu, ndani amene mumamukhulupirira yemwe mumakambirana momasuka za momwe mulili ndi kachilombo ka HIV komanso ma ARV omwe mukumwa? |
| What are your career/vocational goals? | Kodi mumafuna kudzapanga chiyani mukamaliza sukulu. Muli ndi malingaliro anji atsogolo lanu pa ntchito yomwe mumafuna kudzagwira? | What do you want to do when you finish school?  What future plans do you have regarding your career? | Question must be split, because it is too long.  Kodi mumafuna kudzapanga chiyani mukamaliza sukulu?  Muli ndi malingaliro anji atsogolo lanu pa ntchito yomwe mumafuna kudzagwira? | Should remain the same | Kodi mumafuna kudzapanga chiyani mukamaliza sukulu? |
| Do you work after school or on weekends? | Kodi mumagwira ntchito ina iliyonse mukakhala kuti simuli ku sukulu? Ngati eeeh kutiko? Mumalipilidwa kapena ayi? | Do you do any work after school? Where do you work? Do you get paid or not? | Should remain the same | Should remain the same | Kodi mumalipidwa ndalama zingati mukamagwira ntchito kwa anthu ena kapena pakampani?  Mumagwirizana bwanji ndi omwe adakulembani ntchito (akupatsani ntchito) |
|  |  |  |  |  |  |
| A - ACTIVITIES | |  |  |  |  |
| What do you do for fun?What activities do you do during and after school? Are you active in sports? Do you exercise? | Kodi mumachita chiyani pa nthawi yanu yopuma?( monga kupita ku magulu a achinyamata, masewera olimbitsa thupi ngati mpira wa miyendo ndi wa manja) | What do you do on your free time? (attending Youth Clubs, any physical exercises e.g. football or netball)? | Should remain the same | Should remain the same | Should remain the same |
| Who are your friends?  Who do you do fun things with?  Who do you go to with problems?  What do you do on weekends? Evenings? | Anzinzanu ambiri muli nawo ndi ochokera kusukulu kapena madela ena? (ali ndi zaka zingati, ndi anyamata kapena atsikana, nanga amakonda chiyani?) | Where do most of your friends come from; school or other areas)? (What are their age ranges, are they boys or girls, what do they like?) | Should remain the same | Should remain the same | Kodi muli ndi anzanu ndipo amachokera kuti? |
|  | Kodi mumapita ku tchalitchi pafupi pafupi kapena kutenga nawo gawo pa zochitika za mu tchalitchi? | Do you regularly go to church or attend any religious activities? | Should remain the same | Should remain the same | What religion do you belong to? |
|  | Kodi pali nthawi zina zomwe mumakhala muli nokha nokha? Nanga mumaona kuti zili bwino bwino? | Is there any time you are all alone and do you find this okay to be all alone? | Should remain the same | Should remain the same | This question should be moved to Depression Section |
|  | Kodi pali nthawi yomwe mumakhala osungulumwa kapena kusalidwa pa magulu a anzanu pazochitika? Chimachitika ndi chani ndipo mumamva bwanji? | Do you ever feel lonely or discriminated against during activities or events? What happens and how do you feel? | Should remain the same | Should remain the same | Same here: To be moved to depression section |
| **D - DRUGS** |  |  |  |  |  |
| **D - DRUG USE AND ABUSE** | | | | |  |
|  | Achinyamata ena amagwiritsa ntchito mankhwala ozunguza bongo ngati fodya ndi mowa | Some young people abuse substances such as alcohol or cigarettes | Should remain the same | Should remain the same | Should remain the same |
| Do you drink coffee or tea? | Question was considered irrelevant | | | | |
| Do you smoke cigarettes? Have you ever smoked one? | Kodi mukudziwapo chiani za mankhwala ozunguza bongo? | What do you know about substance abuse? | Should remain the same | Should remain the same | Should remain the same |
| Have you ever tried alcohol? When? What kind and how often?  Do any of your friends drink or use drugs?  What drugs have you tried? Have you ever injected steroids or drugs  When?  How often do you use them?  How do you get money to pay for drugs?  Are drugs used or available in places where you hang out? | Kodi mudayamba mwagwiritsapo ntchito, kapena anzanu ena anayamba agwiritsapo ntchito mankhwala ozunguza bongowa? (monga marijuana, Kuber, Indian hemp,mowa) | Have you ever abused drugs or have your friends ever abused them? (e.g. Indian hemp, alcohol) | Question too long needs to be split and remove examples in brackets:  Kodi munayamba mwagwitsapo ntchito mankhawala ozunguza bongowa? The question should only end there. | Should remain the same | Nanga inu, munagwiritsapo ntchito mankhwala ozunguza bongo? |
|  | Ndi chifukwa chiyani anthu amatenga mankhwala ozunguza bongowa? | Why is it that people abuse drugs?  Have you ever abused drugs or have your friends ever abused them? (e.g. Indian hemp, alcohol) | Should remain the same | Should remain the same | N’chifukwa chiyani anthu amagwiritsa ntchito mankhwala ozunguza bongowa? |
|  |  |  |  |  |  |
| **S- SEXUAL AND REPRODUCTIVE HEALTH** | | | | | |
| **S-SEXUAL ACTIVITY/**  **IDENTITY** |  |  |  |  |  |
|  | **Achinyamata ena amapanga mchitidwe ogonana pa chibwenzi.** | **Some young people are involved in sexual relationships** |  |  |  |
| Do you feel you are ready for sex?  Have you chosen to remain abstinent?  How old were you when you first had sex? How old was your partner? Do you think you might be lesbian, gay or bisexual?  Do you think you need to have sex to find out if you are lesbian, gay or bisexual?  Do you want to become pregnant?  Have you ever been pregnant? Have you ever been tested for HIV?  Do you think it would be a good idea to be tested? | Munayamba mwawamverapo anzanu ena kuti amagonana ndi abwenzi awo? | Have you ever heard of anyone among your friends having a sexual relationship? | Should remain the same | Should remain the same | Should remain the same |
| Have you ever had sex? | Nanga inuyo, kodi munayamba mwakhalapo ndi chibwenzi chomwe mumagonana nacho ndipo ndinu omasuka nazo? | How about you? Have you ever been in a sexual relationship and are you comfortable with it? | Remove “nanga inuyo”, should read:  “Kodi munayamba mwakhalapo ndi chibwenzi chomwe mumagonana nacho ndipo ndinu omasuka?” | Kodi munayamba mwakhalapo ndi chibwenzi ndipo munayambapo mwakhalapo pamodzi ? | Should remain the same |
| How many sexual partners have you had? | Ngati munayamba mwakhalapo pa chibwenzi, mwagonapo ndi anthu angati? | If you have had any sexual relationship, how many sexual partners have you had? | Should remain the same | Should remain the same | Should remain the same |
|  | Pali wina anakuchitanipo nkhanza? Anakuchitani nkhanza motani ndipo munachitapo chiyani? | Is there any of them who sexually abused you? What happened and what did you do? | This question should be removed and placed on abuse section. | Add “Kodi” at the beginning. Kodi pali wina anakuchitanipo nkhanza ankuchitani nkhanza motani ndipo munachitapo chiyani? | Should remain the same |
|  | Munayamba mwafotokozerapo abwenzi anu kuti muli ndi kachilombo ka HIV? | Have you ever explained to your boyfriend (s)/girlfriend(s) about your HIV status? | Should remain the same | Kodi munayamba mwafotokozerapo abwenzi anu kuti muli ndi kachilombo ka HIV? | Should remain the same |
|  | Nanga abwenzi anu anakuwuzani ngati ali ndi kachilombo koyambitsa HIV kapena ayi? | Has your boy/girlfriend informed you about his/her HIV status or not? | Add at the beginning ngati muli ndi chibwenzi , the question should flow as follows:  Ngati muli ndi chibwenzi , abwenzi anu anakuwuzani ngati ali ndi kachilombo koyambitsa HIV kapena ayi? | Should remain the same | Should remain the same |
| Have you ever tried sex for money, drugs, clothes, or a place to stay? | Munayamba mwagonanapo ndi munthu ndi cholinga choti akupatseni ndalama kapena zinthu zimene mumasowa? | Have you ever exchanged sex with money or material things? | Should remain the same | Kodi munayamba mwagonanapo ndi munthu ndi cholinga choti akupatseni ndalama kapena zinthu zimene mumasowa? | Should remain the same |
| Have you ever had an infection as a result of having sex? | Ngati munayamba mwagonapo ndi abwenzi anu, munatulukapo ukazi kapena umuna omwe udakudabwitsani kuti mwina mwatenga matenda? | If you have had sex before with your boy/girlfriend, did you have a discharge that made you think you have contracted sexually transmitted infection (STI)? | Should remain the same | Should remain the same | Should remain the same |
| Do you use condoms or another form of contraception for STD and HIV prevention? | Kodi mukudziwapo chiyani pa nkhani yogonana modziteteza? | What do you know about “safer sex”? | Should remain the same | Should remain the same | Should remain the same |
|  | Munayamba mwamvapo za ma condom kapena njira zina zodzitetezera ku matenda opatsirana pogonana kapena mimba? | Have you ever heard of condoms or other forms of contraception to prevent against STI and/or pregnancy? | Should remain the same | Kodi munayamba mwamvapo za ma condom kapena njira zina zodzitetezera ku matenda opatsilana pogonana kapena mimba? | Should remain the same |
|  | Munayamba mwagwiritsako ntchito njira zomwe mwatchulazo? | Have you ever used either of the mentioned methods? | Should remain the same | Kodi munayamba mwagwiritsako ntchito njira zomwe mwatchulazo? | Should remain the same |
|  | Have you ever had sex unwillingly? | Kodi pali wina wake anakugwirani thupi lanu mosayenera/musakufuna, kapena kugonana nanu kumene musakufuna? | Has anyone ever touched you or forced to have sex with you without your consent? | Should remain the same | Should remain the same |
|  | Ngakhale mwakamba kuti simunakhalepo ndi chibwenzi, muli ndi chidwi ndi anyamata kapena atsikana. (Kapena simunaganizire za nkhaniyi, kapena munachita chisankho chosala kaye). (Kwa amene sanayambe zibwenzi). | Although you have said that you have never had any relationship, do you have interest in boys or girls? (Perhaps you’re not sure? Or have you chosen to remain abstinent)? (For those not involved in sexual activities) | Should remain the same | Ngakhale mwakamba kuti simunakhalepo ndi bwenzi muli ndi chidwi ndi anyamata kapena atsikana.(Kapena simunaganizire za nkhaniyi, kapena munachita chisankho chodziletsa kaye).(Kwa amene sanayambe zibwenzi).. | Should remain the same |
|  |  |  |  |  |  |
| **S - SUICIDE/DEPRESSION** | | |  |  |  |
| How do you feel today, on a scale of 0 - 10 (0 = very sad, 10 = very happy)?  What made you feel that way?  Does thinking you may be lesbian, gay, or bisexual make you feel that way?  Did you ever think about hurting yourself or that life isn't worth living, or hope that when you go to sleep you won't wake up? |  |  |  |  |  |
|  | Achinyamata ambiri amakhala ogwetsedwa mphwayi, okhumudwa ndiponso osasangala | Many young people feel discouraged, sad and low/not happy |  |  |  |
|  |  |  |  |  |  |
| Have you ever felt less than a 5? How long did that feeling last? | Munayamba mwakhalako mu mmaganizo oterawa. | Have you ever been in such situations? | Should remain the same | Should remain the same. But add “kodi” at the beginning:  Kodi Munayamba mwakhalako mu mmaganizo oterawa? | Should remain the same |
|  | Zimachitika pafupi pafupi bwanji, ndipo kwa nthawi yayitali bwanji? | How often does this happen and for how long? | Should remain the same | Kodi kukhumudwako kumachitika pafupi pafupi bwanji, ndipo kwa nthawi yayitali bwanji? | Should remain the same |
|  | Kodi mumakhala ndi maganizo obwelera mbuyo pafupi pafupi? N’chifukwa chani? | Do you ever have negative thoughts most often and why? | Should remain the same | Should remain the same | Should remain the same |
|  | Kodi pali nthawi ina yomwe simumafuna kukhala ndi anzanu, kapena achibale anu kwa nthawi yayitali? N’chifukwa chiyani zili choncho? | Is there any time that you don’t feel like being with your friends, or relatives for a long time? Why is it so? | Should remain the same | Should remain the same | Should remain the same |
|  | Kodi muli ndi khalidwe lililonse limene limakulepheretsani kukhala pakati pa achibale kapena anzanu kusukulu, ngakhale m’dera lanu (mwachitsanzo, kukangana kangana)? | Do you have any type of behaviour that makes it difficult for you to be among your relatives or friends at school, even in your community (for example, quarrelling)? | Should remain the same | Should remain the same | Should remain the same |
|  | Kodi mumadya bwanji, mumagona bwanji komanso mumalimbikitsidwa bwanji? | How are your eating habits, sleeping patterns and being motivated? | Should remain the same | Should remain the same | Should remain the same |
|  | Anthu ena amakhala okhumudwa kwambiri kufikira pofuna kuwononga moyo wawo | Some people become so depressed that they want to ruin their lives | Should remain the same | Should remain the same | Should remain the same |
|  | Kodi munayamba mwadzipwetekapo nokha kapena kuganiza zopweteka munthu wina (mwachitsanzo kudzicheka, kudziwotcha, kapena kudzikanda) | Have you ever hurt yourself or thought of hurting someone (for example cutting, burning, scratching) | Should remain the same | Should remain the same | Should remain the same |
|  | Mwa achinyamata, Kodi mukudziwa wina amene anadzipha chifukwa chovutika maganizo? | Among young people, do you know someone who committed suicide because of depression? | Should remain the same | Should remain the same | Kodi mukudziwa wina mwa achinyamata, amene anadzipha chifukwa chovutika mmaganizo? |
