# Supplementary material for: Cultural adaptation of a psychosocial screening tool for adolescents living with HIV/AIDS attending antiretroviral therapy program in Malawi
Source: PLoS One. 2025 Nov 17;20(11):e0318738. doi: 10.1371/journal.pone.0318738 (PMC12622793; doi:10.1371/journal.pone.0318738)
Supplement: S1 File — English Focus Group Discussion Guide. S2 Text. Chichewa Focus Group Discussion Guide. S3 Text. Original HEADSS tool. S4 Text. Participants HEADSS adaptation notes_v1. S5 Text. HEADSS adaptation v1. S6 Text. Participants HEADSS adaptation notes_ v2. S7 Text. HEADSS adaptation v2. S8 Text. HEADSS adaptation v3. S9 Text. HEADSS adaptation _v4_Final Version. (ZIP) [file pone.0318738.s001.zip › Supporting Information/Supplementary File 7.docx]

**Supplementary File 7 - HEADSS adaptation v2**

| DOMAINS | INTERVIEWS QUESTIONS |
| --- | --- |
| H – HOME AND ENVIRONMENT | |
| 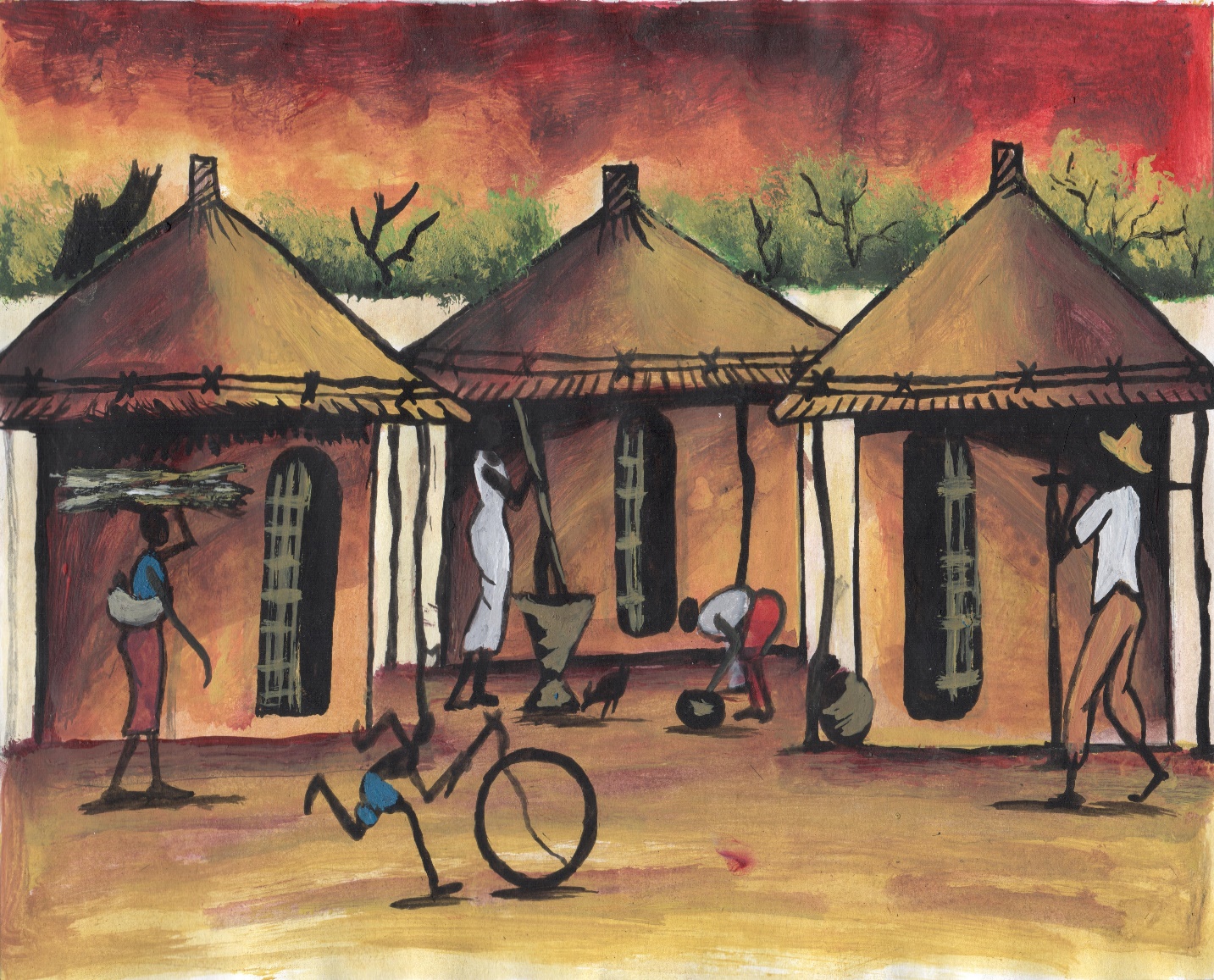 | **RELATIONSHIPS AT HOME**   - May I know your name and where you stay? - *Mungandiwuze dzina lanu ndi komwe mumakhala?* - Whom do you stay with? (Biological parents, an auntie, uncle or grandparents and other relatives/siblings?) - *Mumakhala ndi ndani? (Makolo okubelekani, a zakhali, amalume kapena agogo* - *ndi abale anu ena?)* - Tell me more about these relatives of yours? - *Mungandiwuze zambiri za achibale anuwa?* - How do you get along with your relatives? - *Mumakhalitsana motani ndi achibale anuwa?* - Do you live with them happily, in peace and feeling part of your family? - *Kodi mumakhala mosangalala, mwa mtendere ndi movomerezedwa m’banja mwanu?* - Have you ever run away from your home? If yes, why did you do so and where did you go to stay? - *Munayamba mwathawapo kunyumba komwe mumakhalako? Ngati munathawako, ndi chifukwa chiyani munathawa ndipo munapita kukakhala kuti?* - Is there anybody in your family, who is on ART? - *Kodi alipo wina akumwanso mankhwala mbanja mwanu?* |
| 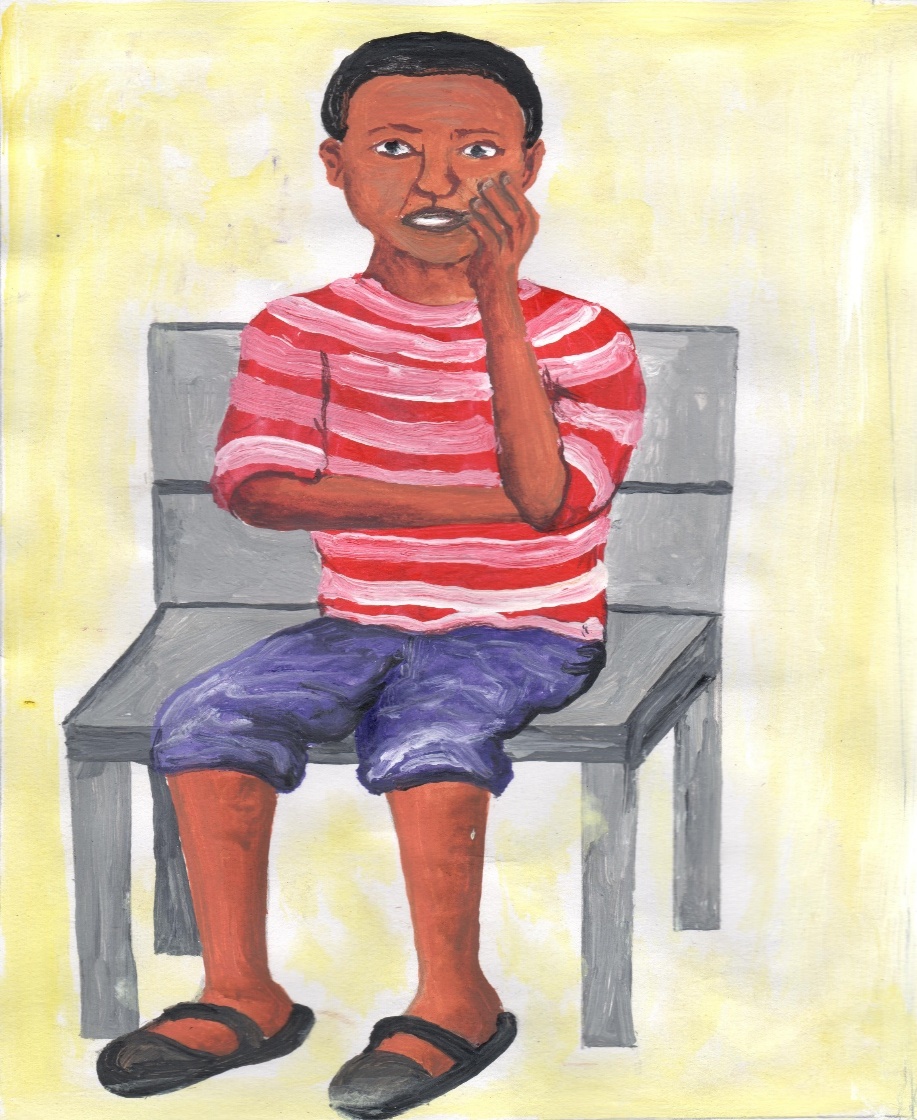 | **STRESS DUE TO MISTREATMENT**  **Sometimes people get stressed up by what is happening around them, have you ever been stressed up?**  ***Zimachitika kuti nthawi zina munthu umatha kupanikizika mmaganizo, kukhala odandaula ndi zochitika. Munayamba mwakhalapo odandaula?***   - Can you explain to me the problems that make you get stressed at home? - *Mungandifotokozereko mavuto amene amakusowetsani mtendere pakhomopo?* - Do you worry a lot about things and find the worry just won’t go away? - *Kodi mumakhala odandaula mu zinthu zambiri ndikuona kuti nkhawa zanu sizikuchoka? ndi kudandaulanso kwa nthawi yayitali?* - Who do you talk to at home when you are stressed? - *Kodi mumamukhuthukira ndani mukakhala ndi nkhawa kapena mukapanikizika mmaganizo?* |
| 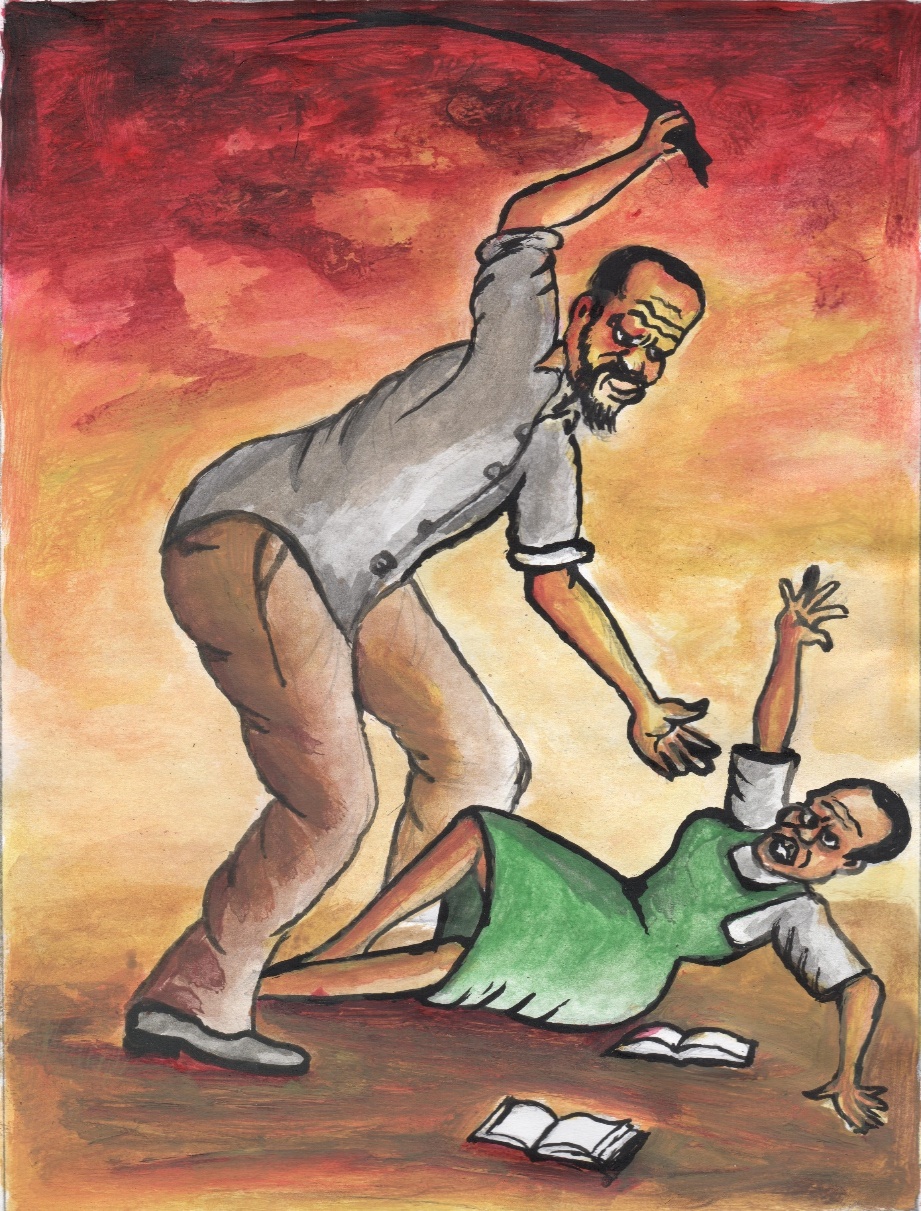 | **PHYSICAL ABUSE**   - In the past year, have you ever been physically abused or been beaten by somebody? - *Mchaka chathachi, mwapangidwako nkhanza kapena kumenyedwa ndi wina aliyense?* - If yes, can you explain how the abuse happened? - *Mungafotokoze mmene nkhazazo zinachitikira?* |
| 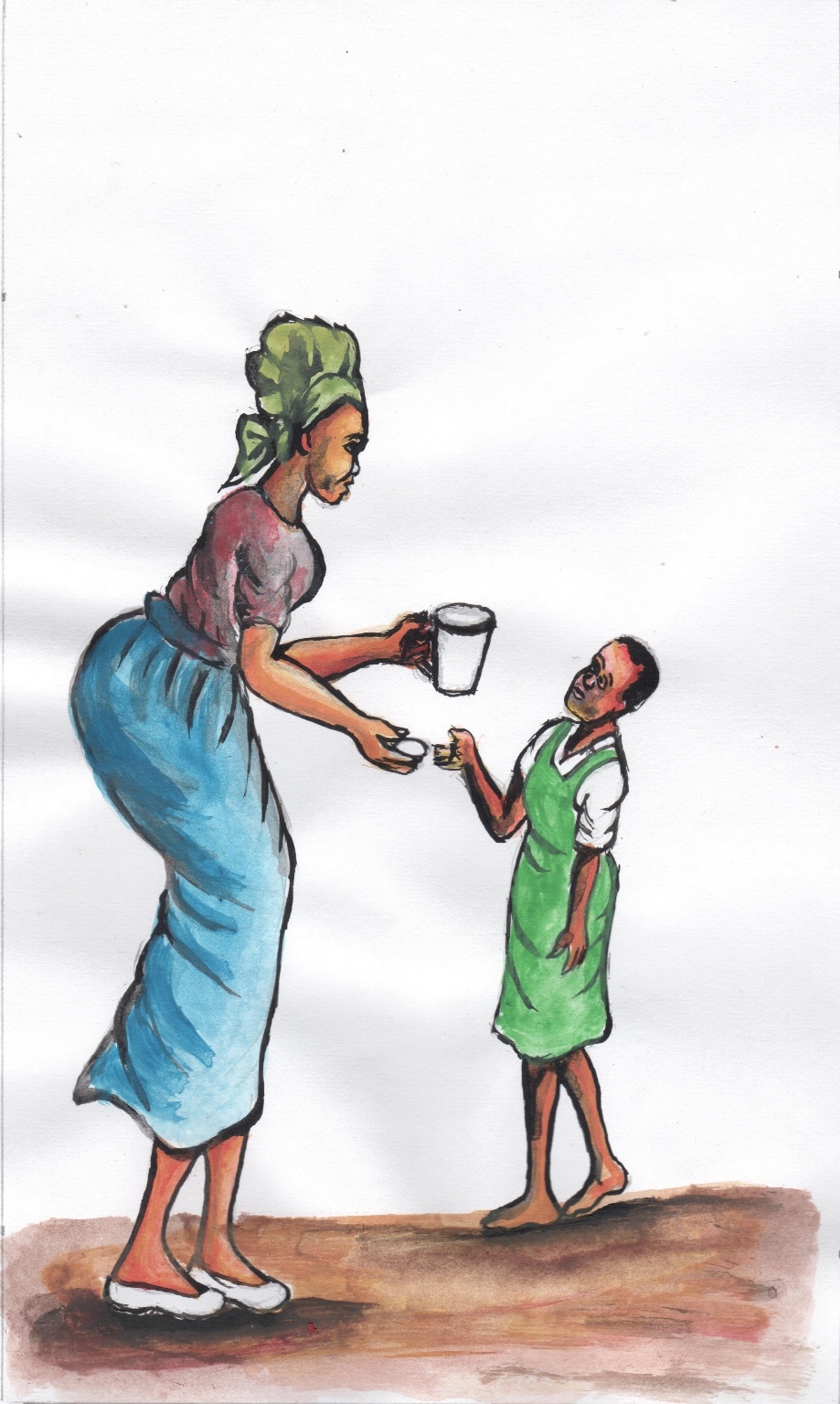 | **ADHERENCE TO ANTIRETROVIRAL THERAPY**   - Apart from your friends in Teen Club or your family, have you disclosed your HIV status to other people? - *Kupatula anzanu kuno ku Teen Club ndi aku banja kwanu, munawuzapo anthu ena za kuti muli ndi kachilombo ka HIV?* - Who supports/encourages you to take your medications/ARVs? - *Amene amakulimbikitsani ndi ndani zakutsata ndondomeko ya kamwedwe ka mankhwala anu?* - How often do you miss taking your medications/ARVs? - *Mumadumphitsa kumwa mankhwala anu mowirikiza bwanji?* - How do you manage taking your medications/ARVs at school? (Only for those in boarding schools) - *Nanga mukakhala ku sukulu, mamwedwe anu a makhwala amakhala otani? (kwa okhawo ali ku sukulu yogonera konko)* |
| 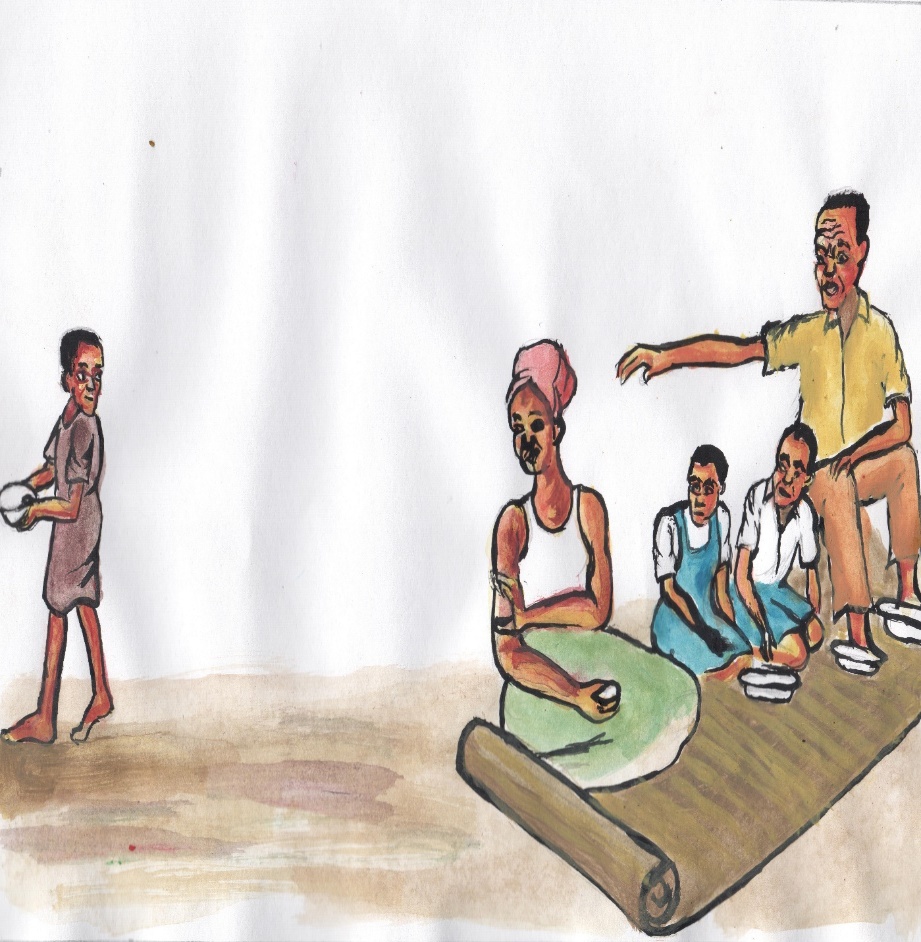 | **STIGMA AND DISCRIMINATION**   - Do you feel discriminated, being bullied and stigmatized in any way because you are HIV positive?(in the community, home or at school) - *Kodi mumadzimva kuti mukusankhidwa, kutonzedwa kapena kusalidwa munjila ina iliyonse chifukwa muli ndi kachilombo ka HIV (kudela kwanu, kunyumba kapena kusukulu)* - What difficulties/problems do you face because of what other people say about you at school or at home? - *Kodi mumakumana ndi mavuto anji, kamba ka zoyankhula za anthu ku sukulu kapena kunyumba?* - Are there any changes in your body that have resulted from taking the ARVs that you are worried about? (Lipodystrophy or gynecomastia)? - *Pali zosintha za m’thupi mwanu zokhudzana ndi kumwa mankhwala zomwe zikukudandaulitsani? (ngati kukula mabele kwa achinyamata kapena kusintha nkhope)* |
| 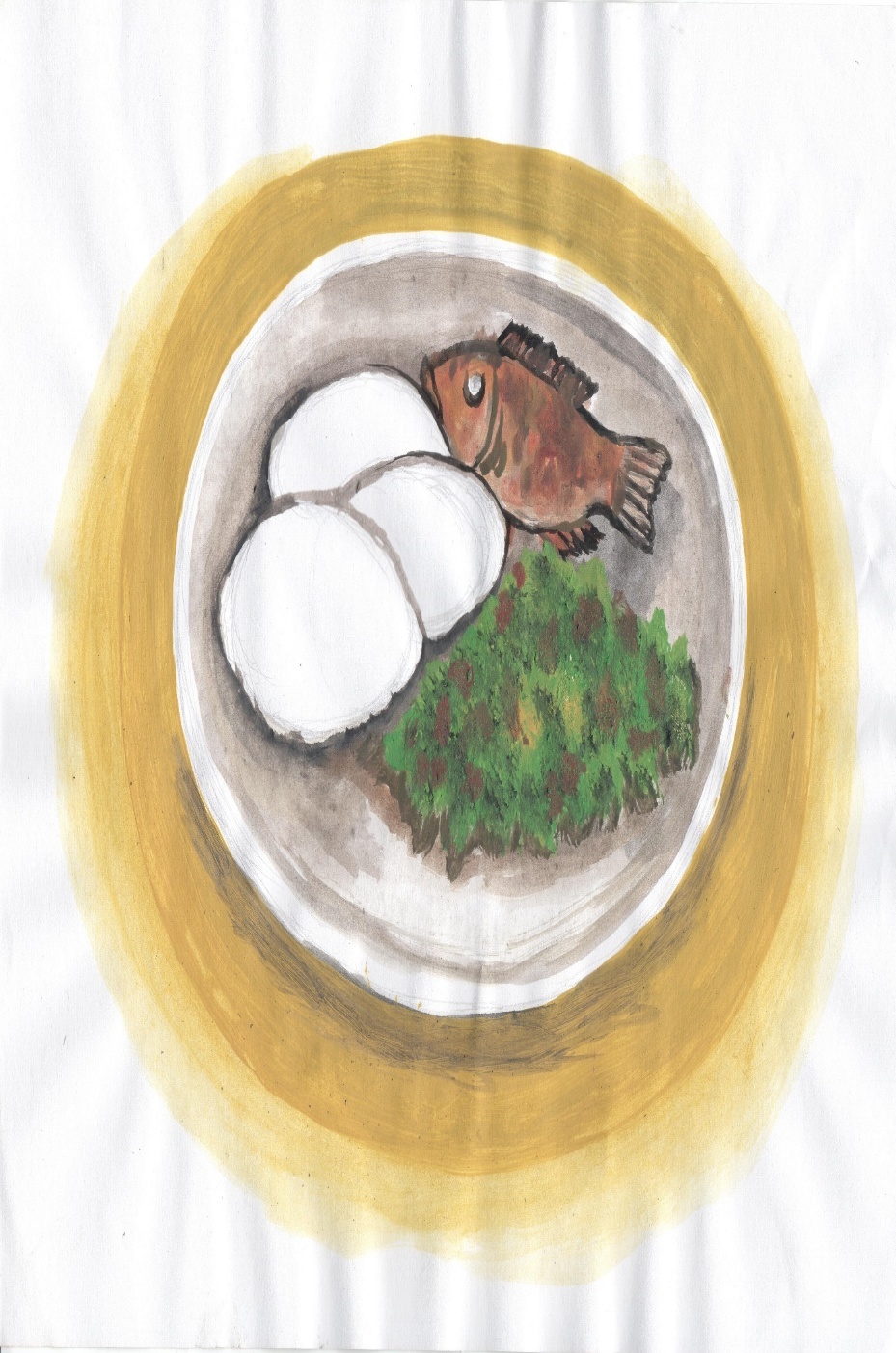 | **FOOD SECURITY**   - May you explain to me, what type of foods do you normally eat in a day; breakfast, lunch, and supper? - *Mungandifotokozereko zakudya zomwe mumanya patsiku; m’mawa , masana ndi madzulo.* - Do you normally eat what you want at home? - *Kodi mumadya zakudya zomwe inu mukufuna?* - How do your parents find the food in the home? - *Kodi makolo anu zakudya zimenezi amapeza bwanji?* |
| E – EDUCATION AND EMPLOYMENT | |
| 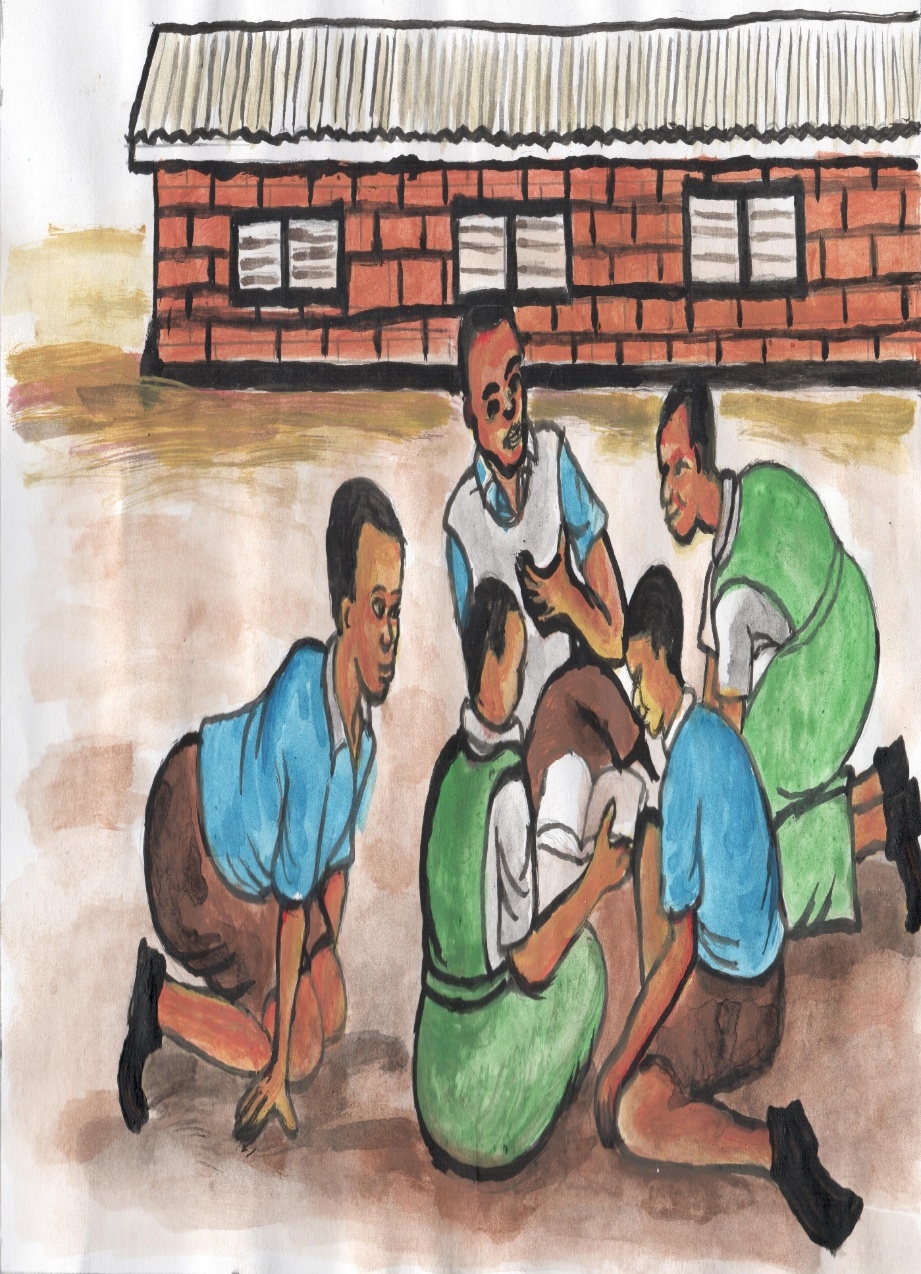 | - Are you in school? - *Kodi muli pa sukulu?* - Are you in primary school, secondary school or tertiary institution? Are you in boarding or not? - *Kodi muli ku pulayimale, sekondale kapena sukulu ya ukachenjede/yoyendera kapena yogonera pompo?* - Who pays for your school fees, uniform, and school materials? - *Kodi amakulipilirani fizi, kukugulirani uniform ndi zofunikira ku sukulu ndani?* - Do you face any problems at school?? (Inquire about “bullying”). - *Kodi mmakumana ndi zotani ku sukulu? (kuzunzidwapo/kumenyedwapo*) - Have you ever repeated a class, suspended or expelled from school? Why did this happen? - *Kodi munabwerezapo kalasi, kuyimitsidwa kapena kuchotsedwa? Chifukwa chiyani*? - Have you been absent from school this month, three months ago or the previous term? (what was the reason) - *Kodi mwajombako ku sukulu mwezi uno, miyezi itatu yapitayi kapena teremu yathayi? (pali zifukwa zanji?)* - Have you ever considered dropping out of school? - *Munayamba mwalingalirapo zosiya sukulu?* - Do you have anybody at school whom you freely discuss important issues with? (Who is he/she)? - *Pali wina wake amene mumakhala omasuka naye ku sukulu, yemwe mumakamba naye zinthu zofunikira? (ndipo ndi ndani?)* - What do you want to do after school, what future plans do you have regarding your career? - *Kodi mumafuna kudzapanga chiyani mukamaliza sukulu?* - *Muli ndi malingaliro anji atsogolo lanu pa ntchito yomwe mumafuna kudzagwira?*   **EMPLOYMENT**   - Do you do any work after school? Where do you work? Do you get paid or not? - Kodi mumagwira ntchito ina iliyonse mukakhala kuti simuli ku sukulu? Ngati eeeh kutiko? Mumalipilidwa kapena ayi? - How do you get along with the ones who employed you? - *Kodi mumakhalitsana nawo bwanji amene anakulembani ntchitowo?* |
|  |  |
| A - ACTIVITIES | |
| 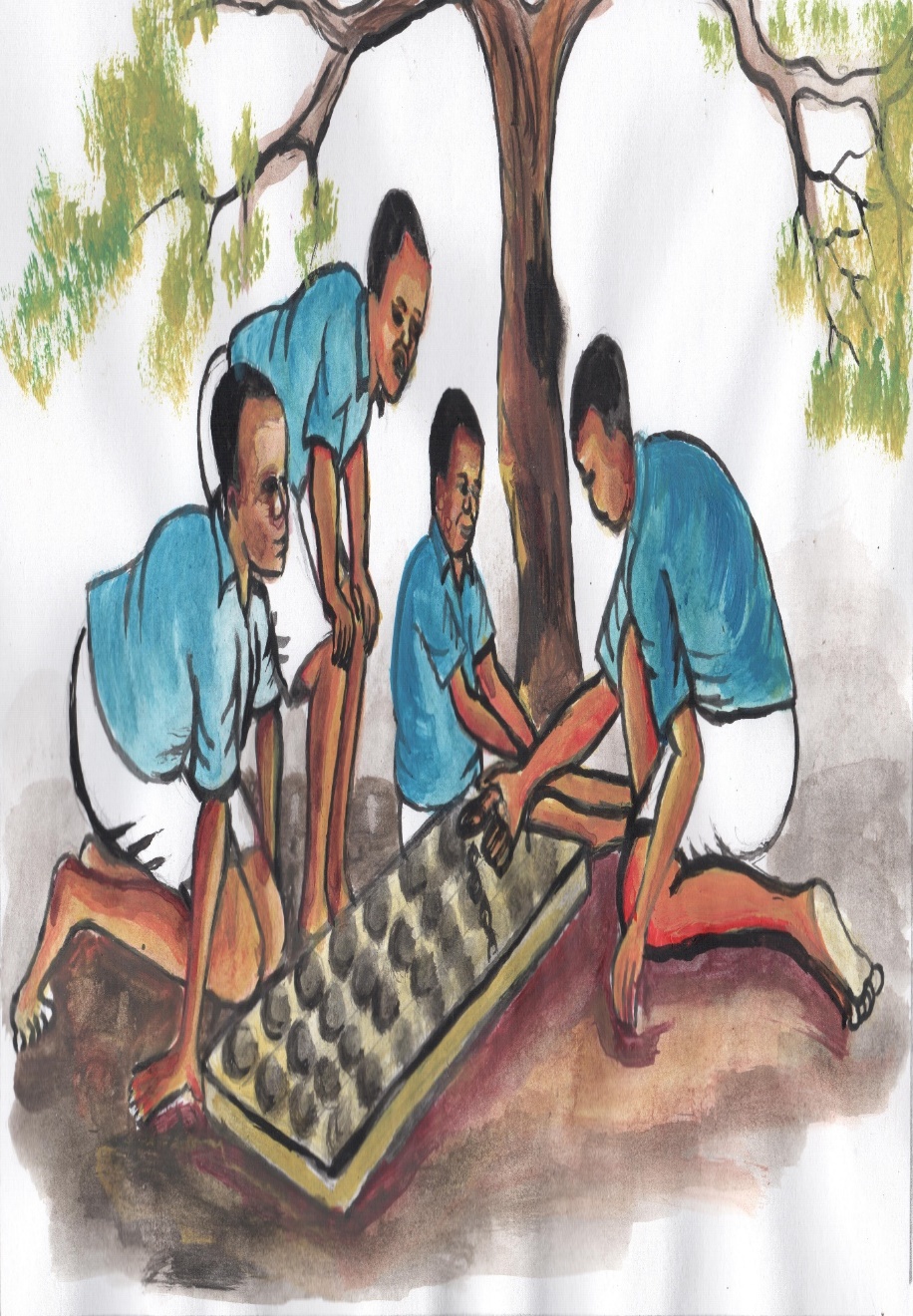 | - What do you do on your free time? (attending Youth Clubs, any physical exercises e.g. football or netball)? - *Kodi Mumachita chiyani pa nthawi yanu yopuma?( monga kupita ku magulu a achinyamata, masewero olimbitsa thupi ngati mpira wa miyendo ndi wa manja)* - Where do most of your friends come from; school or other areas)? (What are their age ranges, are they boys or girls, what do they like?) - *Anzinzanu ambiri muli nawo ndi ochokera kusukulu kapena madela ena? (ali ndi zaka zingati, ndi anyamata kapena atsikana, nanga amakonda chiyani?)* - Do you regularly go to church or attend any religious activities? - *Kodi mumapita ku tchalitchi pafupi pafupi kapena kutenga nawo gawo pa zochitika za mu tchalitchi?* - Is there any time you are all alone? Do you find this okay to be all alone? - *Kodi pali nthawi zina zomwe mumakhala muli nokha nokha? Nanga mumaona kuti zili bwino bwino? TO BE MOVED TO DEPRESSION* - Do you ever feel lonely or discriminated against during activities or events? What happens and how do you feel? - *Kodi pali nthawi yomwe mumakhala osungulumwa kapena kusalidwa pa magulu a anzanu pazochitika? Chimachitika ndi chani ndipo mumamva bwanji?* |
| D – DRUGS (SUBSTANCE ABUSE) | |
| 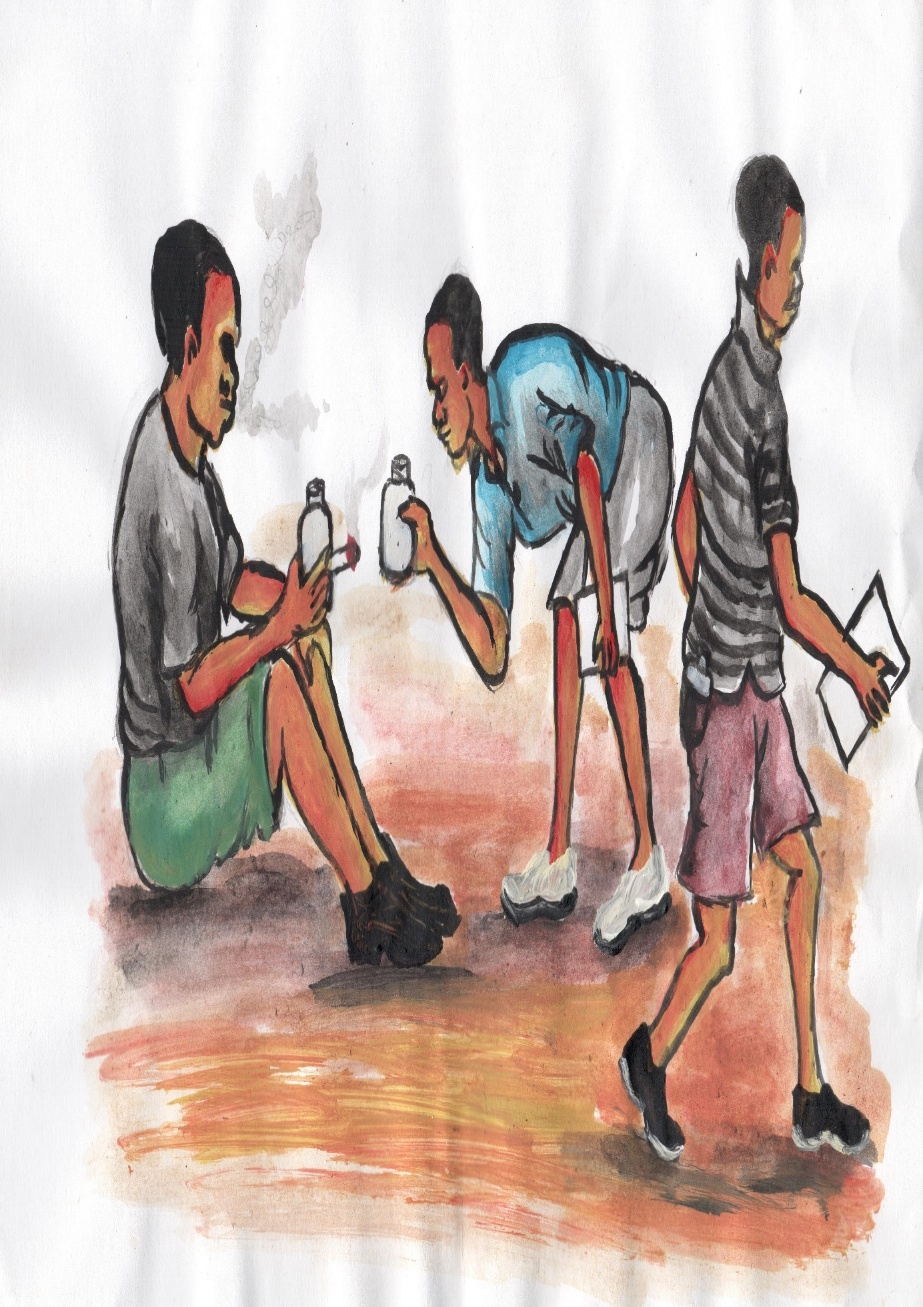 | **Some young people abuse substances such as alcohol or cigarettes**  ***Achinyamata ena amagwiritsa ntchito mankhwala ozunguza bongo ngati fodya ndi mowa***   - What do you know about substance abuse? - *Kodi mukudziwapo chiani za mankhwala ozunguza bongo?* - Have you ever abused drugs or have your friends ever abused them? (e.g. Indian hemp, alcohol) - *Kodi mudayamba mwagwiritsapo ntchito, kapena anzanu ena anayamba agwiritsapo ntchito mankhwala ozunguza bongowa? (monga marijuana, Kuber, Indian hemp,mowa)* - Why is it that people abuse drugs? - *Ndi chifukwa chiyani anthu amatenga mankhwala ozunguza bongowa?* |
| S-SEXUAL AND REPRODUCTIVE HEALTH | |
| 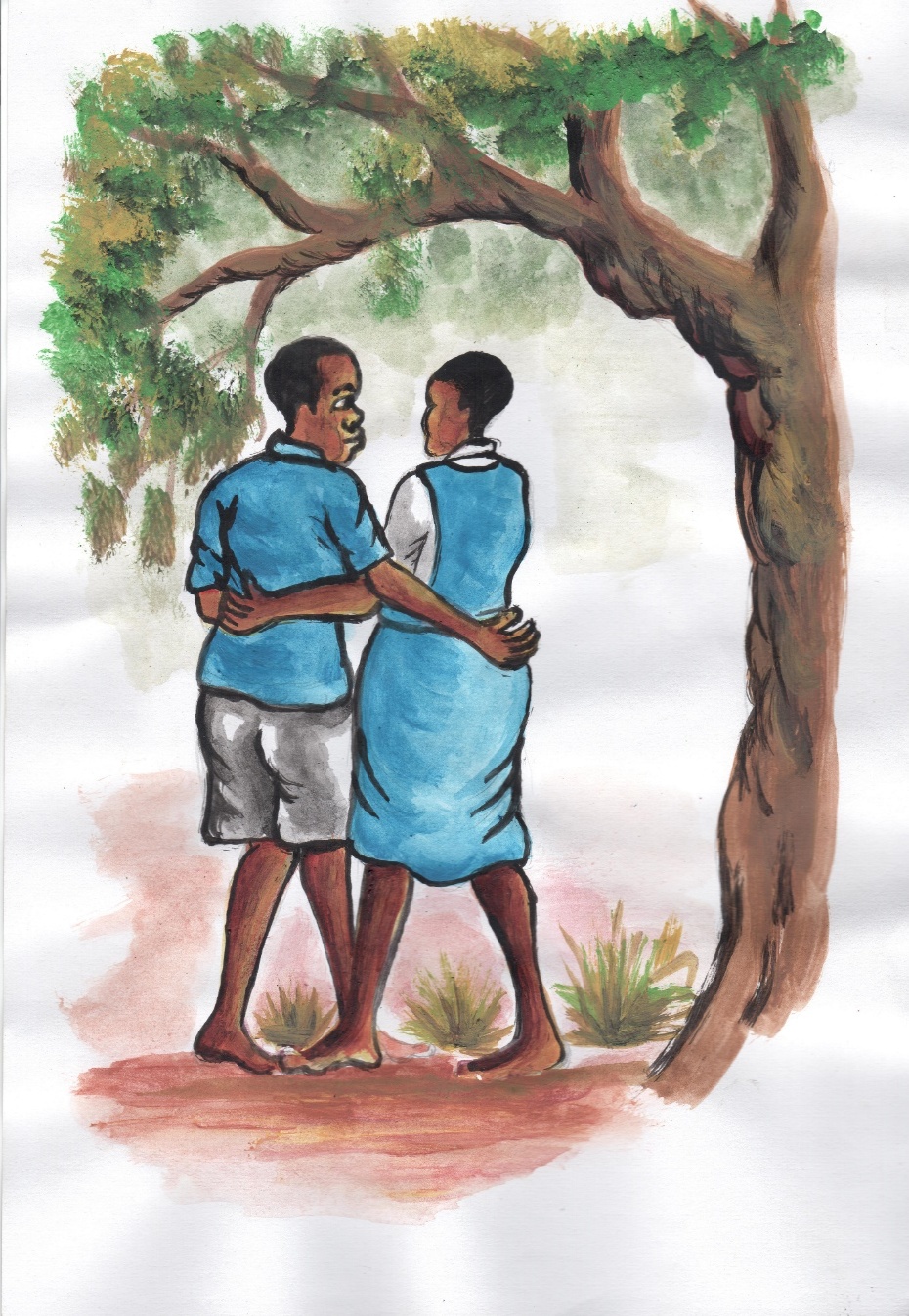 | **Some young people are involved in sexual relationships**  ***Achinyamata ena amapanga mchitidwe ogonana pa chibwenzi.***   - Have you ever heard of anyone among your friends having a sexual relationship? - *Munayamba mwawamverapo anzanu ena kuti amagonana ndi abwenzi awo?* - How about you? Have you ever been in a sexual relationship and are you comfortable with it? - *Nanga inuyo,* kodi *munayamba mwakhalapo ndi chibwenzi chomwe mumagonana nacho ndipo ndinu omasuka nazo?* - If you have had any sexual relationship, how many sexual partners have you had? - *Ngati munayamba mwakhalapo pa chibwenzi, mwagonapo ndi anthu angati?* - Is there anyone who sexually abused you? What happened and what did you do? - *Pali wina anakuchitanipo nkhanza? Anakuchitani nkhanza motani ndipo munachitapo chiyani? TO BE MOVED TO PHYSICAL ABUSE* - Have you ever explained to your boyfriend (s)/girlfriend(s) about your HIV status? - *Munayamba mwafotokozerapo abwenzi anu kuti muli ndi kachilombo ka HIV?* - Has your boy/girlfriend informed you about his/her HIV status or not? - *Nanga abwenzi anu anakuwuzani ngati ali ndi kachilombo koyambitsa HIV kapena ayi?* - Have you ever exchanged sex with money or material things? - *Munayamba mwagonanapo ndi munthu ndi cholinga choti akupatseni ndalama kapena zinthu zimene mumasowa?* - If you have had sex before with your boy/girlfriend, did you have a discharge that made you think you have contracted sexually transmitted infection (STI)? - *Ngati munayamba mwagonapo ndi abwenzi anu, munatulukapo ukazi kapena umuna omwe udakudabwitsani kuti mwina mwatenga matenda?* - What do you know about “safer sex”? - *Kodi mukudziwapo chiyani pa nkhani yogonana modziteteza?* - Have you ever heard of condoms or other forms of contraception to prevent against STI and/or pregnancy? - *Munayamba mwamvapo za ma condom kapena njira zina zodzitetezera ku matenda opatsirana pogonana kapena mimba?* - Have you ever used either of the mentioned methods? - *Munayamba mwagwiritsako ntchito njira zomwe mwatchulazo?* - Has anyone ever touched you or forced to have sex with you without your consent? - *Kodi pali wina wake anakugwirani thupi lanu mosayenera/musakufuna, kapena kugonana nanu kumene musakufuna?TO BE MOVED TO PHYSICAL ABUSE* - Although you have said that you have never had any relationship, do you have interest in boys or girls? (Perhaps you’re not sure? Or have you chosen to remain abstinent)? (***For those not involved in sexual activities)*** - *Ngakhale mwakamba kuti simunakhalepo ndi chibwenzi, muli ndi chidwi ndi anyamata kapena atsikana. (Kapena simunaganizire za nkhaniyi, kapena munachita chisankho chosala kaye).* ***(Kwa amene sanayambe zibwenzi).*** |
| **S - SUICIDE/DEPRESSION** | |
| 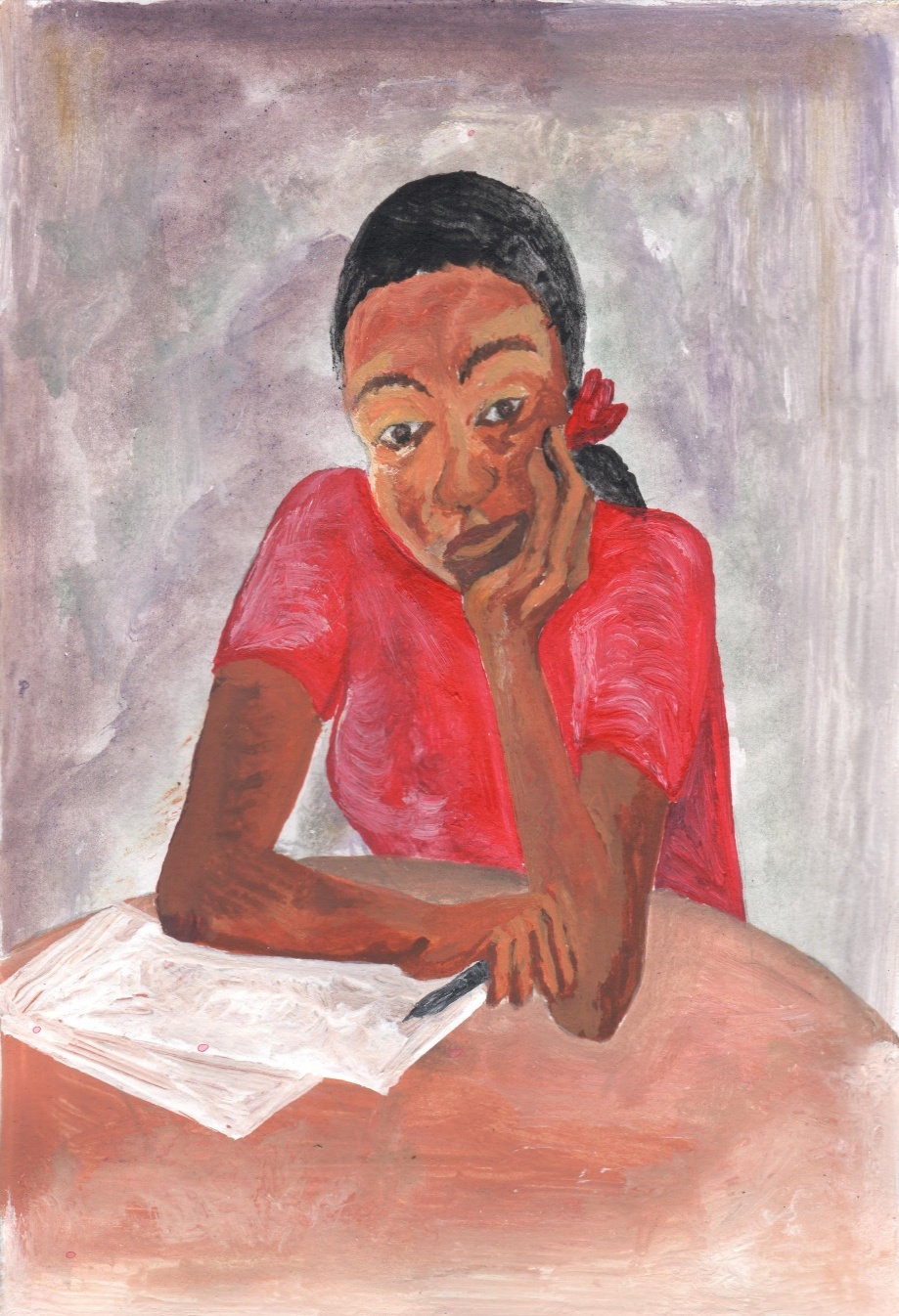 | **DEPRESSION**  **Many young people feel discouraged, sad and low/not happy.**  ***Achinyamata ambiri amakhala opanda chilimbikitso, okhumudwa ndi osasangalala.***   - Have you ever been in such situations? - *Munayamba mwakhalako mu mmaganizo oterawa*. - How often does this happen and for how long? - *Zimachitika pafupi pafupi bwanji, ndipo kwa nthawi yayitali bwanji?* - Do you ever have negative thoughts most often and why? - *Kodi mumakhala ndi maganizo obwelera mbuyo pafupi pafupi? N’chifukwa chani?* - Is there any time that you don’t feel like being with your friends, or relatives for a long time? Why is it so? - *Kodi pali nthawi ina yomwe simumafuna kukhala ndi anzanu, kapena achibale anu kwa nthawi yayitali? N’chifukwa chiyani zili choncho?* - Do you have any type of behaviour that makes it difficult for you to be among your relatives or friends at school, even in your community (for example, quarrelling)? - *Kodi muli ndi khalidwe lililonse limene limakulepheretsani kukhala pakati pa achibale kapena anzanu kusukulu, ngakhale m’dera lanu (mwachitsanzo, kukangana kangana)?* - How are your eating habits, sleeping patterns and being motivated? - *Kodi mumadya bwanji, mumagona bwanji komanso mumalimbikitsidwa bwanji?* |
| 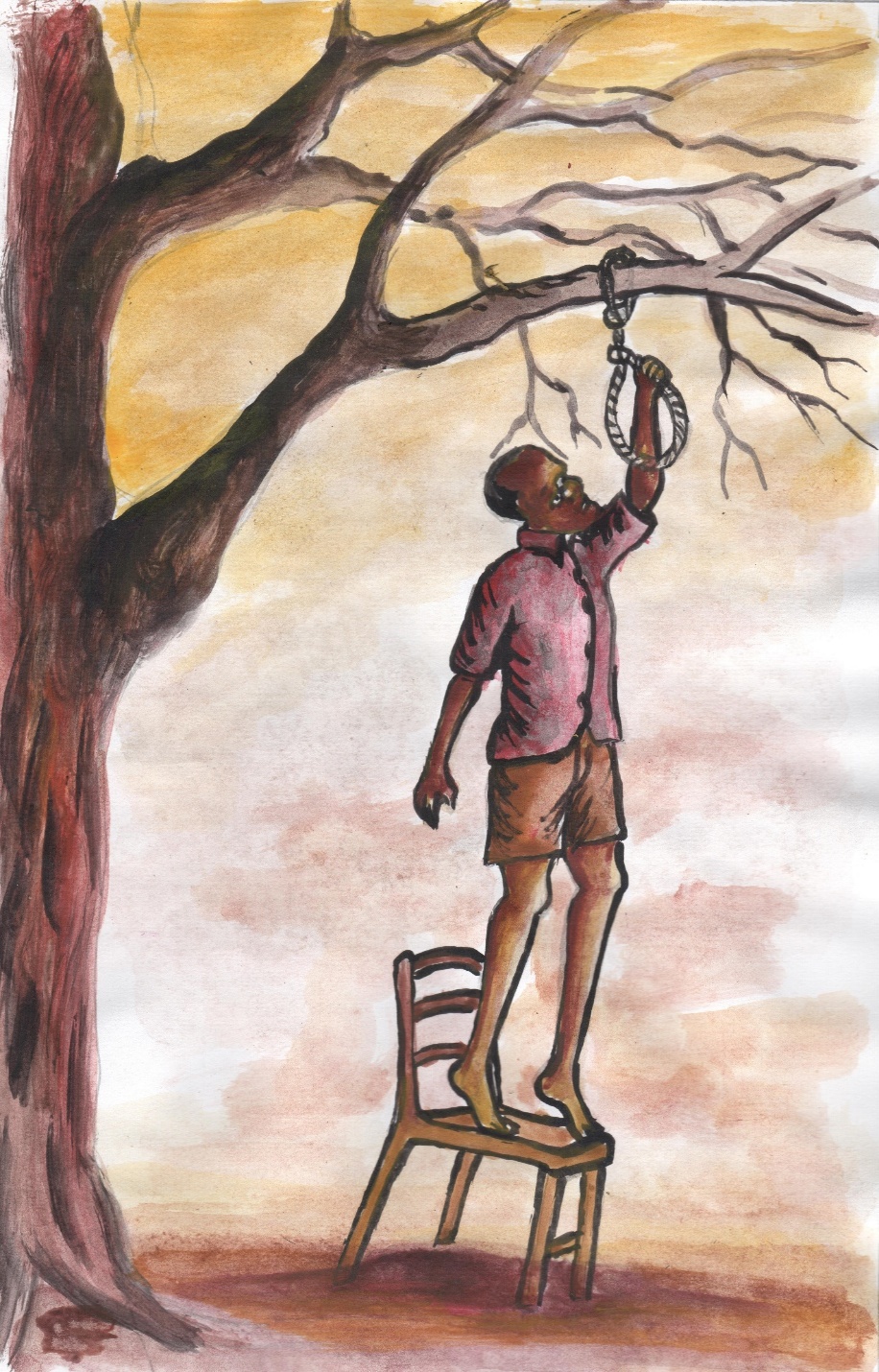 | **SUICIDE ISSUES**  **Some people become so depressed that they want to ruin their lives**  ***Anthu ena amakhala okhumudwa kwambiri kufikira pofuna kuwononga moyo wawo***.   - Have you ever hurt yourself or thought of hurting someone (for example cutting, burning, scratching) - *Kodi munayamba mwadzipwetekapo nokha kapena kuganiza zopweteka munthu wina (mwachitsanzo kudzicheka, kudziwotcha, kapena kudzikanda)* - Among young people, Do you know someone who committed suicide because of depression? - *Mwa achinyamata, Kodi mukudziwa wina amene anadzipha chifukwa chovutika maganizo?* |
