# Supplementary material for: Cultural adaptation of a psychosocial screening tool for adolescents living with HIV/AIDS attending antiretroviral therapy program in Malawi
Source: PLoS One. 2025 Nov 17;20(11):e0318738. doi: 10.1371/journal.pone.0318738 (PMC12622793; doi:10.1371/journal.pone.0318738)
Supplement: S1 File — English Focus Group Discussion Guide. S2 Text. Chichewa Focus Group Discussion Guide. S3 Text. Original HEADSS tool. S4 Text. Participants HEADSS adaptation notes_v1. S5 Text. HEADSS adaptation v1. S6 Text. Participants HEADSS adaptation notes_ v2. S7 Text. HEADSS adaptation v2. S8 Text. HEADSS adaptation v3. S9 Text. HEADSS adaptation _v4_Final Version. (ZIP) [file pone.0318738.s001.zip › Supporting Information/Supplementary File 8.docx]

**Supplementary File 8 - HEADSS Adaptation _v3**

|  | INTERVIEWS QUESTIONS |
| --- | --- |
| H – HOME AND ENVIRONMENT | |
| 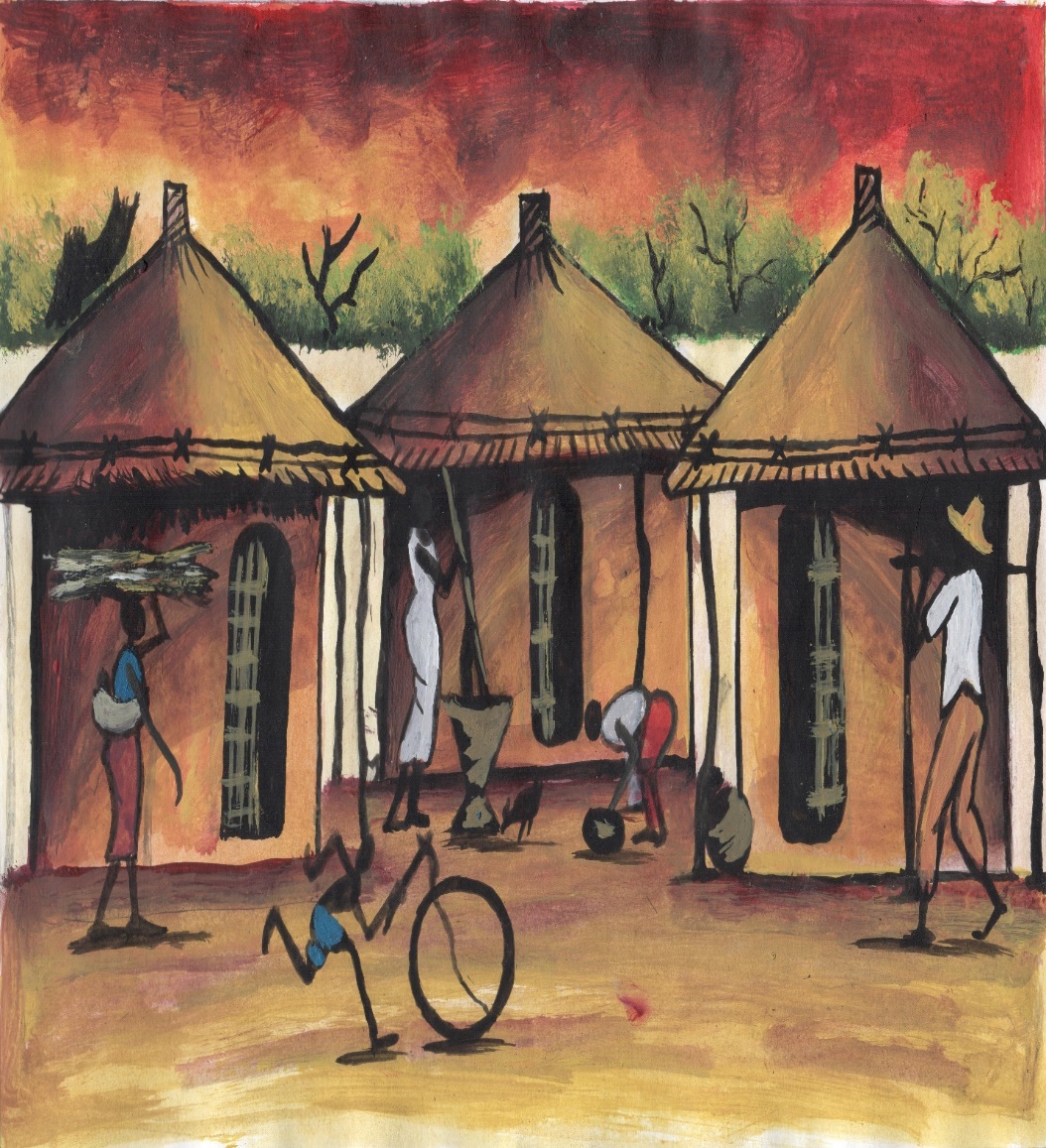 | **RELATIONSHIPS AT HOME**   - What’s your name and where do you live?   Mungandiwuze dzina lanu ndi komwe mumakhala?   - Who do you live with? (Biological parents, Auntie, Uncle, Grandma or grandparents or siblings)?   Mumakhala ndi ndani? (Makolo okubelekani, a zakhali, amalume kapena agogo ndi abale anu ena?)   - Tell me about your parents/ Auntie, Uncle, Grandma or grandparents/siblings   Mungandiwuze zambiri za achibale anuwa?   - How do you get along with your relatives?   Mumakhalitsana motani ndi achibale anuwa?   - Do you feel happy, safe and belonging to your family?   Kodi mumakhala mosangalala, mwa mtendere ndi movomerezedwa mbanja mwanu?   - Have you ever run away from home? If yes, Why and where do you go?   Munayamba mwathawapo kunyumba komwe mumakhalako? Ngati munathawako, ndi chifukwa chiyani munathawa ndipo munapita kukakhala kuti?   - Is anyone else on ART in your family?   Kodi alipo wina akumwanso mankhwala mbanja mwanu ? |
| 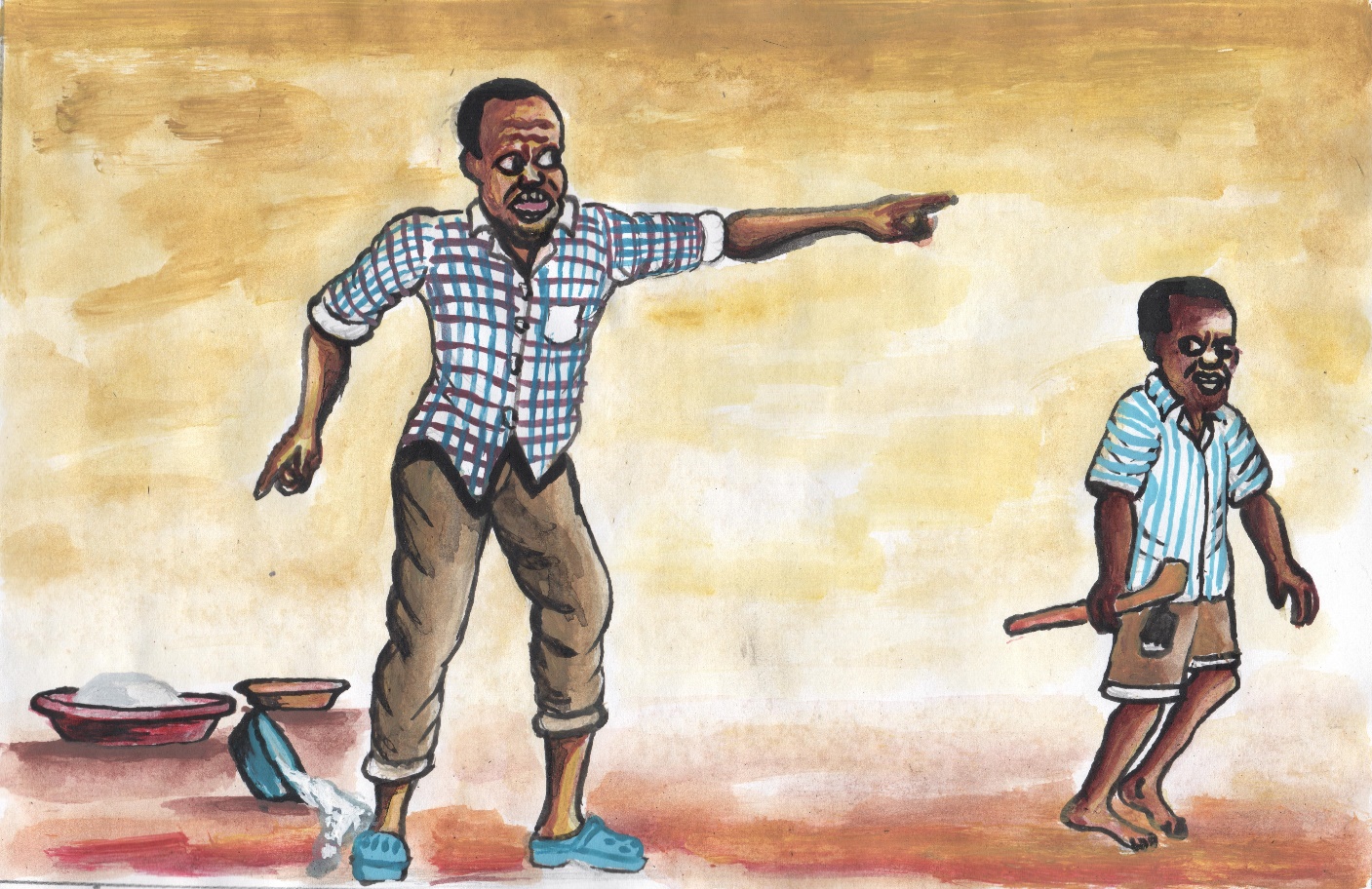 | **STRESS DUE TO MISTREATMENT**  It’s normal to feel stressed or worried in certain situations. Do you feel nervous or stressed?  Zimachitika kuti nthawi zina munthu umatha kupanikizika mmaganizo, kukhala odandaula ndi zochitika. Munayamba mwakhalapo odandaula?   - Explain to me any problems that worry you at home?   Mungandifotokozereko mavuto amene amakusowetsani mtendere pakhomopo?   - Do you worry a lot about things and find the worry just won’t go away?   Kodi mumakhala odandaula kopyola ndi kudandaulanso kwa nthawi yayitali?   - Who do you talk to at home when you are stressed?   Kodi mumamukhuthukira ndani mukakhala ndi nkhawa kapena mukapanikizika mmaganizo? |
| 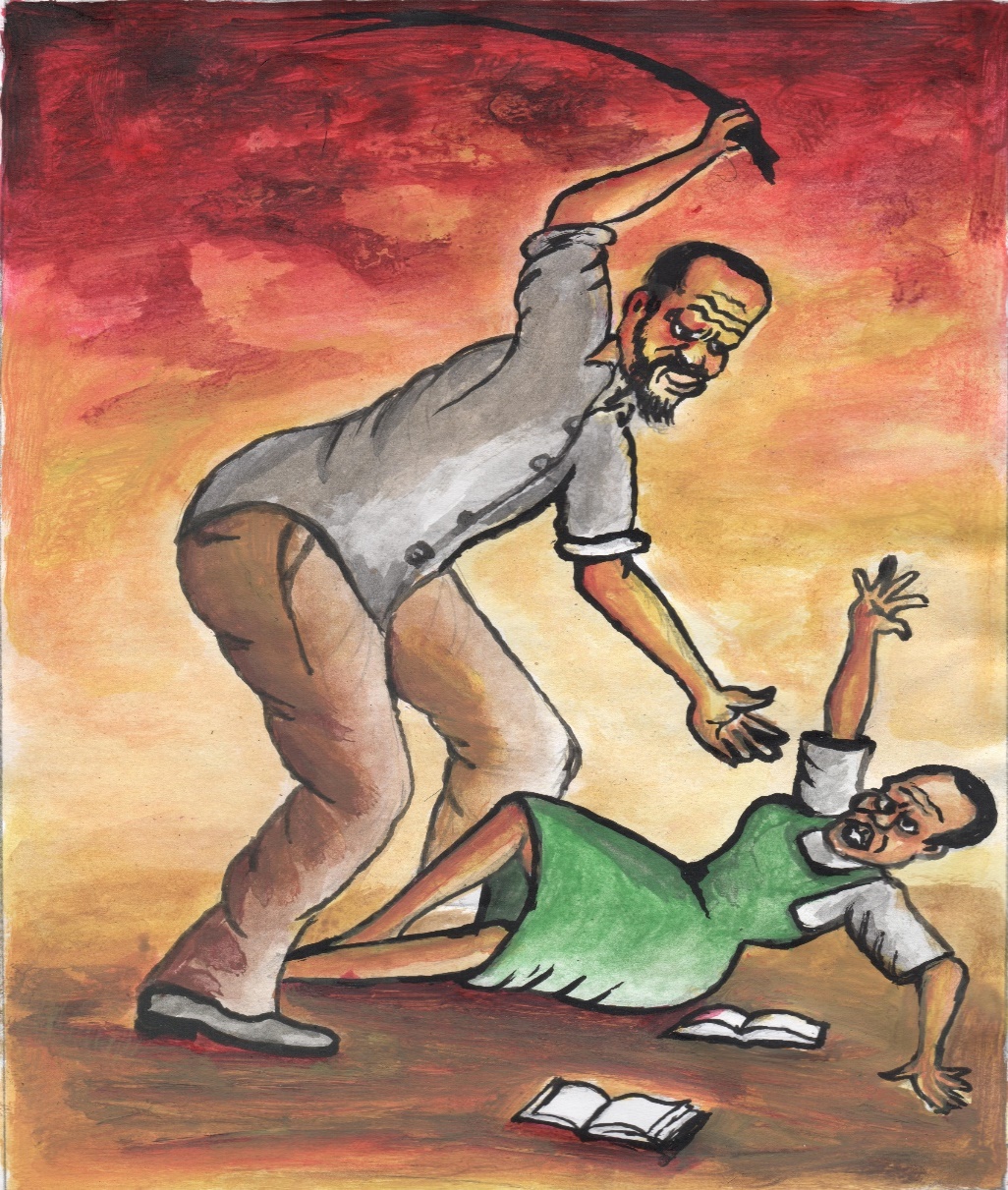 | **PHYSICAL ABUSE**   - In the past year, have you been hit, kicked, or physically hurt by another person?   Mchaka chathachi, mwapangidwako nkhanza kapena kumenyedwa ndi wina aliyense.   - If yes, can you describe what happened. - Mungafotokoze mmene nkhazazo zinachitikira. |
| 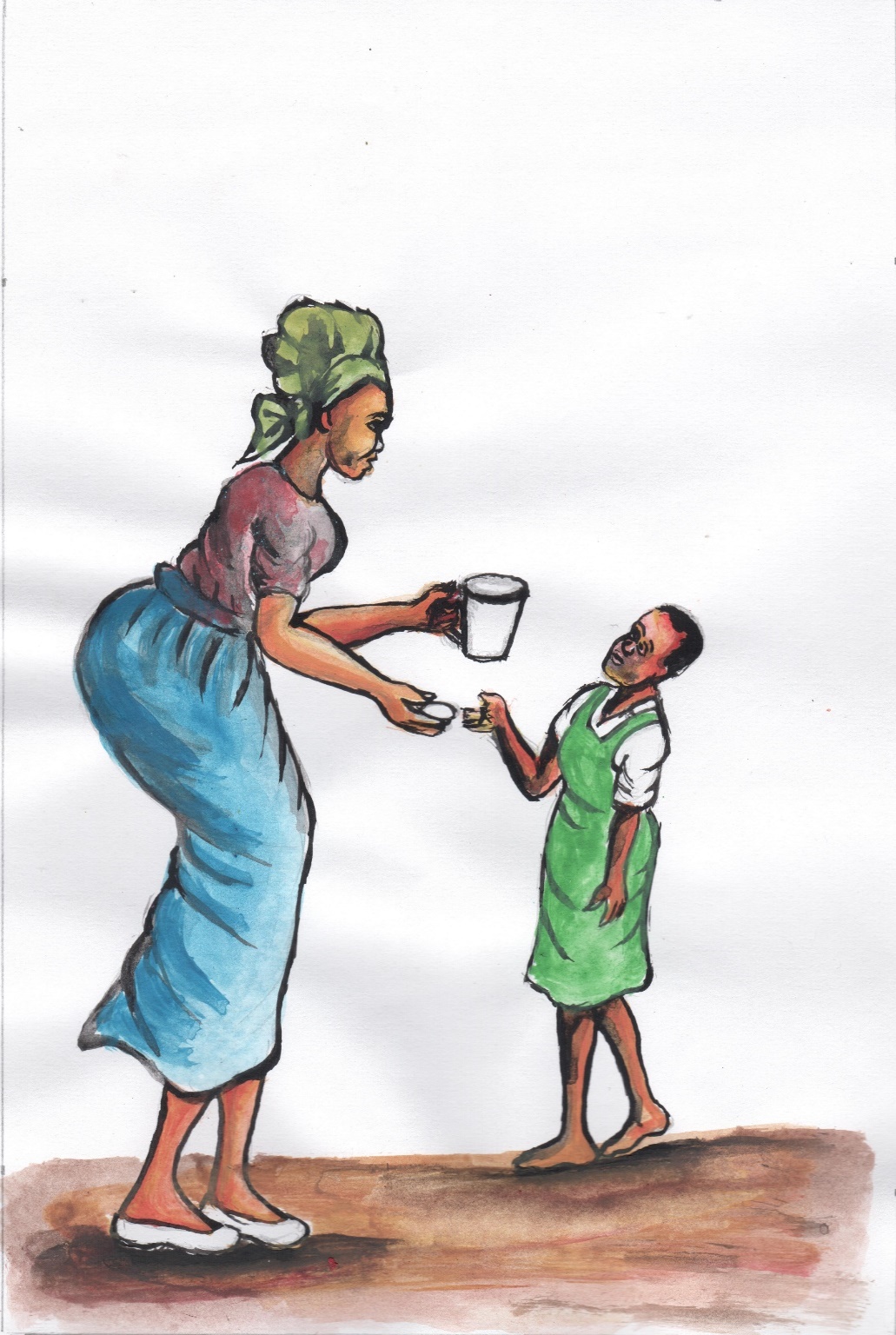 | **ADHERENCE TO ANTIRETROVIRAL THERAPY**   - Have you disclosed your HIV status to anyone outside of Teen Club and family?   Munawuzapo munthu wina aliyense za kuti muli ndi kachilombo ka HIV kupatula anzanu ena akuno ku teen club ndi aku banja kwanu.   - Who supports in adherence to your medications?   Amene amakulimbikitsani ndani kutsata ndondomeko ya kamwedwe ka mankhwala anu?   - How often do you miss drugs?   Mumadumphitsa kumwa mankhwala mowirikiza bwanji?   - How do you take your medications in boarding school (Only for those attending boarding school)   Nanga mukakhala ku sukulu, mamwedwe anu amakhwala amakhala otani? (Kwa okhawo ali ku sukulu yogonera konko) |
| 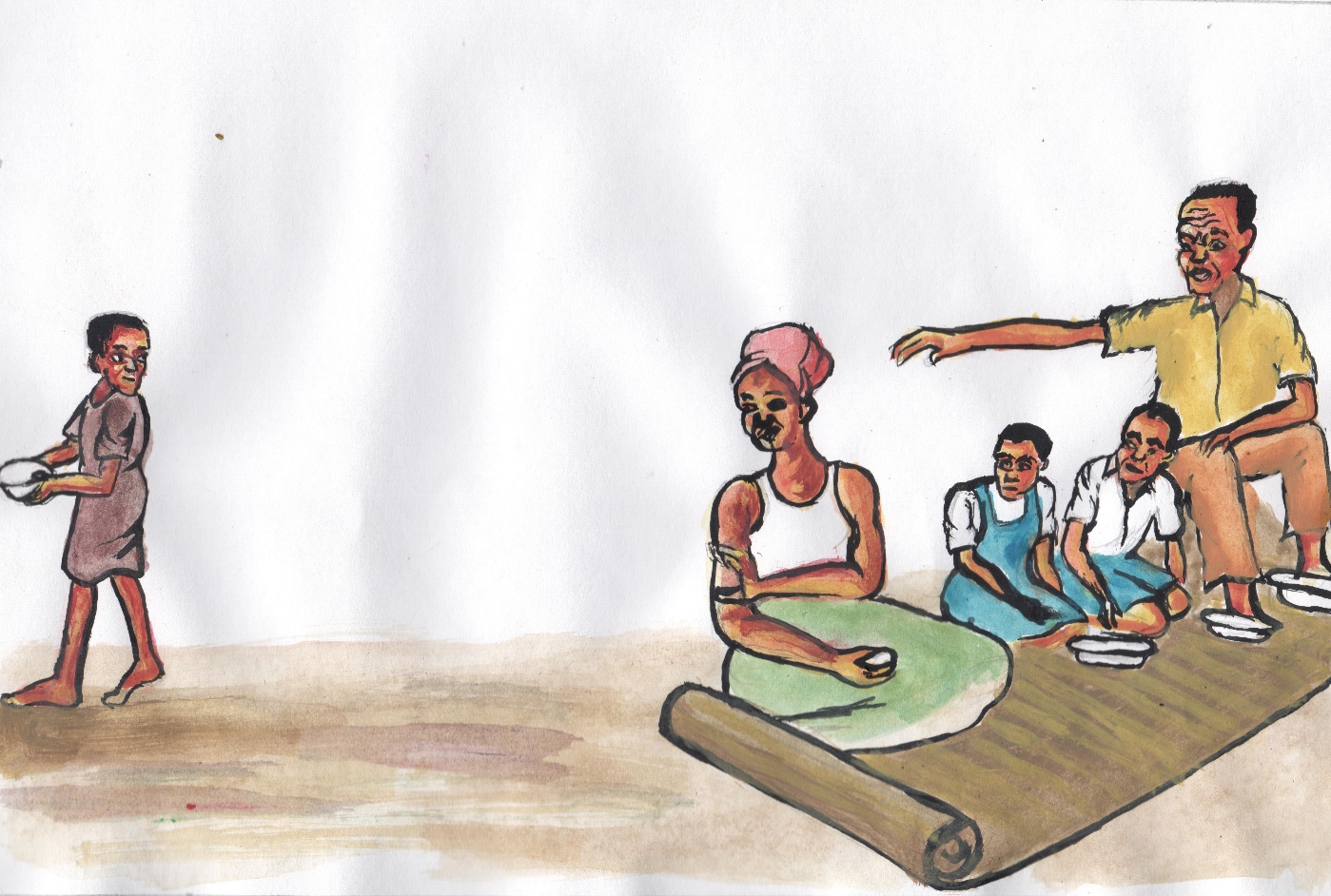 | **STIGMA AND DISCRIMINATION**   - Do you feel discriminated, bullied and stigmatized in any way because of your HIV status?(in the community, home or at school)   Kodi mumadzimva kusankhidwa, kutonzedwa kapena kusalidwa munjila ina iliyonse chifukwa muli ndi kachilombo ka HIV (kudela kwanu, kunyumba kapena kusukulu)   - What difficulties do you face because of what people say about you at school or at home?   Kodi mumakumana ndi mavuto anji, Kamba ka zoyankhula za anthu ku sukulu kapena kunyumba?   - Any ART or HIV effects that you are worried about (lipodystrophy or gynecomastia)?   Pali zosintha za thupi lanu zokhudzana ndi kumwa mankhwala zomwe zikuku dandaulitsani? |
| 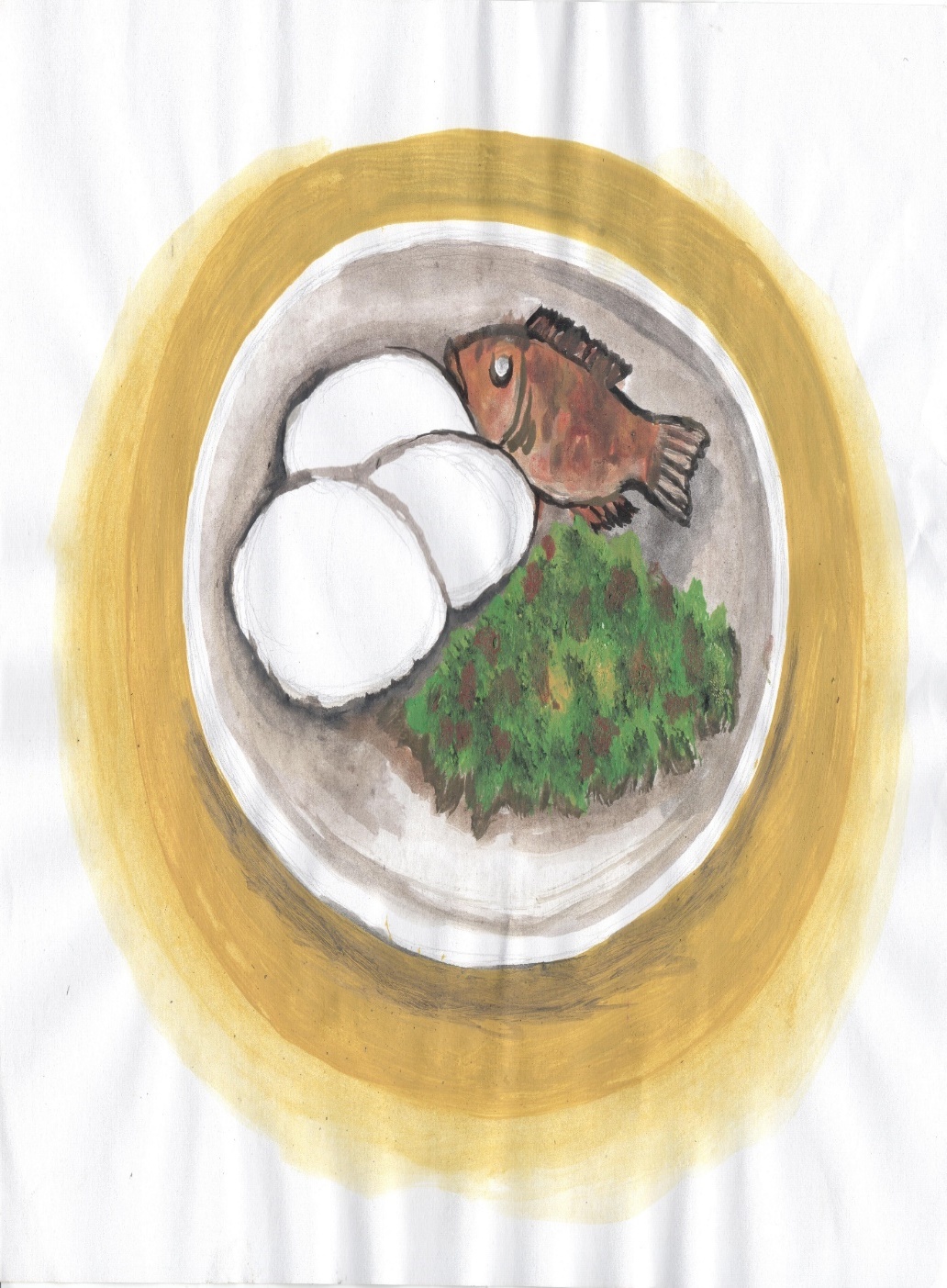 | **FOOD SECURITY**   - What do you normally eat for breakfast, Lunch and Supper?   Mungandifotokozereko zakudya zomwe mumanya patsiku; mmawa , masana ndi madzulo.   - Do you normally eat what you want at home?   Mumadya zakudya zomwe inu mumafuna?   - What is the source of the food in the home?   Zakudya zimenezi mumazipeza bwanji? |
| E – EDUCATION AND EMPLOYMENT | |
| 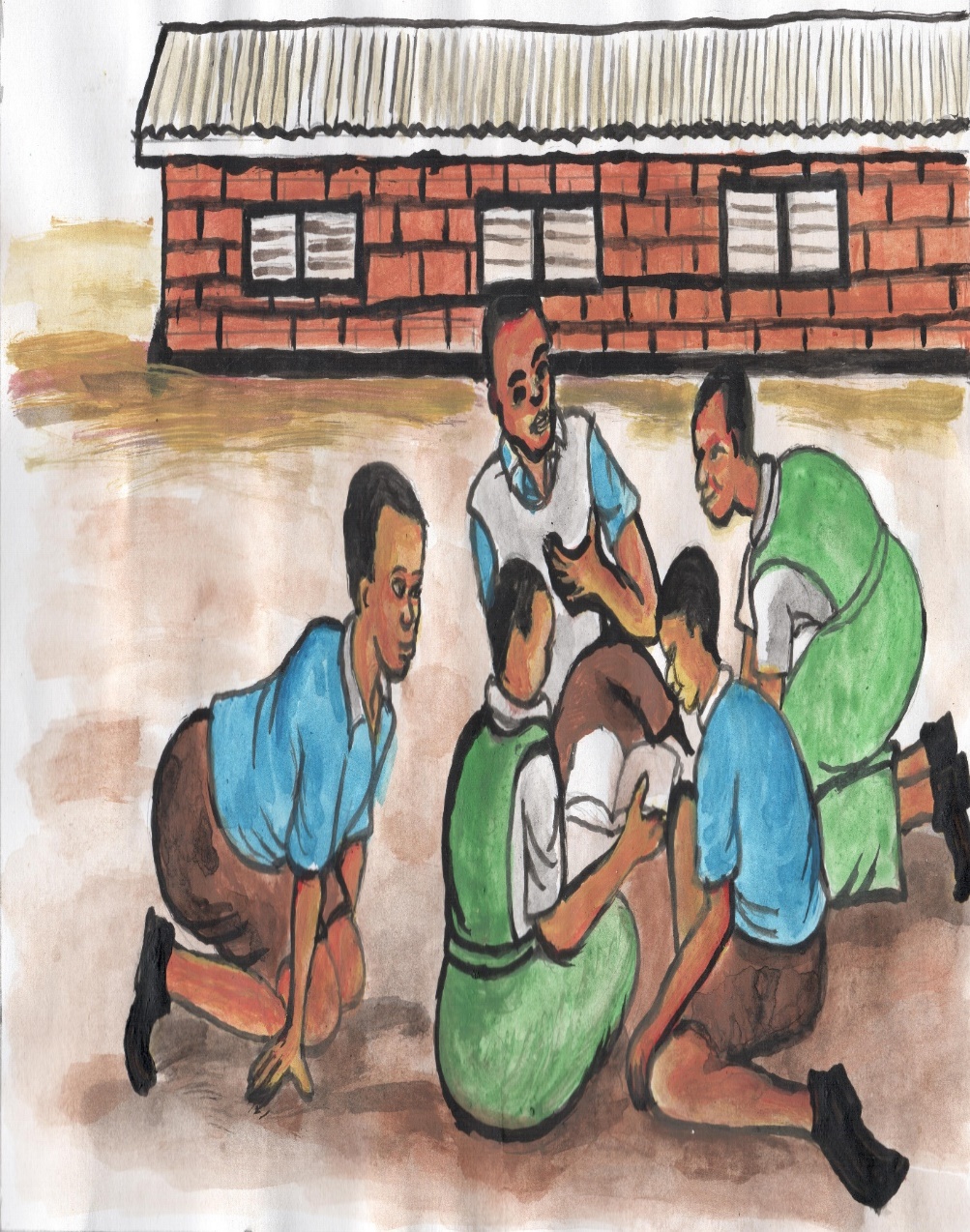 | **SCHOOL ISSUES**   - Do you go to school?   Kodi muli pa sukulu?   - Educational level? (Primary, secondary and tertiary/boarding or day)?   Kodi muli ku pulayimale, sekondale kapena sukulu ya ukachenjede/yoyendera kapena yogonera pompo?   - Who pays for your school fees, uniform, and school materials?   Kodi amakulipilirani fizi, kukugulirani uniform ndi zofunikira ku sukulu ndani?   - What’s your experience at school? (Inquire about “bullying”).   Kodi mmakumana ndi zotani ku sukulu? (Munayamba mwatonzedwapo?   - Have you ever had to repeat a class, suspended or expelled? Why?   Kodi munabwerezapo kalasi, kuyimitsidwa kapena kuchotsedwa? Chifukwa chiyani?   - Have you missed classes this month/ quarter/semester? (***reasons for missing school*)**   Kodi mwajombako ku sukulu mwezi uno, miyezi itatu yapitayi kapena teremu yathayi? (pali zifukwa zanji?)   - Have you ever considered dropping out of school?   Munayamba mwalingalirapo zosiya sukulu?   - Is there anyone at school you feel you could talk to about something important? (Who?)   Pali wina wake amene mumakhala omasuka naye ku sukulu, yemwe mumakamba naye zinthu zofunikira? (ndipo ndi ndani?)   - What do you want to do when you finish school? Any future plans/career or vocational goals?   Kodi mumafuna kudzapanga chiyani mukamaliza sukulu. Muli ndi malingaliro anji atsogolo lanu pa ntchito yomwe mumafuna kudzagwira?  **EMPLOYMENT ISSUES**   - Do you work after school or on weekends? Where? Paid or unpaid?   Kodi mumagwira ntchito ina iliyonse mukakhala kuti simuli ku sukulu? Kuti? Mumalipilidwa kapena ayi?   - How do you get along with your employers?   Kodi mumakhalitsana nawo bwanji amene anakulembani ntchitowo? |
|  |  |
| A – ACTIVITY | |
| 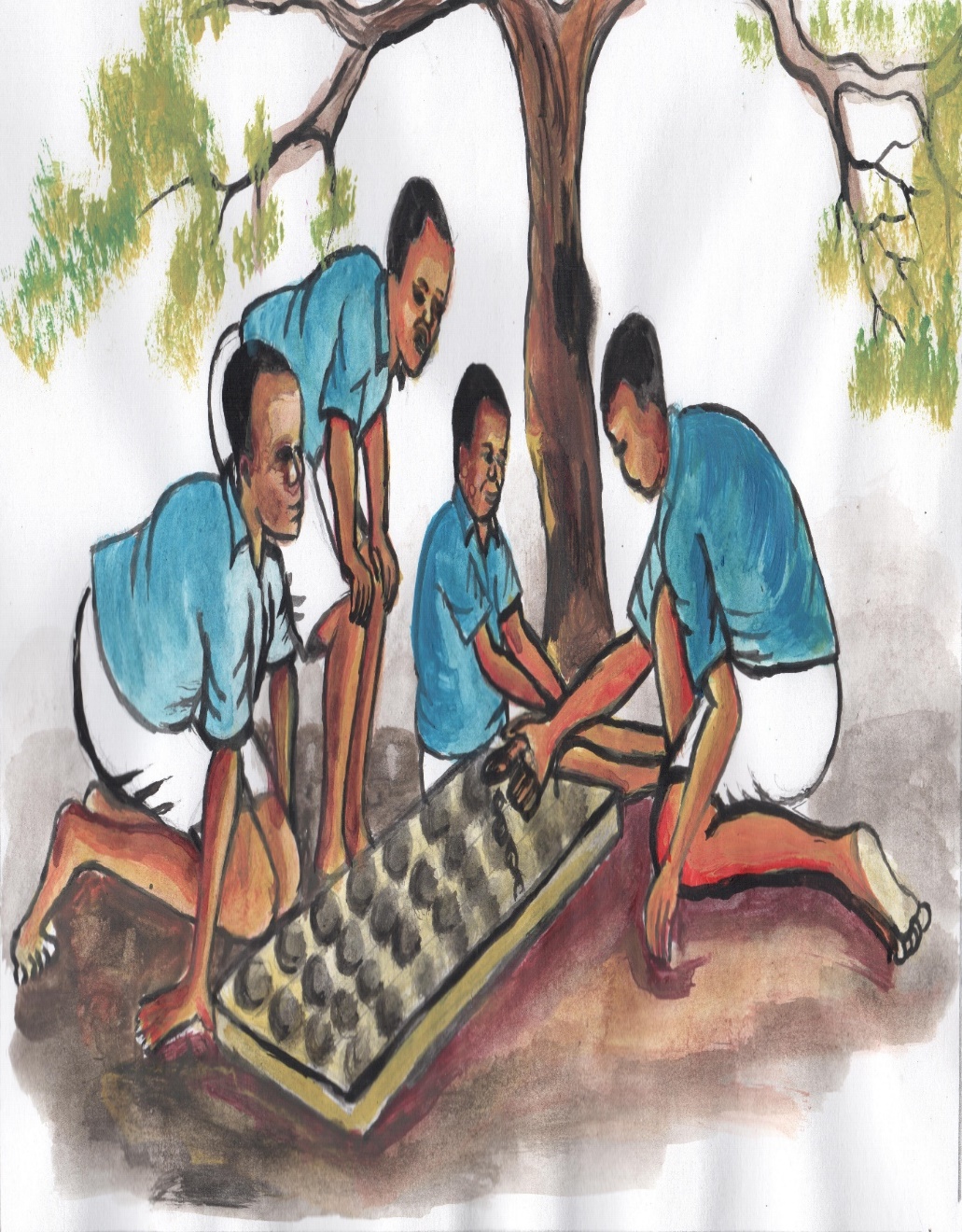 | - How do you spend your time? (Youth club, games, football, netball etc)   Kodi nthawi yanu yapadera mumayigwiritsa ntchito bwanji? ( monga kupita ku magulu achinyamata, masewera olimbitsa thupi ngati mpira wa miyendo ndi wa manja)   - Are most of your friends from school or somewhere else? (age, sex, hobbies)?   Anzinzanu ambiri mulinawo ndi ochokera kusukulu kapena madela ena? (ali ndi zaka zingati, ndi anyamata kapena atsikana, nanga amakonda chiyani?)   - Do you regularly go to church or attend any religious activities?   Kodi mumapita kutchalitchi pafupi pafupi kapena kutenga nawo gawo pa zochitika za ku tchalitchi? |
| D - DRUGS | |
| 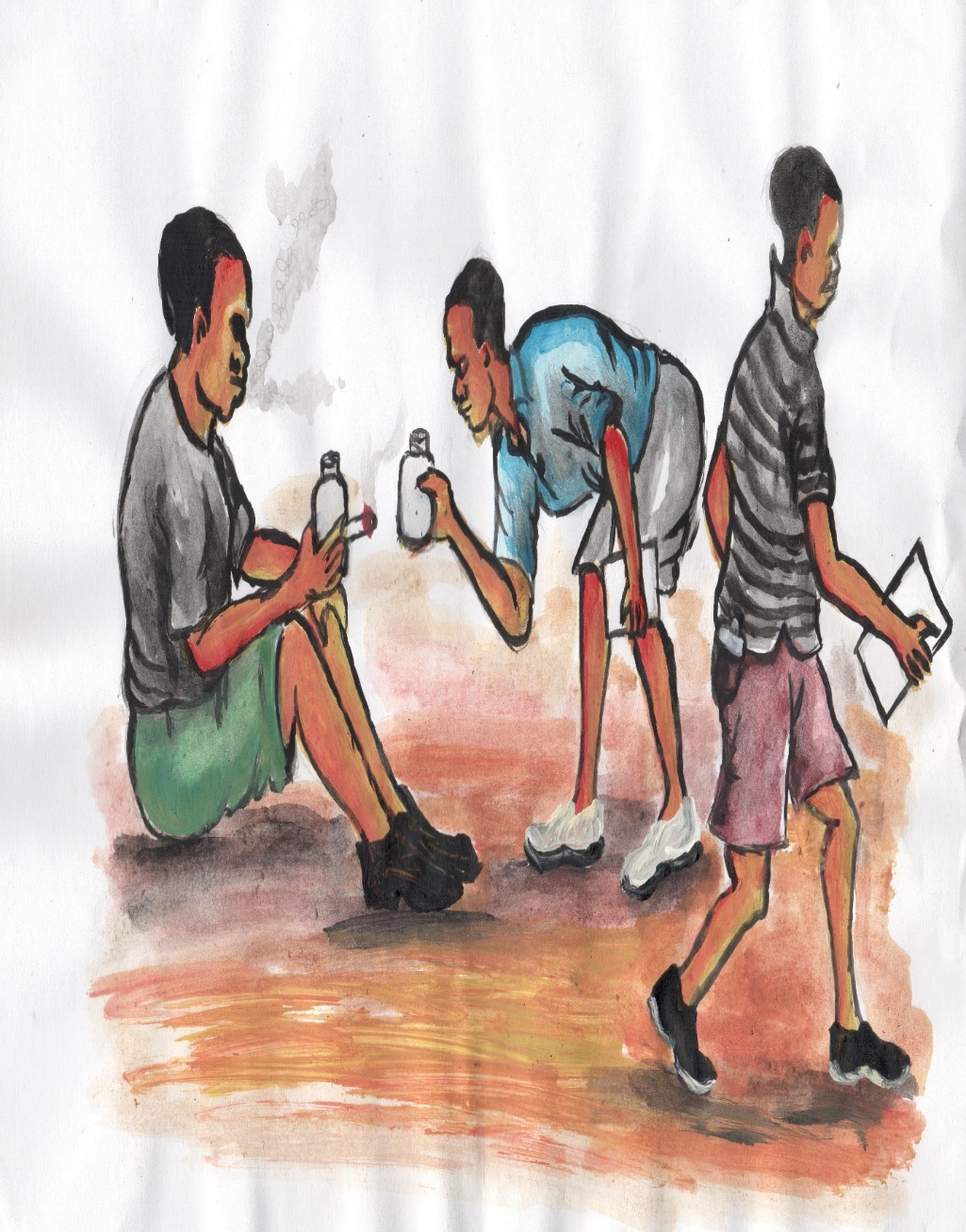 | Some young people experiment with drugs, alcohol, or cigarettes.  Achinyamata ena amagwiritsa ntchito mankhwala ozunguza bongo ngati fodya ndi mowa   - What do you know about substance abuse?   Kodi mukudziwapo chani pa mankhwala ozunguza bongo?   - Have you or your friends ever tried them?   (alcohol, chamba, cannabis, kuber or other drugs?  Kodi mudayamba mwagwiritsapo ntchito, kapena anzanu ena anayamba agwiritsapo ntchito mankhwala ozunguza bongowa? (monga chamba, Kuber)   - What do you think makes people to take drugs?   Ndi chifukwa chiyani anthu amatenga mankhwala ozunguza bongowa?   - How about you? Do you use any of these and how often? Nanga inu munayamba mwagwiritsapo ntchito mwankhwala ozunguza bongowa? |
| S-SEXUAL AND REPRODUCTIVE HEALTH | |
| 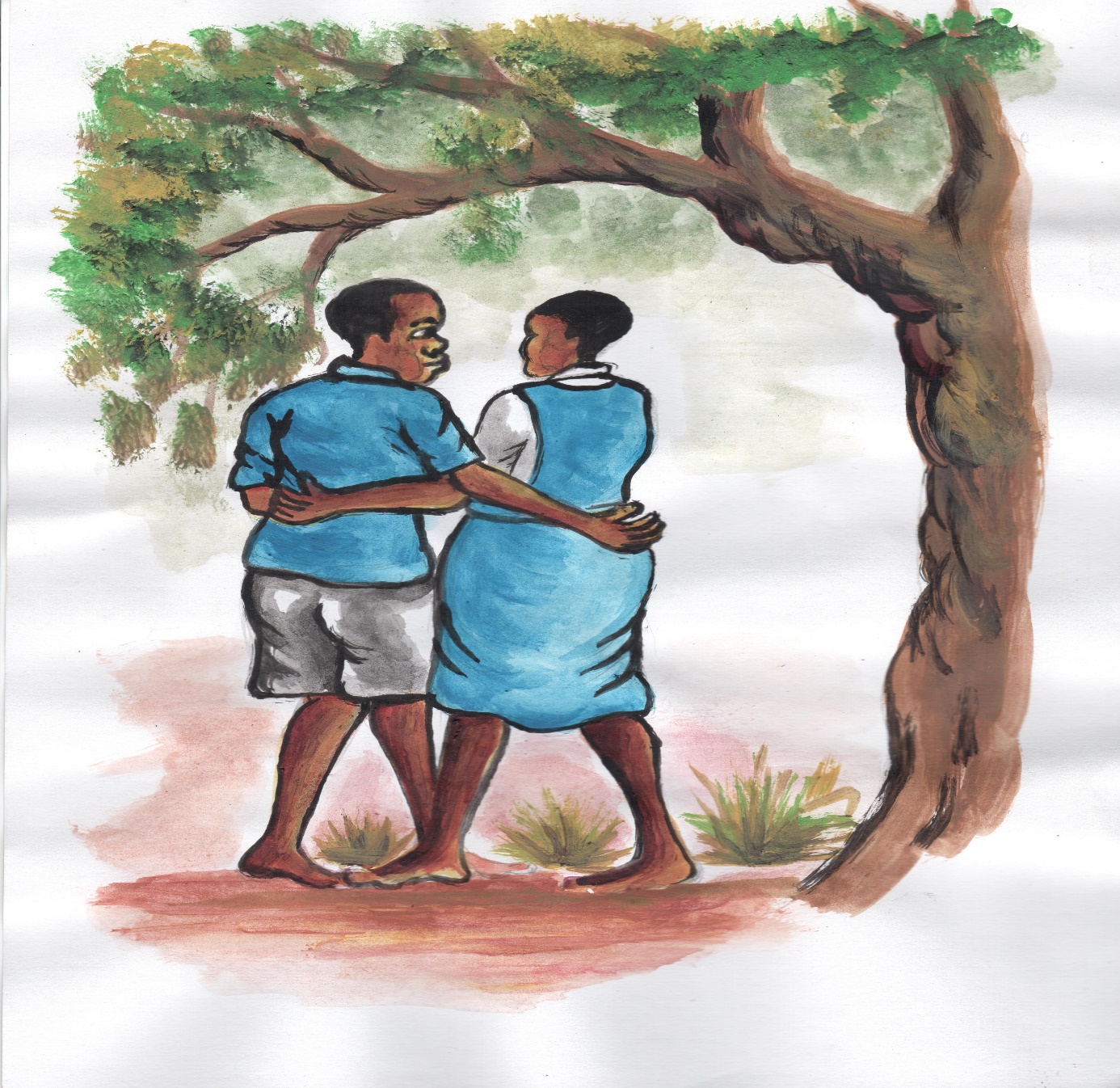 | Some young people are involved in sexual relationships  Achinyamata ena amapanga mchitidwe ogonana pa ubwenzi.   - Have you ever heard of anyone among your friends having a sexual relationship?   Munayamba mwawamverapo anzanu ena kuti amagonana ndi abwenzi awo?   - Have you been in an intimate relationship and are you comfortable with it?   Nanga inu munayamba mwakhalapo ndi chibwenzi chomwe mumagonana nacho ndipo ndinu omasuka?   - How many sexual partners have you had? Has any of them been abusive? How and how did you deal with it?   Munayamba mwagonapo ndi anthu angati? Pali wina anakuchitani nkhanza? Anakuchitani nkhanza motani ndipo munachitapo chiyani?   - Have you disclosed your HIV status to your partner (s)?   Munayamba mwafotokozerapo abwenzi anu muli ndi kachilombo ka HIV?   - Do you know the HIV status of your partner?   Nanga abwenzi anu anakuwuzani ngati ali ndi kachilombo koyambitsa HIV kapena ayi?   - Have you ever tried to exchange sex for money or any material things?   Munayamba mwagonanapo ndi munthu ndi cholinga choti akupatseni ndalama kapena zimene zinthu mumasowa?   - If sexually active have you ever had a discharge or sores that you are concerned about or worried that you had an infection?   Ngati munayamba mwagonapo ndi abwenzi anu, munatulukapo ukazi kapena umuna omwe udakudabwitsani kuti mwina mwatenga matenda.   - What does the term “safer sex” mean to you?   Kodi mukudziwapo chiyani pa nkhani yogonana modziteteza?   - Have you ever heard of condoms or other forms of contraception to prevent against sexually transmitted infections (STI) and/or pregnancy?   Munayamba mwamvapo za ma condom kapena njira zina zodzitetezera mimba kapena matenda opatsirana pogonana?   - Have you ever used either of the mentioned methods?   Munayamba mwagwiritsako ntchito njira zomwe mwatchulazo?   - Has anyone touched, forced or pressured to do something sexually that you didn’t want?   Kodi pali winawake anakugwirani thupi lanu mosayenera musakufuna, kapena kugonana nanu kumene musakufuna?   - Even though you haven’t had a relationship yet, are you interested in boys or girls? (Perhaps you’re not sure? Or have you chosen to remain abstinent)? (***For those not involved in sexual activities)*** - Ngakhale mwakamba kuti simunakhalepo ndi chibwenzi, muli ndi chidwi ndi anyamata kapena atsikana. (Kapena simunaganizire za nkhaniyi, kapena munachita chisankho chosala kaye). **Kwa amene sanayambe zibwenzi.** |
|  |  |
| **S - SUICIDE/DEPRESSION** | |
| 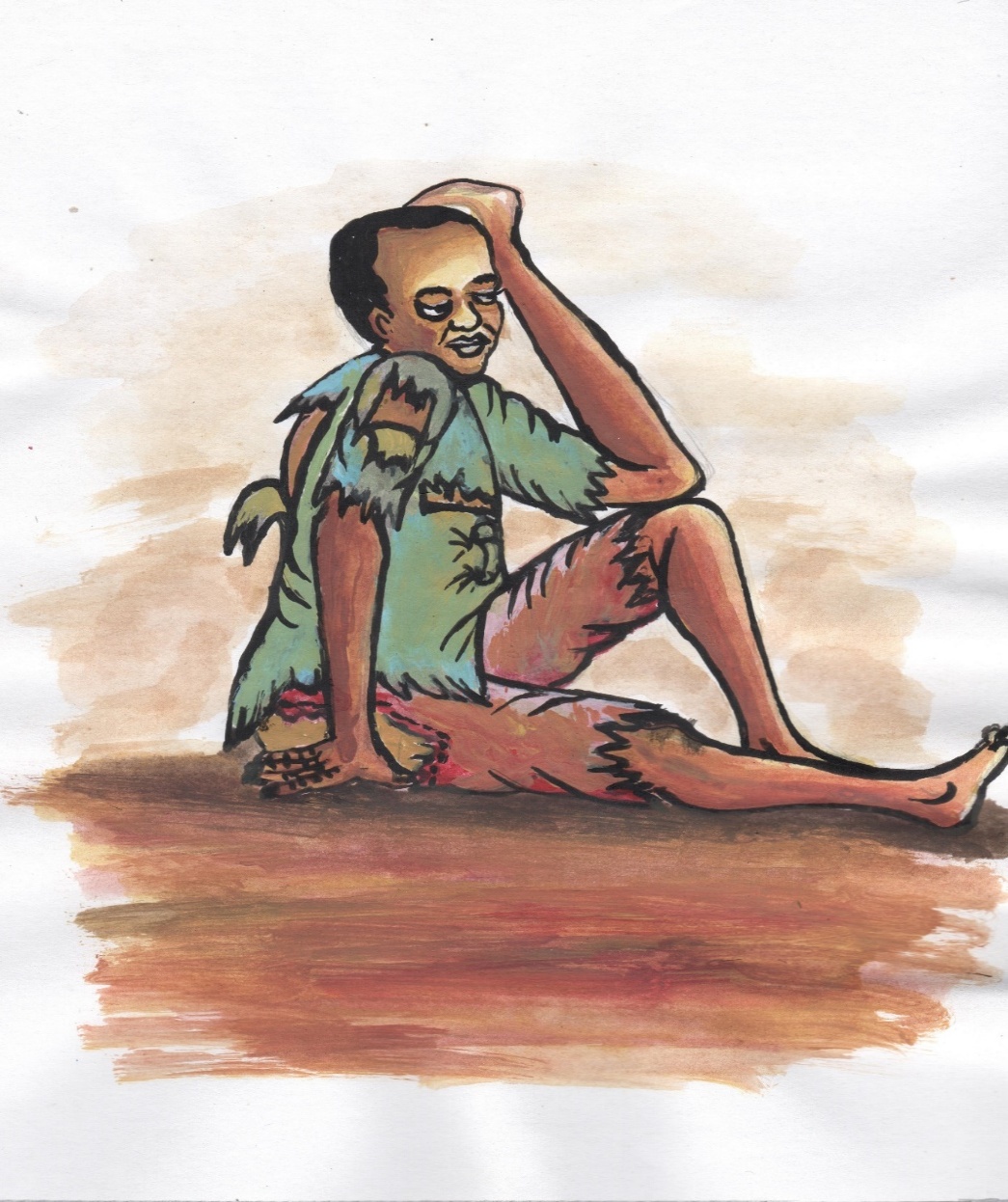 | **DEPRESSION ISSUES**  Many young people feel discouraged, sad and low.  Achinyamata ambiri amakhala opanda chilimbikitso, okhumudwa ndi osasangalala.   - Have you ever felt like this?   Munayamba mwakhalako mu mmaganizo oterawa.   - How often and for how long?   Zimachitika pafupi pafupi bwanji, ndipo kwanthawi yayitali bwanji?   - Do you ever have negative thoughts most often and why?   Kodi mumakhala ndi maganizo obwelera mbuyo pafupi pafupi? Chifukwa chani?   - Do you find yourself spending less and less time with friends or family? Why?   Pali nthawi zina zomwe mumawona kuti simukhalitsa kapena kucheza ndi anzanu ndi abale anu? Chifukwa chani?   - Does your behaviour affect your relationship with your family, friends, school and people in the community? (e.g. lots of arguments)   Kodi pali khalidwe lanu lina lomwe limasokoneza kukhala bwino ndi abale anu, anzanu a kusukulu ndi akudera kwanu, mwachitsanzo kukangana kangana)   - How is your appetite, motivation, sleeping pattern etc?   Kodi chilakolako chanu chodya zakudya, magonedwe anu ndi kulimbikitsidwa chili bwanji? |
| 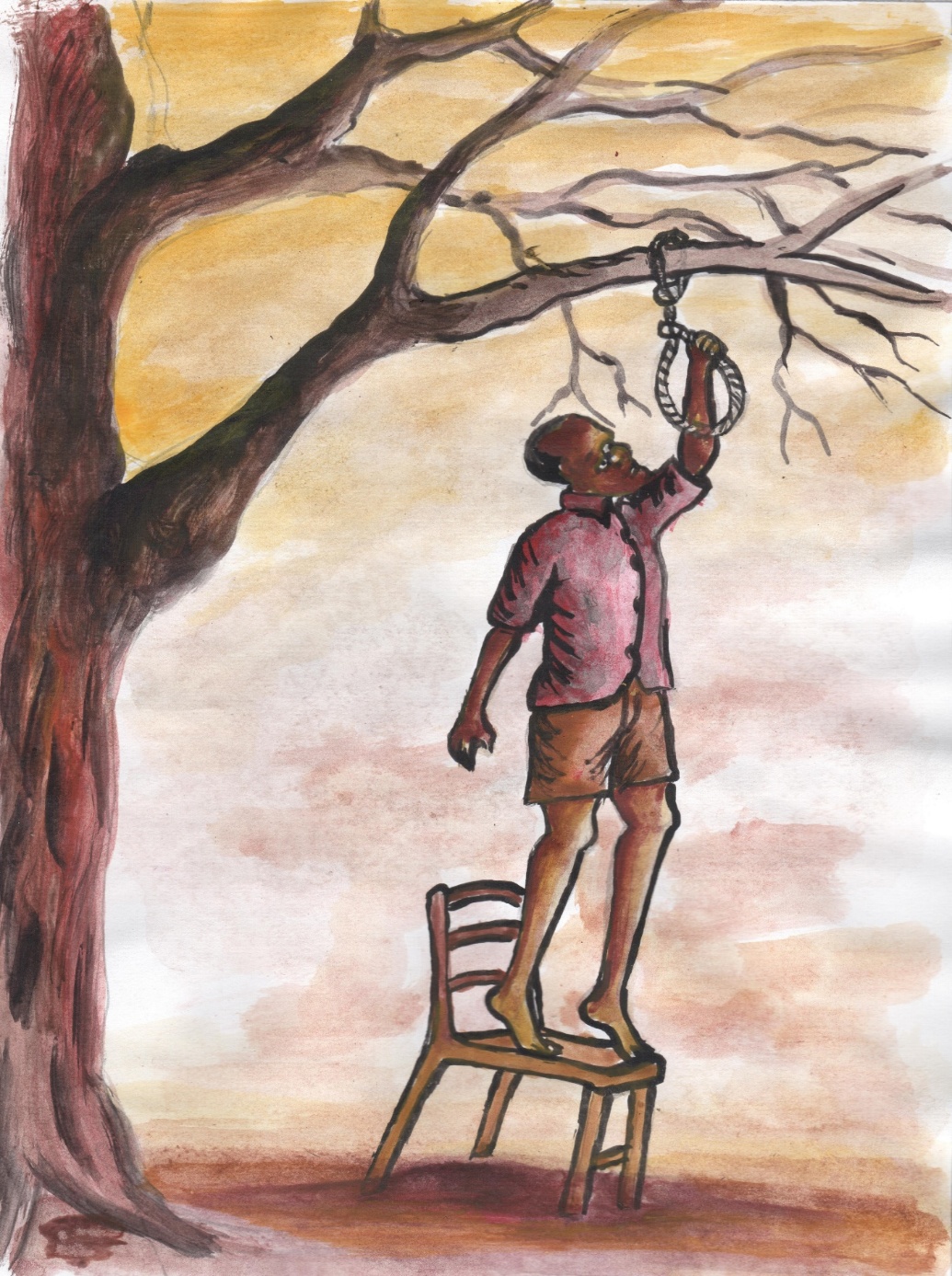 | **SUICIDE ISSUES**  Some people who feel really down often feel like hurting themselves.  Anthu ena amakhala okhumudwa kwambiri kufikira pofuna kuwononga moyo wawo.   - Have you ever harmed or injured yourself or someone else (like cutting, burning, or scratching yourself)?   Kodi munayamba mwadzipwetekapo nokha kapena kuganiza zopweteka munthu wina (kudzicheka, kuwotcha, kudzikanda)   - What prevented you from doing so?   Chidakubwezani maganizo ndi chani?   - Do you know anyone who has committed suicide?   Mukudziwapo munthu amene adatengapo moyo wake. |
