# Supplementary material for: Cultural adaptation of a psychosocial screening tool for adolescents living with HIV/AIDS attending antiretroviral therapy program in Malawi
Source: PLoS One. 2025 Nov 17;20(11):e0318738. doi: 10.1371/journal.pone.0318738 (PMC12622793; doi:10.1371/journal.pone.0318738)
Supplement: S1 File — English Focus Group Discussion Guide. S2 Text. Chichewa Focus Group Discussion Guide. S3 Text. Original HEADSS tool. S4 Text. Participants HEADSS adaptation notes_v1. S5 Text. HEADSS adaptation v1. S6 Text. Participants HEADSS adaptation notes_ v2. S7 Text. HEADSS adaptation v2. S8 Text. HEADSS adaptation v3. S9 Text. HEADSS adaptation _v4_Final Version. (ZIP) [file pone.0318738.s001.zip › Supporting Information/Supplementary File 9.docx]

Supplementary File 9
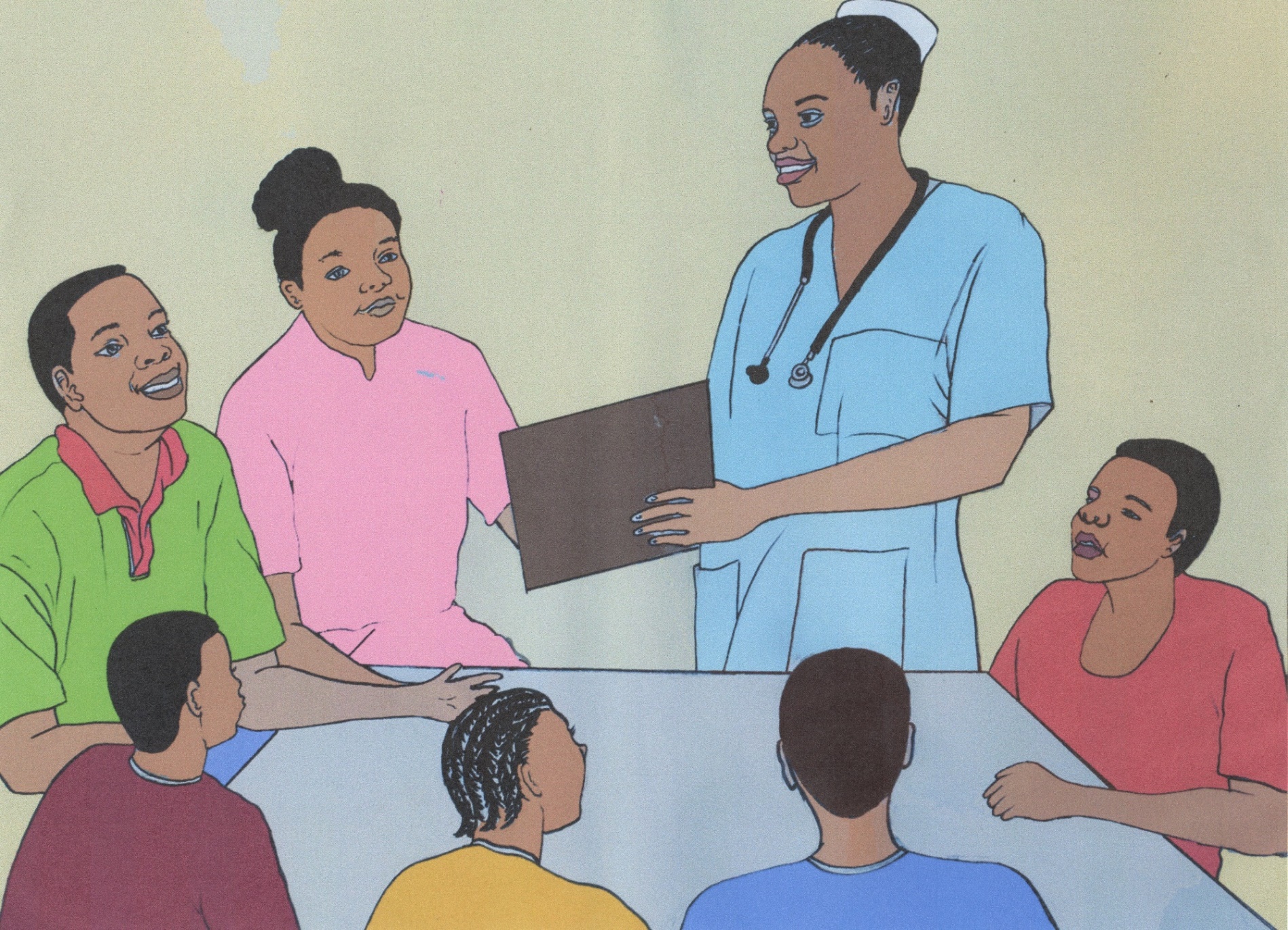
 - HEADSS Adaptation _V4_ Final Version


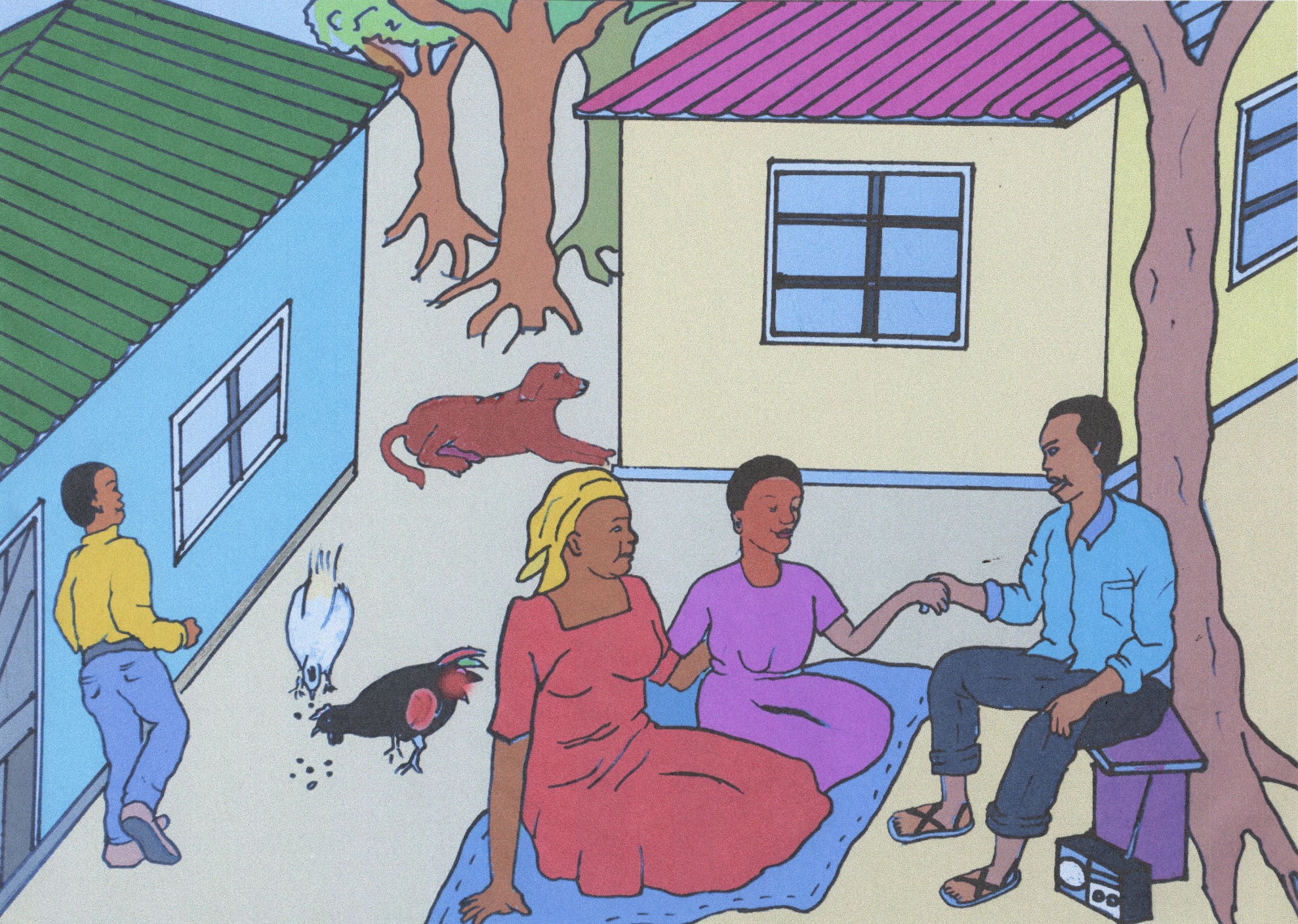


|  | **DOMAINS** | **INTERVIEWS QUESTIONS** |
| --- | --- | --- |
| **H – HOME AND ENVIRONMENT** | | |
|  | **ENGLISH** | **CHICHEWA** |
|  | **RELATIONSHIPS AT HOME** | **UBALE KU NYUMBA** |
| 1 | May I know your name and where do you stay? | Mungandiuze dzina lanu ndi komwe mumakhala? |
| 2 | Who do you live with? | Nanga mumakhala ndi ndani? |
| 3 | How do you get along with your relatives? | Nanga inuyo mumakhala nawo bwanji achibale anu? |
| 4 | What challenges do you face from where you live? | Ndi zovuta zanji zomwe mumakumana nazo kuchokera kumene mukukhala? |
| 5 | Have you ever had thoughts of leaving your home? | Munayamba mwakhalapo ndi maganiza ochoka komwe mukukhala? |
| 6 | If yes, why did you have such an idea? | Ngati eya, n’chifukwa chiyani munali ndi maganizo amenewa? |


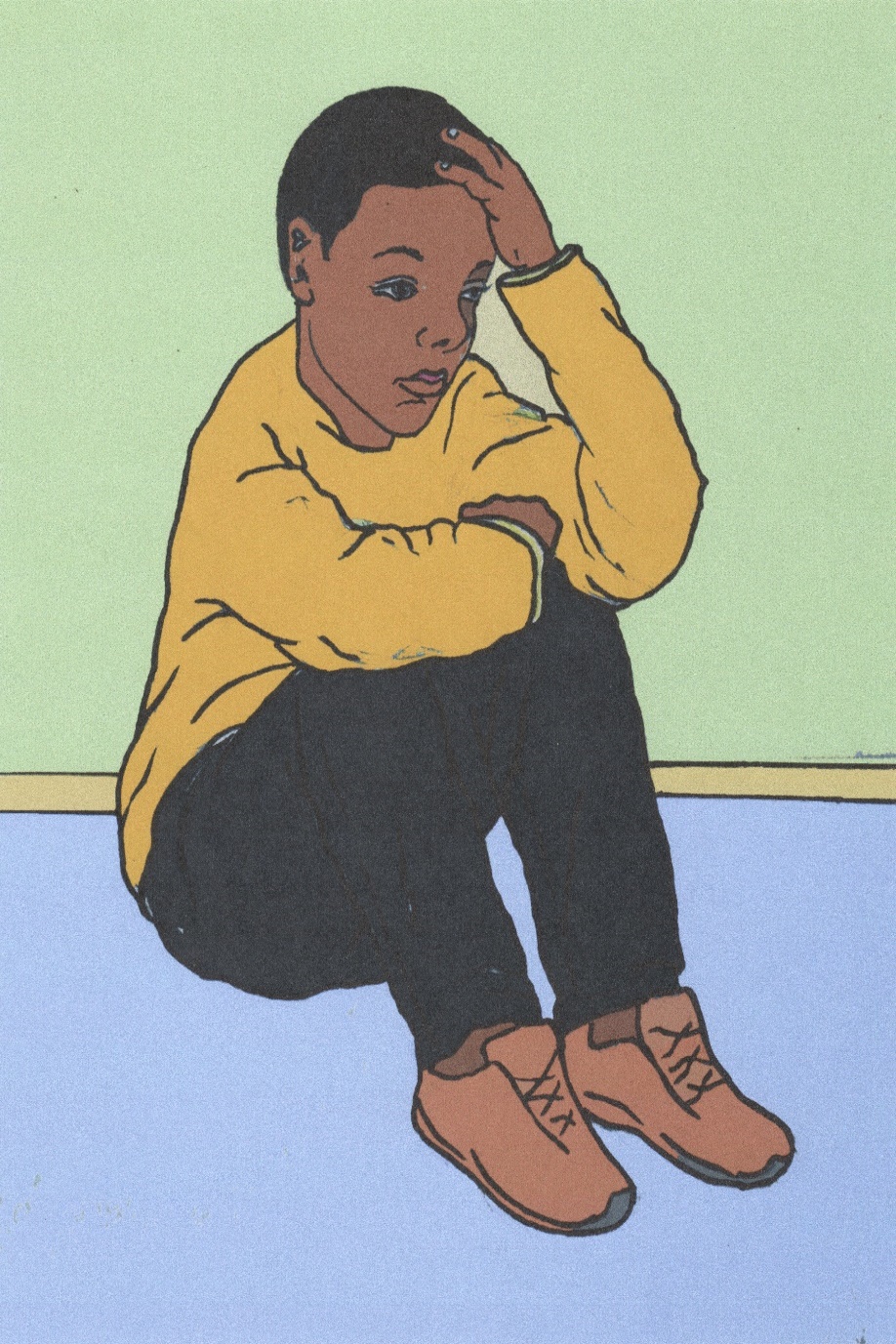


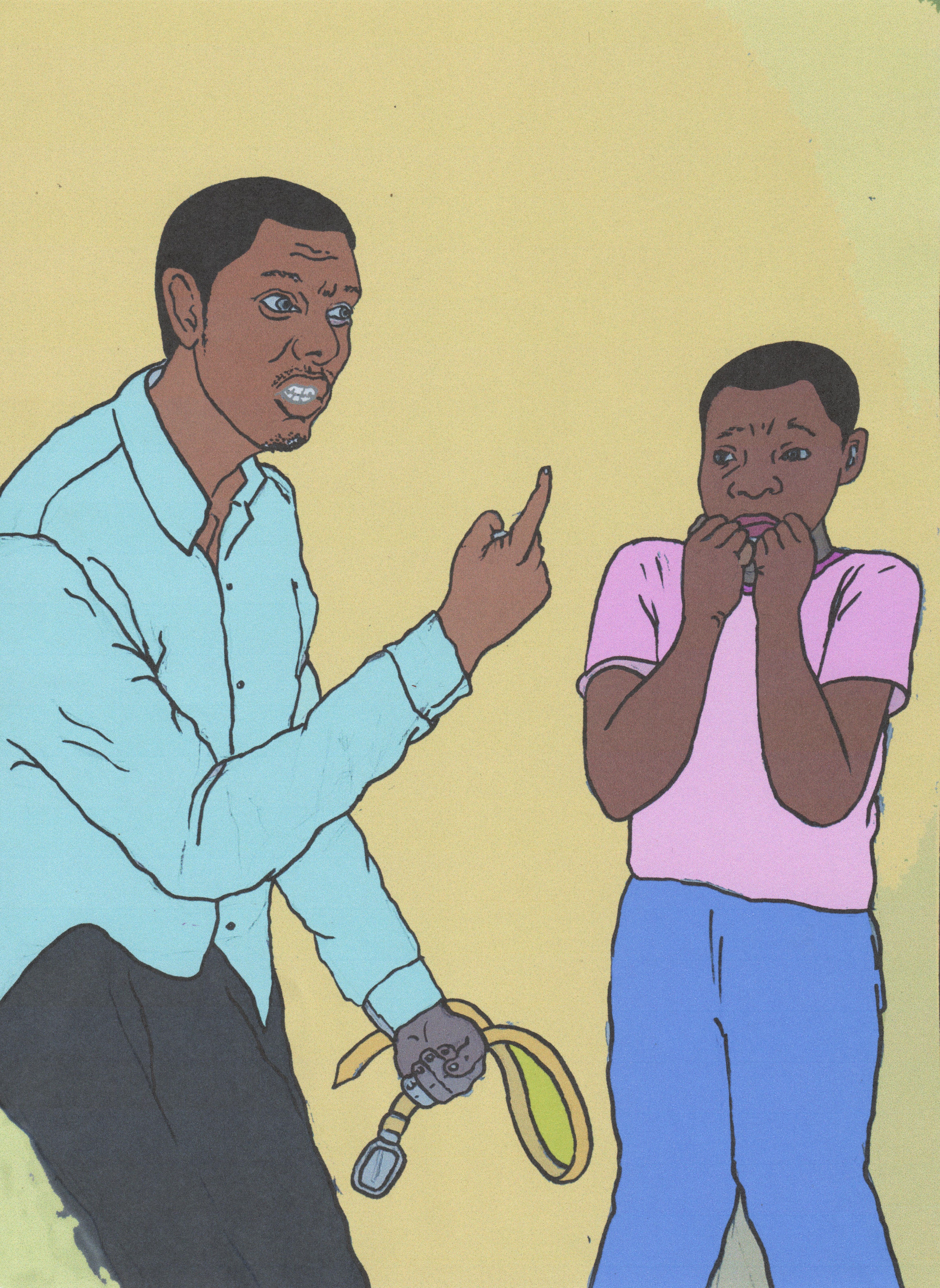


| **ABUSE/ NKHANZA** | | |
| --- | --- | --- |
| **1** | Have you ever been a victim of violence? | Kodi munayamba mwachitidwapo nkhanza? |
| **2** | If yes, may you explain what kind of violence it was | Ngati inde, tafotokozani zinali nkhanza za mtundu wanji? |
| **3** | Has anyone ever forced to have sex with you without your consent? | Kodi pali wina amene anakugwililiranipo kapena kukukakamizani kuti agonane nanu popanda chilolezo chanu? |
| **4** | If yes, what did you do after being abused? | Ngati eya, munatani mutachitiridwa nkhanza? |
| **5** | Where did you report after being abused or did you ever tell anyone else? | Kodi mudakchita lipoti kutiko mutachitiridwa nkhanza kapena munauzapo wine ali yense? |


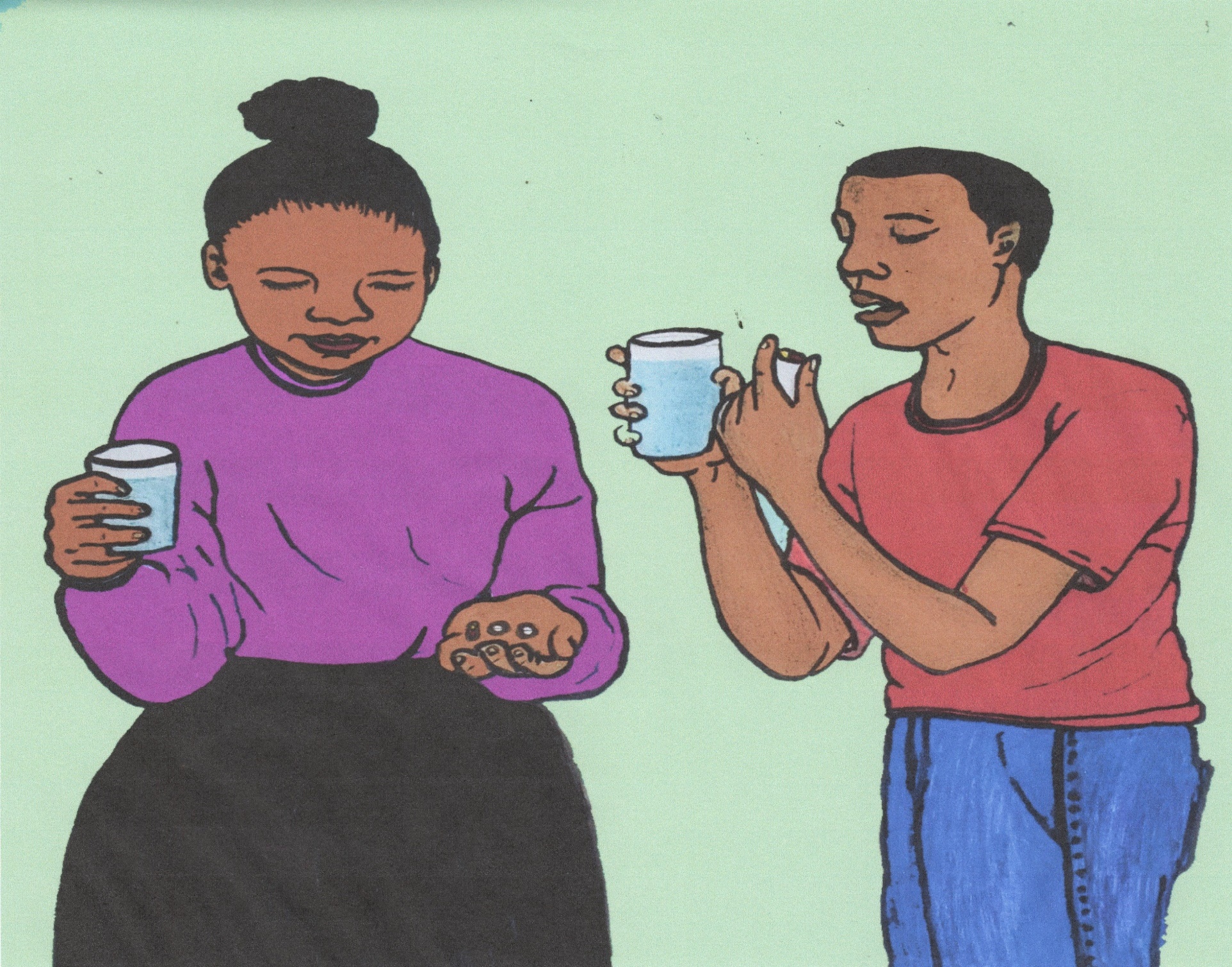


| **ADHERENCE TO ANTIRETROVIRAL THERAPY /KUMWA MANKHWALA MWA NDONDOMEKO** | | |
| --- | --- | --- |
| **1** | Apart from your friends in Teen Club or your family, have you disclosed your HIV status to other people? (*for those in boarding schools)?* | Kupatula anzanu kuno ku Teen Club ndi akubanja kwanu, munawuzapo anthu ena za kuti muli ndi kachilombo ka HIV? (*kwa omwe ali kusukulu zogonera*)? |
| **2** | How do you manage to take your medications at school? (*Only for those in boarding schools*) | kodi mukakhala ku sukulu yogonera konko kamwedwe ka mankhwala anu kamakhala kotani? |
| **3** | What side effects have you noticed because of taking ARVs? | Kodi mukukumana ndi zovuta zanji chifukwa chokumwa ma ARV? |
| **4** | Does having these side effects make you feel depressed? | Kodi zovuta zomwe mumakumana nazozi zimakupangitsani kukhala okhumudwa? |
| **5** | If yes, so what do you do? | Ngati inde, ndiye mumatani? |


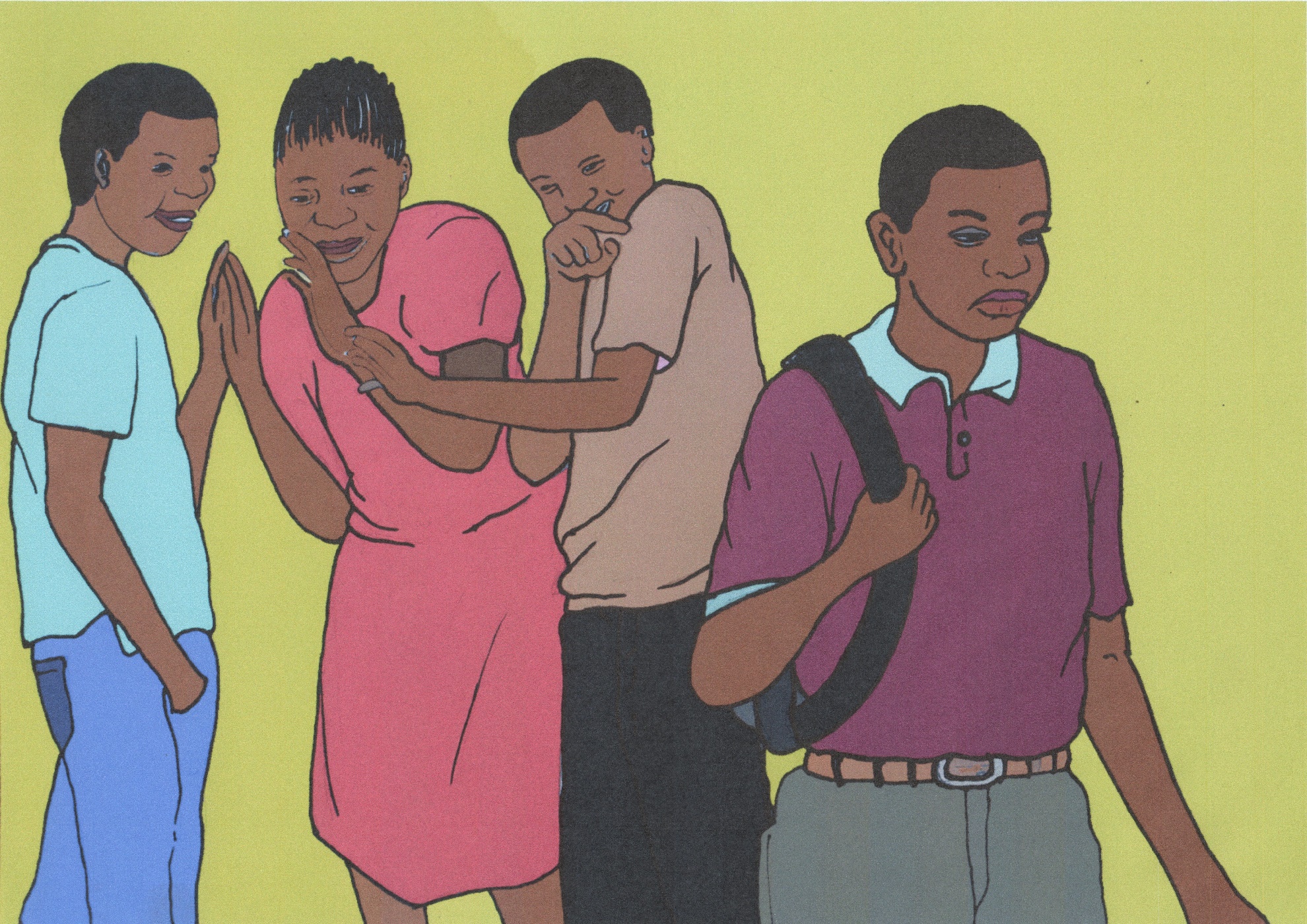


| **STIGMA AND DISCRIMINATION / KUSALIDWA** | | |
| --- | --- | --- |
| **1** | Do you feel discriminated, being bullied and stigmatized in any way because you are HIV positive?(in the community, home or at school)? | Kodi mumadzimva kuti mukusankhidwa, kutonzedwa kapena kusalidwa munjila ina iliyonse chifukwa muli ndi kachilombo ka HIV (kudela kwanu, kunyumba kapena kusukulu) |
| **2** | If yes, what difficulties/problems do you face because of what other people say about you at school or at home? | Ngati inde, ndizovuta ziti zomwe mumakumana nazo chifukwa cha zomwe anthu ena amanena za inu kusukulu kapena kunyumba? |
| **3** | Do you feel discriminated against during community or school events? | Kodi mumaona kuti mumakusalidwa panthawi ya zochitika za m'dera lanu kapena kusukulu? |
| **4** | What happens and how do you feel or how do you cope with that? | Kodi chimachitika ndi chiyani ndipo mumamva bwanji kapena mukulimbana nazo bwanji? |


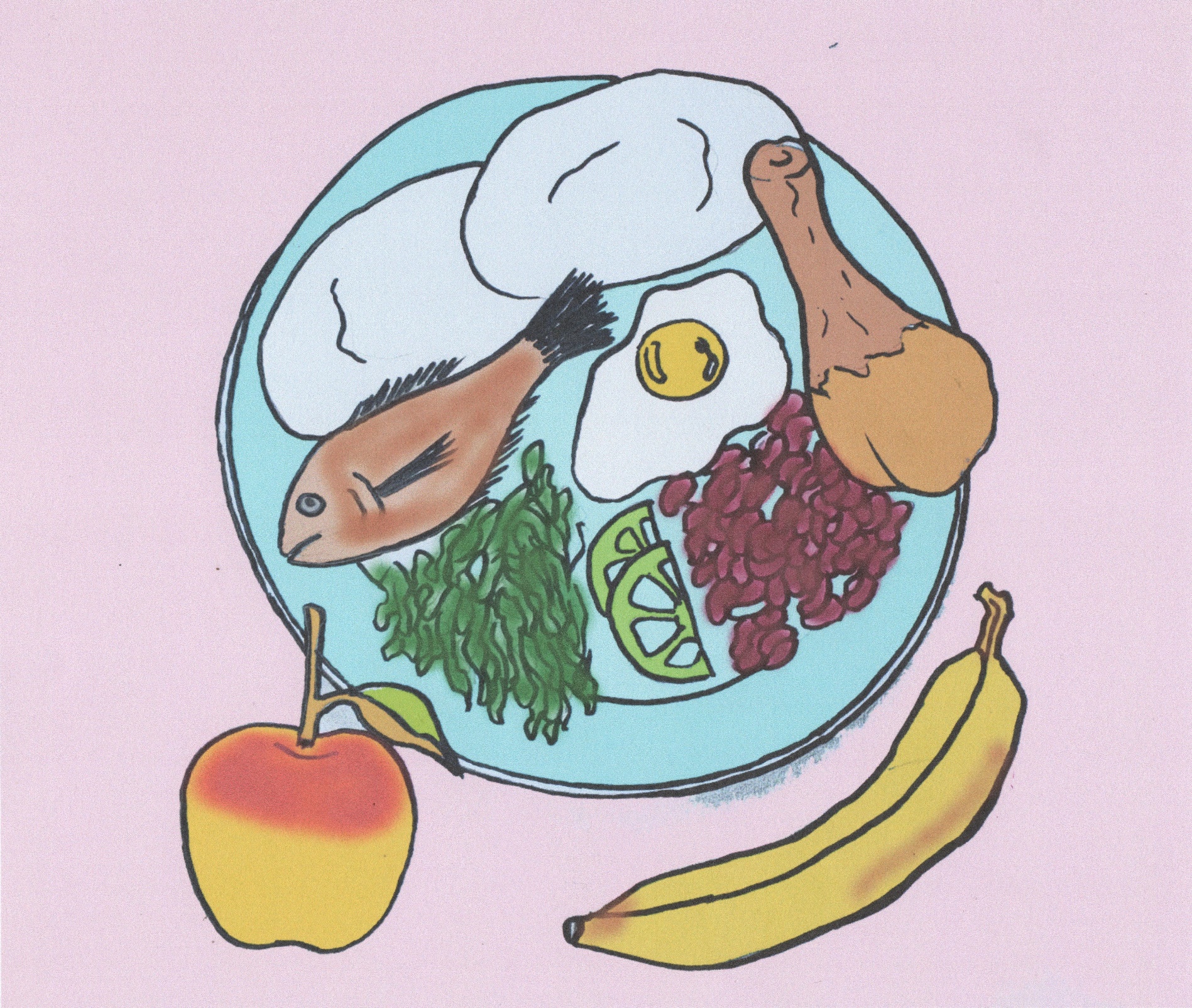


| **FOOD SECURITY** | |  |
| --- | --- | --- |
| **1** | How many times do you take food in a day? | Kodi mumadya chakudya kangati pa tsiku? |
| **2** | What type of foods do you take for breakfast, lunch and supper? | Ndi zakudya zotani zomwe mumadya m'mawa, masana ndi madzulo? |
| **3** | How do your parents get adequate food to feed the whole family? | Kodi makolo anu amapeza bwanji chakudya chokwanira banja lonse? |
| **4** | If food is not available, what happens? | Ngati chakudya palibe, chimachitika ndi chiyani? |


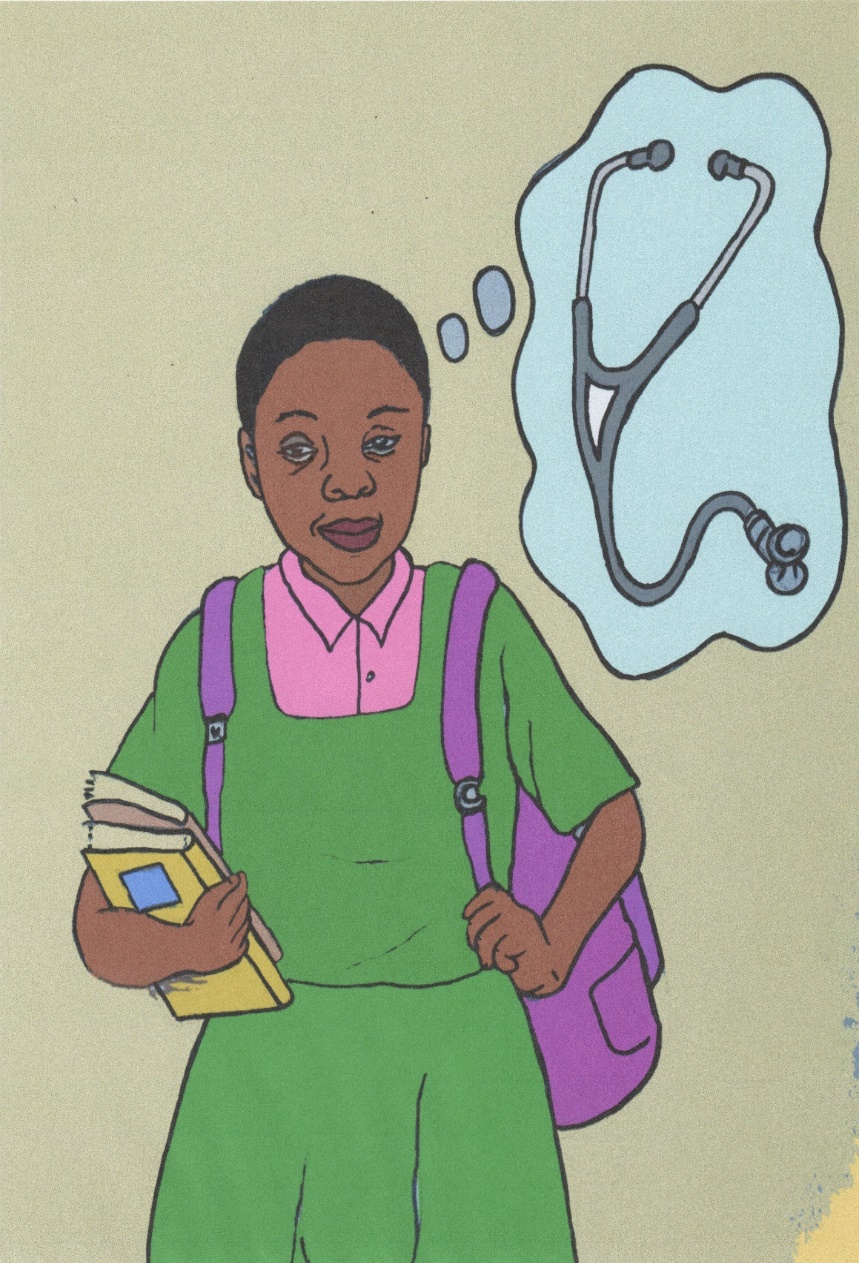

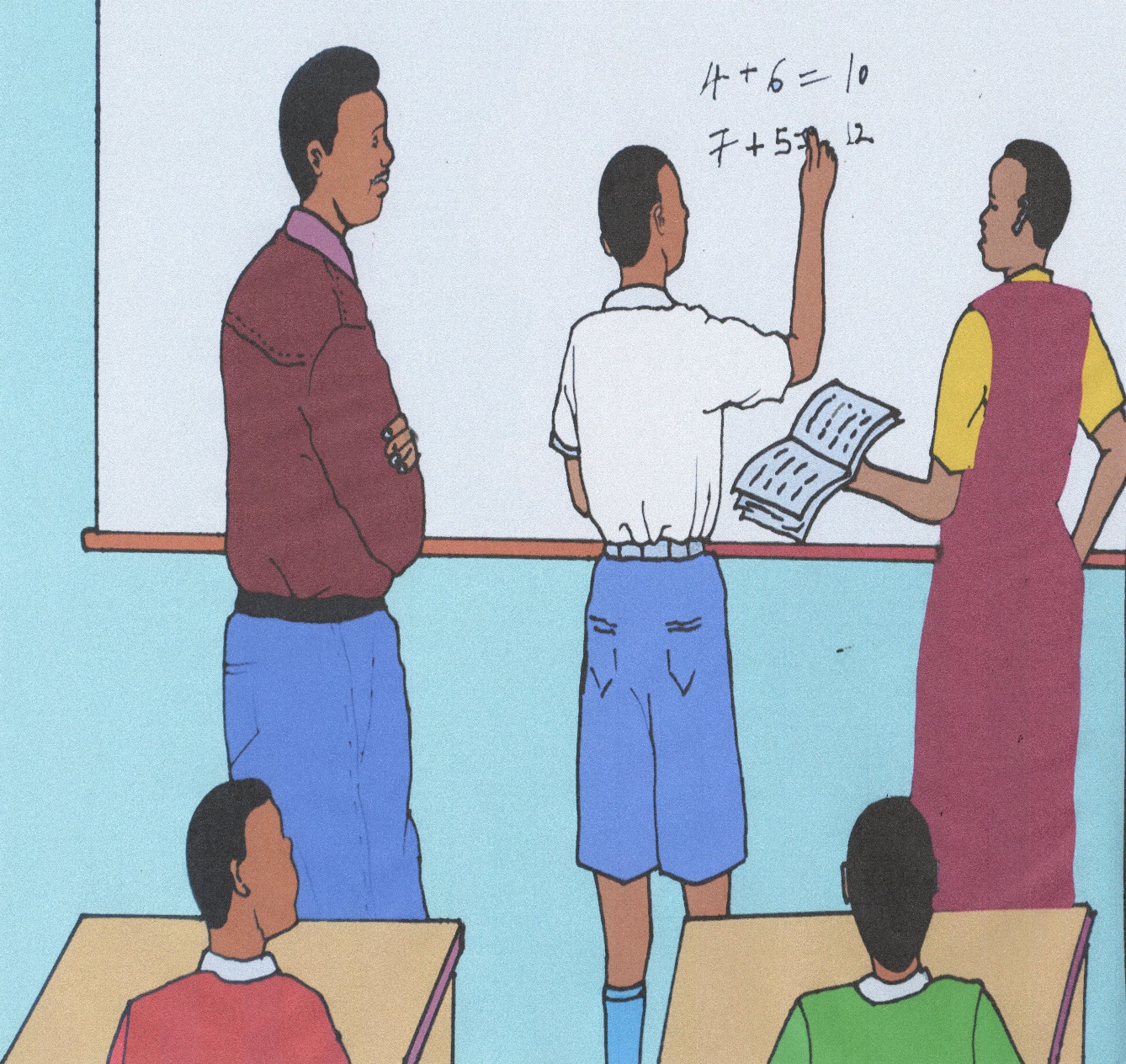


| **E – EDUCATION AND EMPLOYMENT** | | **MAPHUNZIRO NDI NTCHITO/GANYU** |
| --- | --- | --- |
| **1** | Are you in school? If yes, where do you go to school? | Kodi muli pasukulu? Ngati inde, mumapita ku sukulu itiyo? |
| **2** | Who pays for your books and other expenses? | Ndi ndani amakulipirani ndalama za mabuku ndi zina zofunikira ku sukulu? |
| **3** | What problems do you face because you are at school? *(If no problems, skip to Q.5)* | Kodi ndi zovuta zanji zomwe mumakumana nazo chifukwa choti muli pa sukulu? (*Ngati palibe zovuta, pitani ku funso No.5)* |
| **4** | So how do you deal with these problems? | Ndiye mumathana ndi mavutowa bwanji? |
| **5** | Have you ever been disciplined at school? *(If no, skip to No.7)* | Munayamba mwapatsidwapo chilango ku sukulu? *(Ngati ai, pitani ku funso No.7)* |
| **6** | If yes, what type of punishment did you get? | Ngati inde, kodi munalandira chilango chotani? |
| **7** | Have you been absent from school and if yes, why? *(Ask for the past 3 months)* | Munayamba mwajombapo ku sukulu? Ndi chifukwa chiani? (*Funsani kwa miyezi itatu yapitayi*) |
| **8** | Have you ever considered dropping out of school? | Kodi munayamba mwaganizapo zosiya sukulu? |
| **9** | If yes, why did you consider dropping out of school? | Ngati inde, n’chifukwa chiyani munaganiza zosiya sukulu? |
| **10** | When you are at school, who is your confidant that you can discuss freely with, about your HIV status and the ARVs you are taking? | Mukakhala kusukulu, ndani amene mumamukhulupirira yemwe mumakambirana momasuka za momwe mulili ndi kachilombo ka HIV komanso ma ARV omwe mukumwa? |
| **11** | What do you want to do when you finish school? | Kodi mumafuna kudzapanga chiyani mukamaliza sukulu? |
| **12** | What future plans do you have regarding your career? | Muli ndi malingaliro anji atsogolo lanu pa ntchito yomwe mumafuna kudzagwira? |
|  | **EMPLOYMENT (FORMAL OR INFORMAL)** | **NTCHITO (YOLEMBEDWA KAPENA GANYU)** |
| **13** | When you are not at school do you ever get a job somewhere to earn money? (If no, skip to the next section below) | Kodi mukakhala kuti simuli kusukulu mumapeza ntchito kwinakwake kuti mupeze ndalama? (Ngati ai, pitani ku gawo pansipo) |
| **14** | How much are you paid when you do some work for some people or in a company? | Kodi mumalipidwa ndalama zingati mukamagwira ntchito kwa anthu ena kapena pakampani? |
| **15** | How do you get along with the ones who employed you (or who give you piece works) | Kodi mumagwirizana nawo bwanji ndi omwe adakulembani ntchitowo (kapena omwe amakupatsani ganyu)? |
| **16** | Are you comfortable working full-time or part-time? | Kodi ndinu omasuka kugwira ntchito yokhazikika kapena nganyu? |
| **17** | How do you balance working and studying at the same time? | Kodi mumalinganiza bwanji ntchito ndi kuphunzira nthawi imodzi? |
| **18** | What made you seek for part-time work? | Chinakupangitsani kuti muzipeza ganyu ndi chiyani? |


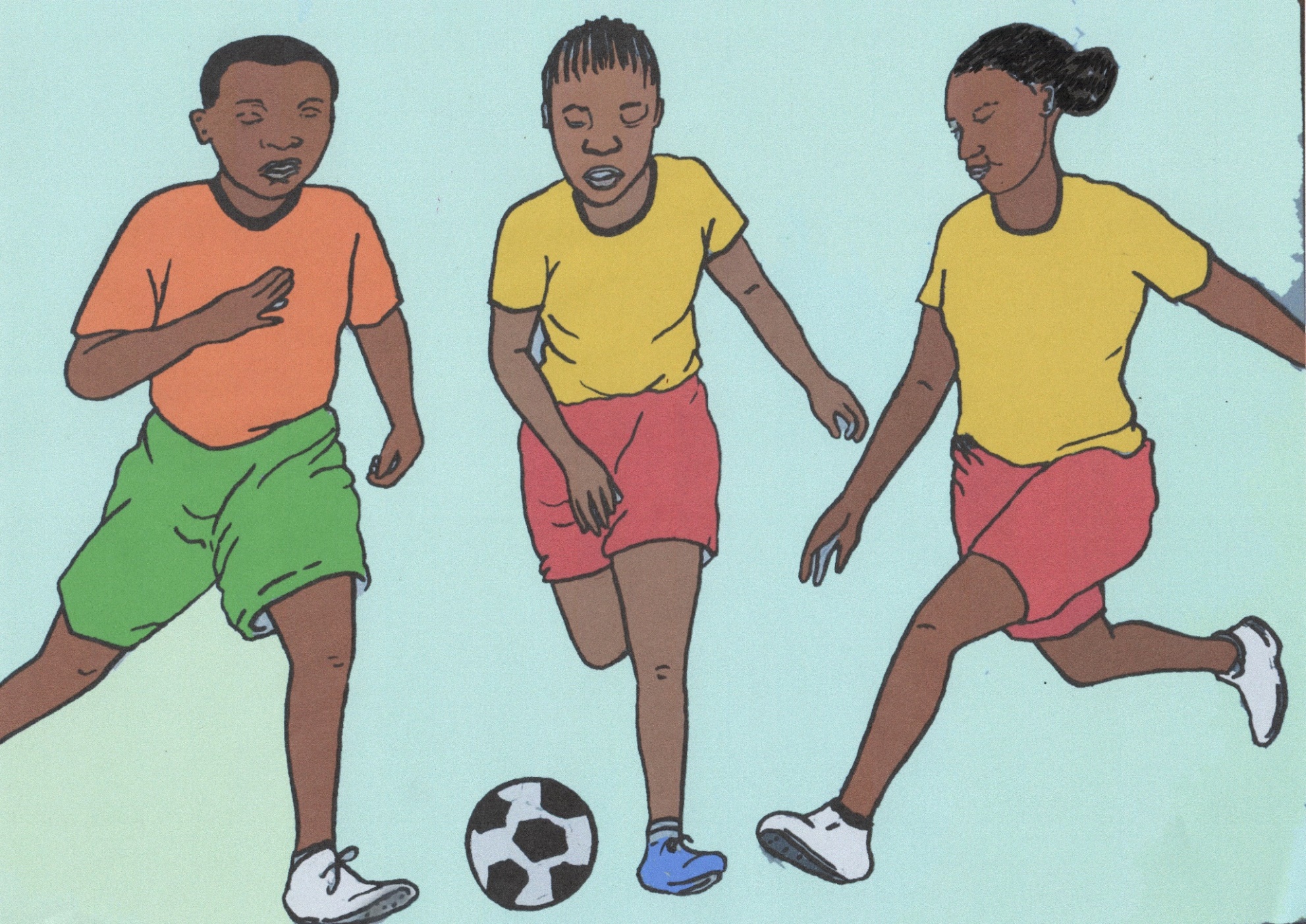


| **A – ACTIVITIES** | | **ZOCHITA** |
| --- | --- | --- |
| **1** | Do you have friends and where do they come from? | Kodi muli ndi anzanu ndipo amachokera kuti? |
| **2** | What types of games do you usually play? | Ndi masewera anji omwe mumakonda kusewera? |
| **3** | Normally who do you play with? | Nthawi zambiri mumasewera ndi ndani? |
| **4** | What religion do you belong to? | Ndinu achipembedzo chanji? |
| **5** | Do you have time to go for prayers? | Kodi muli ndi nthawi yopita kukapemphera? |
| **6** | Does going to pray give you encouragement and hope? | Kodi kupita kukapemphera kumakupatsani chilimbikitso ndi chiyembekezo? |
| **7** | Do you participate in any other religious activities? | Kodi mumatenga nawo gawo/mbali pa zochitika zina za komwe mumapempherako |


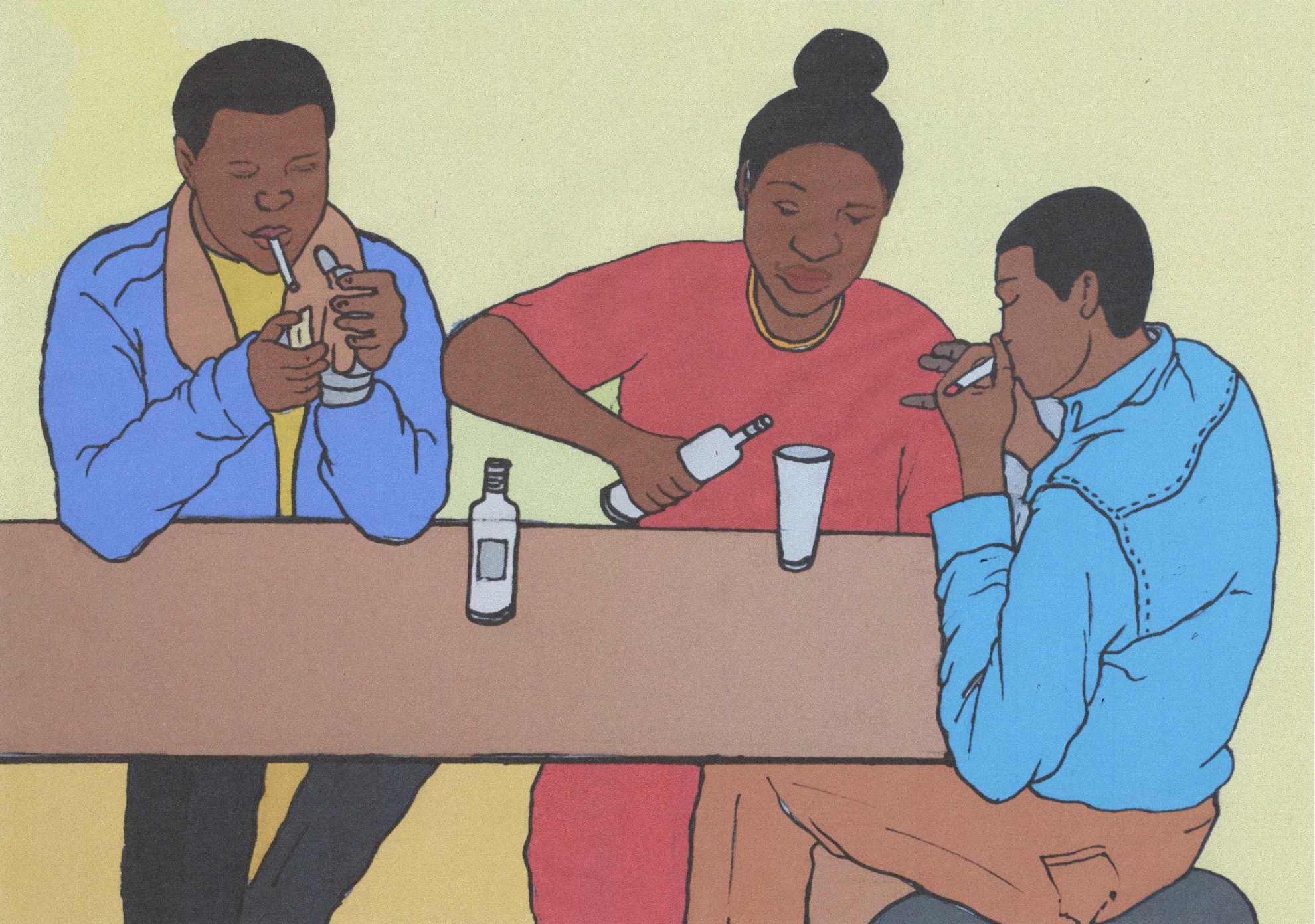


| **D – DRUG USE AND ABUSE** | | **MANKHWALA OZUNGUZA BONGO** |
| --- | --- | --- |
| ***Some young people abuse substances/drugs*** | | ***Achinyamata ena amagwiritsa ntchito mankhwala ozunguza bongo*** |
| 1 | What do you know about substance abuse? | Kodi mukudziwapo chiani za mankhwala ozunguza bongo? |
| 2 | Can you give me examples of addictive drugs found in your community/area? | Kodi mungandipatseko zitsanzo za makhwala ozunguza bongo omwe amapezeka m’dera la kwanu? |
| 3 | Do you have friends who abuse drugs (alcohol, marijuana, cigarettes)? | Kodi muli ndi anzanu omwe amagwiritsa ntchito mankhwala osokoneza bongo (*mowa, chamba, fodya*)? |
| 4 | How about you, have you ever abused drugs? | Nanga inu, munagwiritsapo ntchito mankhwala ozunguza bongo? |
| 5 | Do you know the effects of substance abuse? | Kodi mukudziwa zotsatira za kugwiritsa ntchito mankhwala ozunguza bongowa? |
| 6 | Do you know the negative effects of combining such drugs with ARVs? | Kodi mukudziwa kuipa kophatikiza mankhwalawa ndi ma ARV? |
| 7 | Why do people abuse drugs? | N’chifukwa chiyani anthu amagwiritsa ntchito mankhwala ozunguza bongowa? |
| 8 | How can one avoid abusing drugs? | Kodi munthu angapewe bwanji kugwiritsa ntchito mankhwala ozunguza bongowa? |
| 9 | What advice would you give to your friends who are abusing these drugs? | Kodi mungapatse malangizo otani kwa anzanu omwe amakonda kugwiritsa ntchito mankhwala osokoneza bongo? |


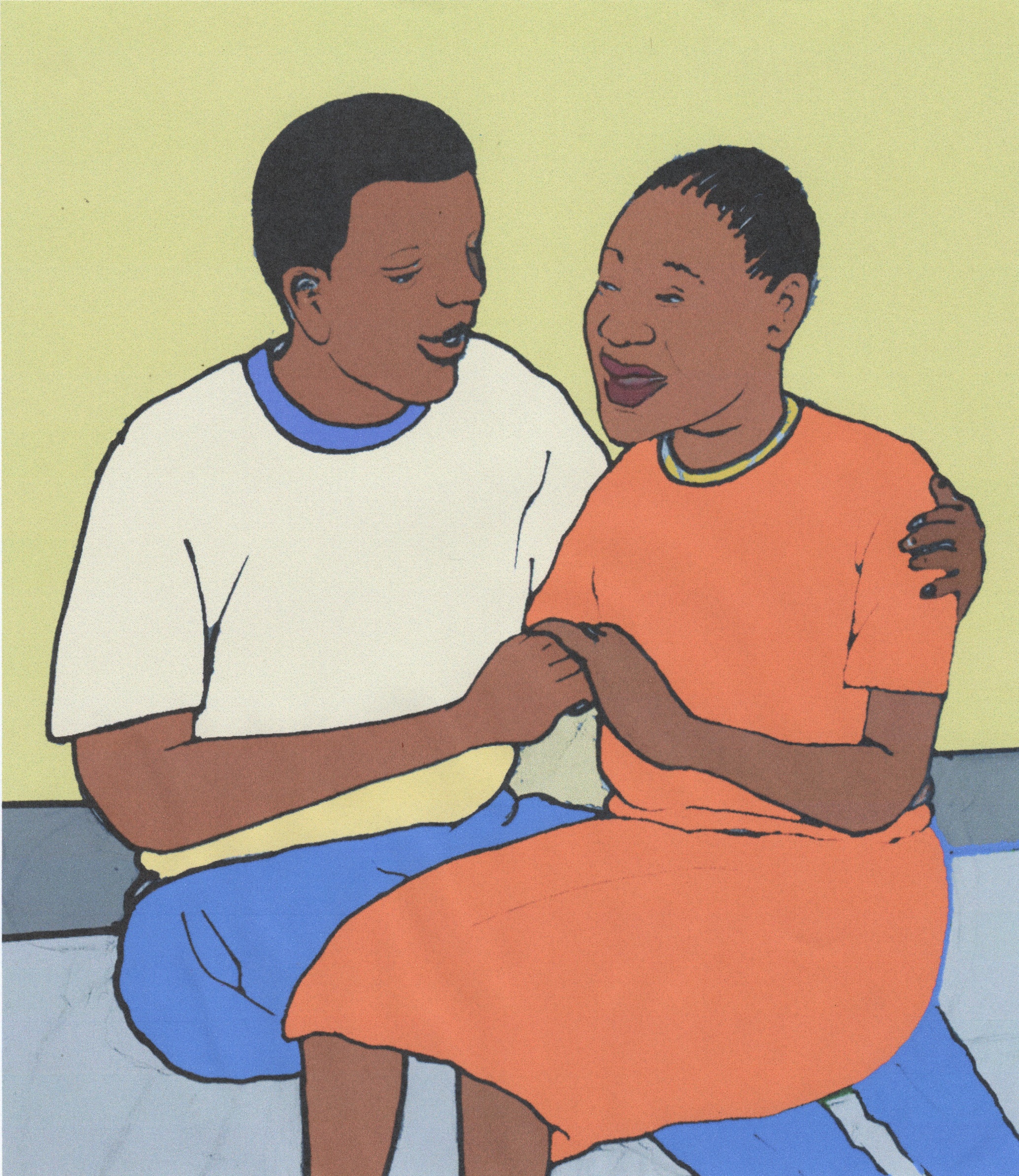


| **S-SEXUAL AND REPRODUCTIVE HEALTH** | | **KUGONANA NDI UBELEKI WABWINO** |
| --- | --- | --- |
| ***Some young people get involved in romantic and sexual relationships*** | | ***Achinyamata ena amatenga nawo mbali m’zibwenzi ndi kugonana*** |
| **1** | Have you ever been asked to date someone? (*If not skip to 4*) | Kodi munafunsidwapo kuti mukhale pachibwenzi?*Ngati ai, funsani nambala 4)* |
| **2** | If yes, was that person same age as you or older than you? | Ngati inde, kodi munthu ameneyo anali wamsinkhu wofanana ndi inu kapena wamkulu kuposa inu? |
| **3** | If you've been in a relationship before, how did you feel about it? | Ngati munayamba mwakhalapo pachibwenzi, zinakusangalatsani bwanji? |
| **4** | If you have never been in a relationship before, has someone ever touched your private parts? | Ngati simunakhalepo pachibwenzi, pali wina amene anakugwirapo ziwalo zobisika? |
| **5** | Have you ever heard of anyone among your friends having a sexual relationship? | Munayamba mwawamverapo anzanu ena kuti amagonana ndi chibwenzi chawo? |
| **6** | How about you, have you ever had any sexual relationship with someone else? (*If not skip to no.14*) | Nanga inu, munayamba mwagonanapo ndi munthu wina?(*Ngati ai funani nambala 14*) |
| **7** | If you have had a sexual relationship with someone else, how many sexual partners have you had? | Ngati munagonanapo ndi munthu wina, kodi munagonanapo ndi anthu angati? |
| **8** | Have you ever exchanged sex with money or material things? | Munayamba mwagonanapo ndi munthu ndi cholinga choti akupatseni ndalama kapena zinthu zimene mumasowa? |
| **9** | If you have ever had a sexual partner, have you ever discussed about HIV and AIDS? | Ngati muli kapena munakhala ndi chibwenzi chogonana nacho, munayamba mwakambiranapo za HIV ndi Edzi? |
| **10** | Have you ever discussed that you are HIV positive and that you are taking ARVs? | Kodi mudakambiranapo kuti muli ndi kachilombo ka HIV komanso kuti mukumwa ma ARV? |
| **11** | If you are in a relationship, has your boy/girlfriend informed you about his/her HIV status or not? | Ngati muli ndi chibwenzi, nanga abwenzi anu anakuwuzani ngati ali ndi kachilombo koyambitsa HIV kapena ayi? |
| **12** | Have you ever accessed information on safer sex? | Kodi mudamvapo nkhani yogonana modziteteza? |
| **13** | Have you ever discussed with your sexual partner about protecting yourselves from STIs and pregnancy? | Munayamba mwakambiranapo ndi chibwenzi chanu zodziteteza ku matenda opatsirana pogonana komanso kutenga mimba? |
| **14** | What kinds of birth control or protection against sexually transmitted diseases do you and/or your partner use? | Ndi njira zotani zolerera kapena zodzitetezera ku matenda opatsirana pogonana zomwe inu ndi/kapena okondedwa anu mumagwiritsa ntchito? |
| **15** | Even if you say you have never been in a relationship do you have an interest in boys or girls? | Ngakhale mwanena kuti simunakhalepo ndi chibwenzi kodi muli ndi chidwi ndi anyamata kapena atsikana? |
| **16** | Are you thinking about marriage in the future? | Kodi mumaganiza zodzakhala pa banja m'tsogolomu? |


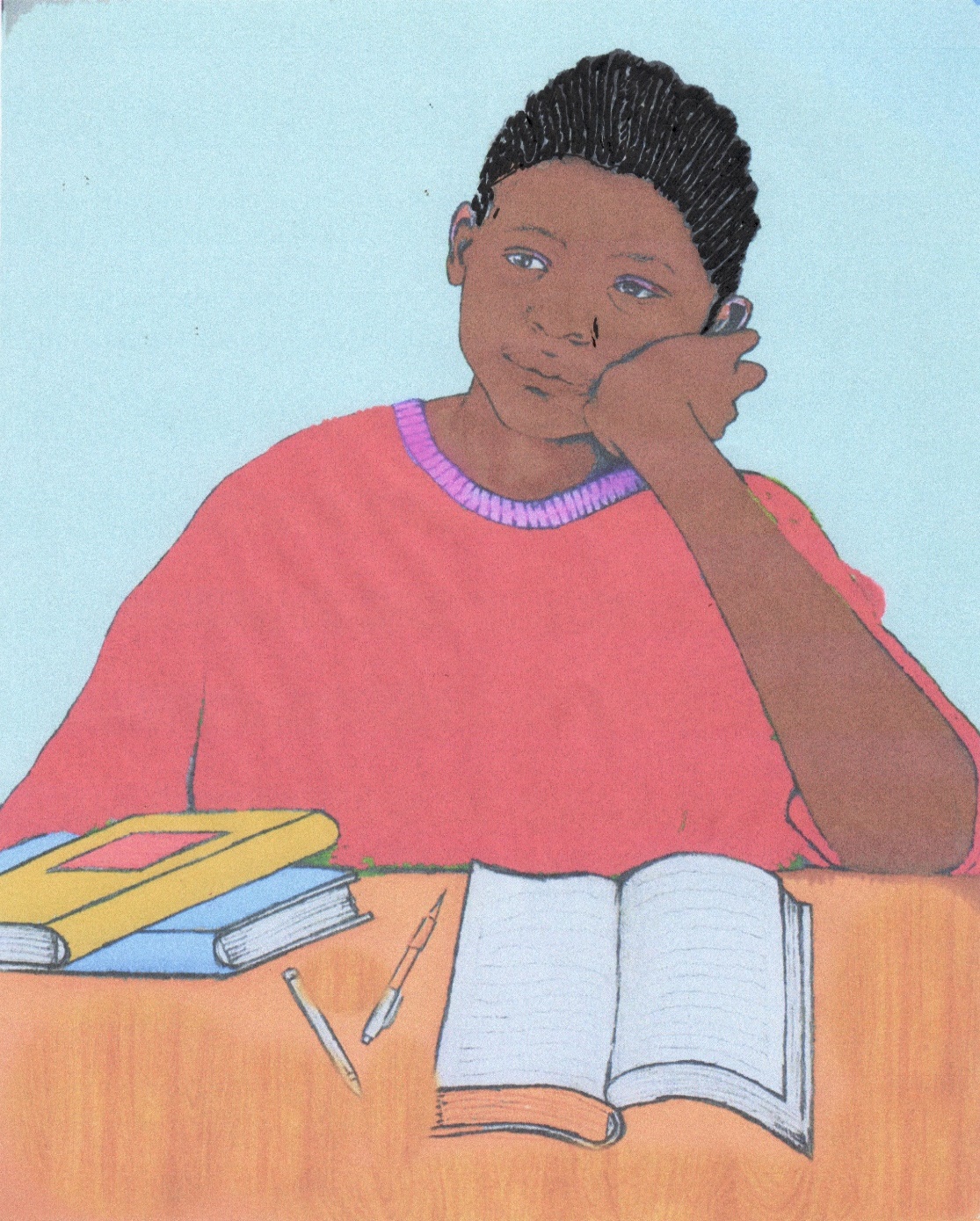

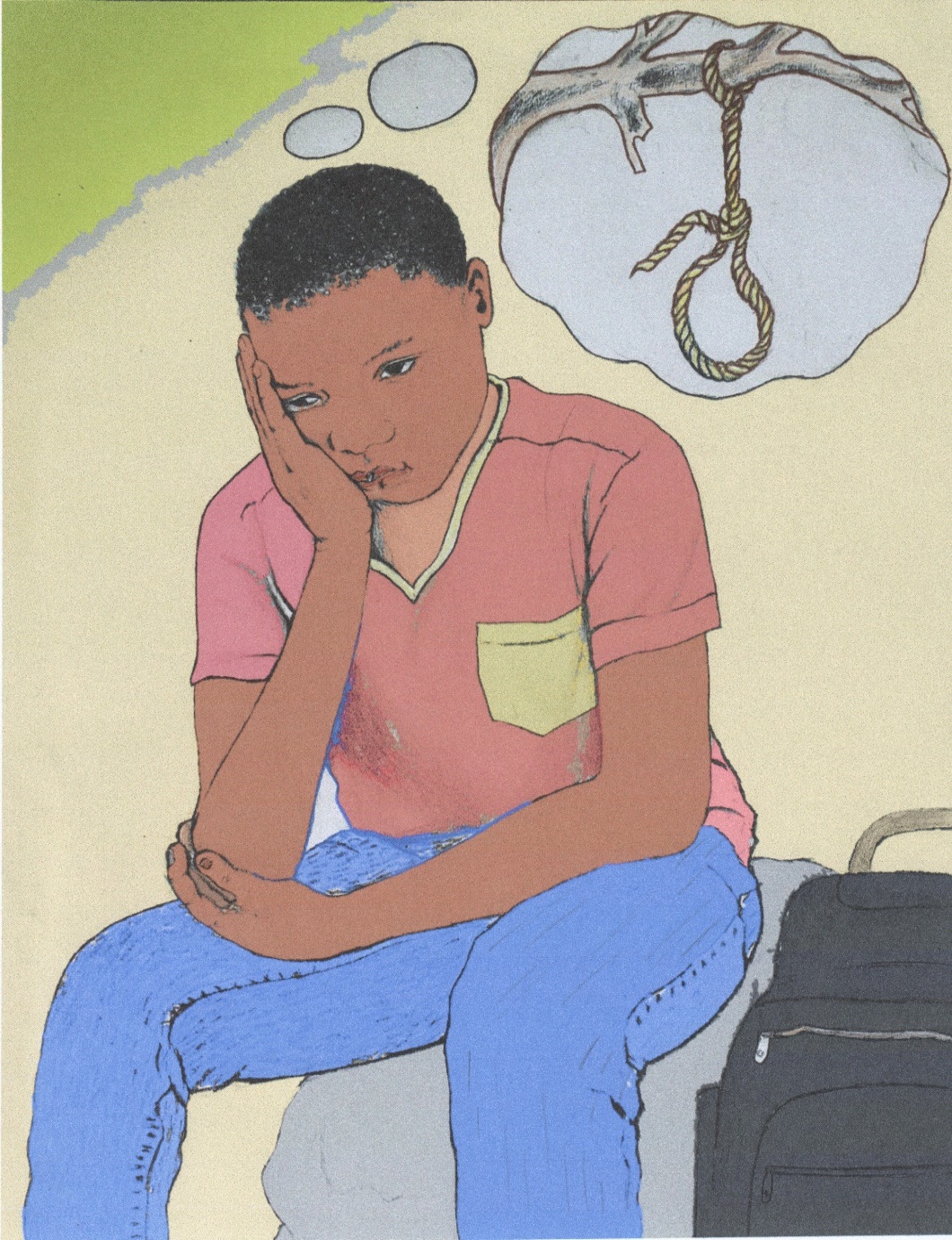


| **S–SUICIDAL IDEATION AND DEPRESSION** | | **KUDZIPHA NDI KUKHUMUDWA** |
| --- | --- | --- |
| ***Sometimes people get stressed up by what is happening around them, have you ever been stressed up?*** | | ***Nthawi zina munthu amatha kukhala okhumudwa ndi zochitika za m’mene akukhalira ndi achibale ake. Inu munayamba mwakhumudwapo komanso kuda nkhawa?*** |
| **1** | Do you know what anxiety is? (*If no, please explain*) | Kodi mumatha kuzindikira kuti nkhawa ndi chiani?(*Ngati asakudziwa, mufotokozeleni)* |
| **2** | Have you ever felt disappointed and worried by the way your family or community members treat you? | Kodi munayamba mwakhumudwapo ndi kuda nkhawa ndi mmene achibale anu kapena anthu a m’dera lanu akukuchitirani? |
| **3** | If yes, please tell me what you are worried about? | Ngati inde, tandiuzani mukuda nkhawa ndi chani? |
| **4** | In particular, what makes you anxious? | Makamaka, chimakupangitsani kuti mukhale ndi nkhawa ndi chiani? |
| **5** | May you explain what can cause a person to be depressed or anxious? | Tafotokozani zomwe zingapangitse munthu kuti akhale okhumudwa kapena ndi nkhawa? |
| **6** | When you get worried, what do you do? | Mukakhala ndi nkhawa mumatani? |
| **7** | Who do you talk to at home when you are worried? | Mumafotozera ndani kunyumba mukakhala ndi nkhawa? |
| **8** | Is there a time when you feel lonely? | Kodi pali nthawi yomwe muli nokha ndipo mumamva bwanji mukakhala nokha? |
| **9** | Have you ever intentionally hurt yourself? | Kodi munadzivulaza mwadala? |
| **10** | If yes, how did you do it? | *Ngati inde, munachita bwanji?* |
| **11** | How do you feel about taking ARVs for the rest of your life? | Kodi mumamva bwanji kuti mukumwa ma ARV m'moyo mwanu wonse? |
| **12** | Have you ever thought that you are different from your peers and that things are not going well because you are on ARVs? | Munayamba mwaganizapo kuti ndinu osiyana ndi anzanu ndipo zinthu sizikuyenda bwino chifukwa mumamwa ma ARV? |
| **13** | Are you able to talk to your friends about the challenges you are facing, and how do they help you? | Kodi mumatha kukambirana ndi anzanu za zovuta zomwe mukukumana nazo ndipo amakulimbikitsani bwanji? |
| **14** | Have you ever felt depressed after an argument with family and friends? Why was that? | Kodi munayamba mwakhumudwapo pambuyo pokangana ndi achibale kapena anzanu? N’chifukwa chiyani zinali choncho? |
| **15** | Have you ever heard of a young person committing suicide and why did he/she do it? | Kodi munamvapo kuti wachinyamata wadzipha ndipo chifukwa chiyani anadzipha? |
| **16** | What are some of the contributing factors that might lead to suicide? | Kodi zina mwa zinthu zimene zingachititse kuti munthu adziphe ndi ziti? |
| **17** | If you were having such thoughts or intentions, what can you do? | Kodi ngati mungakumane ndi maganizo otere, mungatani? |
| **18** | Where can you go and seek help? | Kodi mungapite kuti kukafuna chithandizo? |
| **19** | Who would you tell about such thoughts? | Kodi mungamuuze ndani za maganizo amenewa? |
