# Supplementary material for: Cultural adaptation of a psychosocial screening tool for adolescents living with HIV/AIDS attending antiretroviral therapy program in Malawi
Source: PLoS One. 2025 Nov 17;20(11):e0318738. doi: 10.1371/journal.pone.0318738 (PMC12622793; doi:10.1371/journal.pone.0318738)
Supplement: S1 File — English Focus Group Discussion Guide. S2 Text. Chichewa Focus Group Discussion Guide. S3 Text. Original HEADSS tool. S4 Text. Participants HEADSS adaptation notes_v1. S5 Text. HEADSS adaptation v1. S6 Text. Participants HEADSS adaptation notes_ v2. S7 Text. HEADSS adaptation v2. S8 Text. HEADSS adaptation v3. S9 Text. HEADSS adaptation _v4_Final Version. (ZIP) [file pone.0318738.s001.zip › Supporting Information/Supplementary File 3.doc]

Supplementary File 3 - Original HEADSS Psychosocial Assessment Tool

## H-HOME

- Where do you live?
- Who do you live with?
- How much time do you spend at home?
- What do you and your family argue about?
- Can you go to your parents with problems?
- Have you ever run away from home?

## E-EDUCATION

- What grade are you in?
- What grades are you getting? Have they changed?
- Have you ever failed any classes or been kept back a grade?
- Do you ever cut classes?
- Have you ever been teased or attacked at school?
- Do you work after school or on weekends?
- What are your career/vocational goals?

## A-ACTIVITIES

- What do you do for fun?
- What activities do you do during and after school?
- Are you active in sports? Do you exercise?
- Who do you do fun things with?
- Who are your friends?
- Who do you go to with problems?
- What do you do on weekends? Evenings?

## D-DRUGS

- Do you drink coffee or tea?
- Do you smoke cigarettes? Have you ever smoked one?
- Have you ever tried alcohol? When? What kind and how often?
- Do any of your friends drink or use drugs?
- What drugs have you tried? Have you ever injected steroids or drugs?
- When? How often do you use them?
- How do you get money to pay for drugs?
- Are drugs used or available in places where you hang out?

## S-SEXUAL ACTIVITY/IDENTITY

- Do you feel you are ready for sex?
- Have you chosen to remain abstinent?
- Have you ever had sex?
- How many sexual partners have you had?
- How old were you when you first had sex? How old was your partner?
- Have you ever had sex with men? Women? Both?
- Do you think you might be lesbian, gay, or bisexual?
- Do you think you need to have sex to find out if you're lesbian, gay, or bisexual?
- Do you want to become pregnant? Have you ever been pregnant?
- Have you ever had an infection as a result of having sex?
- Do you use condoms or another form of contraception for STD and HIV prevention?
- Have you ever had sex unwillingly?
- Have you ever tried sex for money, drugs, clothes, or a place to stay?
- Have you ever been tested for HIV? Do you think it would be a good idea to be tested?

## S-SUICIDE/DEPRESSION

- How do you feel today, on a scale of 0 - 10 (0 = very sad, 10 = very happy)?
- Have you ever felt less than a 5? How long did that feeling last?
- What made you feel that way?
- Does thinking you may be lesbian, gay, or bisexual make you feel that way?
- Did you ever think about hurting yourself or that life isn't worth living, or hope that when you go to sleep you won't wake up?

CohenE,MackenzieRG,YatesGL.HEADSS,apsychosocialriskassessmentinstrument: implications for designing effective intervention programs for runaway youth. J Adolesc Health. 1991;12:539-544.
