# Supplementary material for: Cultural adaptation of a psychosocial screening tool for adolescents living with HIV/AIDS attending antiretroviral therapy program in Malawi
Source: PLoS One. 2025 Nov 17;20(11):e0318738. doi: 10.1371/journal.pone.0318738 (PMC12622793; doi:10.1371/journal.pone.0318738)
Supplement: Attachment — Submitted filename: Response to Reviewers_PONE-D-24-58777R1.docx [file pone.0318738.s004.docx]

**Authors Response to Reviewers**

P. O Box 212

Zomba

Malawi

15^th^ July, 2025

**To:**

Academic Editor
PLOS ONE

Dear Academic Editor Prof Olashore,

**Ref: Re-submission ID PONE-D-24-58777R1:** Cultural adaptation of a psychosocial screening tool for adolescents living with HIV/AIDS attending antiretroviral therapy program in Malawi

**Authors:**

Esther C. Kip ([esther_kip@yahoo.com](mailto:esther_kip@yahoo.com))

Mina C. Hosseinipour ([mina_hosseinipour@med.unc.edu](mailto:mina_hosseinipour@med.unc.edu))

Kazione Kulisewa ([kkulisewa@kuhes.ac.mw](mailto:kkulisewa@kuhes.ac.mw))

Brian W. Pence (bpence@unc.edu)

Michael Udedi ([mphatsoudedi@yahoo.co.uk](mailto:mphatsoudedi@yahoo.co.uk))

Bradley N. Gaynes (bgaynes@med.unc.edu)

Vivian F. Go ([vgo@live.unc.edu](mailto:vgo@live.unc.edu))

**Version 2: Date:15/07/2025**

To the Academic Editor:

Once again, we are very grateful to the editors and reviewers for their positive and constructive suggestions. We feel that the quality of the revised manuscript has been improved significantly as a result. All comments in the manuscript file have been taken into account. We have followed each of the Editor’s recommendations for the revised submission, including highlighting in yellow our modifications

**ACADEMIC EDITOR**

Please include the reason for using a purposive sampling method and highlight the justification for your sample size

**Response:** Thank you for your suggestion. We have included the rationale for using purposive sampling method and highlighted the justification for the sample size in the manuscript as shown in lines 141 - 158, p. 6 and 7

Could the sampling method be a limitation of this study?

**Response:** Thank you for pointing out this issue. Yes, it might be a limitation of this study due to limited generalizability of the findings and selection bias of participants. We have included this in the Study Limitation section (see lines 711 – 715, pgs. 32 & 33).

Also, kindly make the abstract section brief but comprehensive

**Response:** Thank you for your valuable feedback. We have revised the abstract section to be more concise, reducing its length from 299 words to 242 words (see lines 24 - 50, pgs. 1&2).

Please review your reference list to ensure that it is complete and correct. If you have cited papers that have been retracted, please include the rationale for doing so in the manuscript text, or remove these references and replace them with relevant current references. Any changes to the reference list should be mentioned in the rebuttal letter that accompanies your revised manuscript. If you need to cite a retracted article, indicate the article’s retracted status in the References list and also include a citation and full reference for the retraction notice.

**Response:** We appreciate this important point. Upon thorough review, no explicit evidence of retracted articles was found among the cited references but we have revised reference No.55 to read “Government of the Republic of Malawi. National Commission for Science and Technology. National policy measures and requirements for the improvement of health research co-ordination in Malawi. 2012 revised edition. Lilongwe. Malawi”. We have also highlighted it in the reference list. However, we acknowledge the possibility of oversight. We would be grateful if the editors could indicate any specific retracted articles they have identified.

**REVIEWER 1 COMMENTS**

**Reviewer comment:** 1. Lines 253–260: I recommend deleting this section, as it reiterates points already made earlier in the manuscript.

**Response:** We thank you for your suggestion. The information in lines 253 -260 has been deleted.

**Reviewer comment:** 2. Lines 321–325: Please revise the reference to the supplemental materials to read: “see Supplemental Information S3–S9.”

**Response:** Thank you for your valuable feedback. We have revised the reference to the supplemental materials to read as suggested (see lines 325 – 326, p.16).

**Reviewer comment:** 3. Lines 337–339: This section needs clarification. Elsewhere in the manuscript, it appears that suicide-related questions were included. Was there a period during which these questions were removed? This does not seem to be the case based on the supplemental materials.

**Response:** Thank for your feedback. As recommended, we have included some clarification on the questions which were removed/changed from the original HEADSS tool during the adaptation process as shown in lines 337 – 347, p. 15 &16.

**Reviewer comment:** 4. Lines 337–339: I also recommend adding a comment—perhaps in the Discussion—on the implications of removing sexual orientation questions. Consider addressing how stigma and psychological distress among sexual minorities in Malawi may influence HIV transmission, treatment adherence, risky sexual behaviors, and increased risk of mental health disorders among sexual minority individuals living with HIV.

**Response:** Thank you for pointing this out. We have included the suggested information in the Discussion section as highlighted in yellow in lines 610-638, pgs. 28– 29.

**Reviewer comment:** 5. Lines 382–387: The addition of a section on suicide in the Discussion is valuable. I suggest expanding this section, particularly by contrasting participants’ generally supportive views on asking about suicide with healthcare workers’ concerns that such questions may heighten suicide risk. This dichotomy is worth further exploration—specifically, how prevalent this attitude gap is in the existing literature. The authors should also consider discussing how this divergence in perspectives may impact suicide screening and prevention efforts, and how it highlights the need for interventions—such as provider training and public health messaging—to shift healthcare workers’ attitudes toward alignment with evidence-based best practices, especially for adolescents living with HIV.

**Response:** Thank you again for your valuable suggestion. We have included the suggested information in the Discussion section as highlighted in yellow (see lines 645 – 651 & 660 – 674, pgs. 30 & 31).

**Reviewer comment:** 6. The manuscript contains multiple sections that repeat similar ideas (e.g., discussion of acceptability, relevance, and comprehensibility is revisited with overlapping quotes and interpretations). Consider condensing.

**Response:** We appreciate your suggestion. We have incorporated your suggestions in the results section as shown in line 328, we have combined the section on Comprehensibility and Relevance of the adapted tool and all section and subsections in Acceptability section have been combined as shown in line 421.
